# Design, Semisynthesis, and Estrogenic Activity of Lignan Derivatives from Natural Dibenzylbutyrolactones †

**DOI:** 10.3390/ph15050585

**Published:** 2022-05-09

**Authors:** Priscila López-Rojas, Ángel Amesty, Miguel Guerra-Rodríguez, Yeray Brito-Casillas, Borja Guerra, Leandro Fernández-Pérez, Ana Estévez-Braun

**Affiliations:** 1 Departamento de Química Orgánica, Instituto Universitario de Bio-Orgánica, Universidad de La Laguna, Avda. Astrofísico Fco. Sánchez 2, 38206 La Laguna, Tenerife, Spain; plopezro@ull.edu.es; 2 Instituto Universitario de Investigaciones Biomédicas y Sanitarias (IUIBS), Universidad de Las Palmas de Gran Canaria (ULPGC), Paseo Blas Cabrera Felipe s/n, 35016 Las Palmas de Gran Canaria, Spain; miguel.guerra106@alu.ulpgc.es (M.G.-R.); yeray.brito@ulpgc.es (Y.B.-C.); borja.guerra@ulpgc.es (B.G.)

**Keywords:** natural products, lignans, estrogenic and antiestrogenic activities, induced fit docking (IFD), molecular dynamics (MD)

## Abstract

Based on molecular docking studies on the ERα, a series of lignan derivatives (**3**–**16**) were designed and semisynthesized from the natural dibenzylbutyrolactones bursehernin (**1**) and matairesinol dimethyl ether (**2**). To examine their estrogenic and antiestrogenic potencies, the effects of these compounds on estrogen receptor element (ERE)-driven reporter gene expression and viability in human ER+ breast cancer cells were evaluated. Lignan compounds induced ERE-driven reporter gene expression with very low potency as compared with the pure agonist E2. However, coincubation of 5 μM of lignan derivatives **1**, **3**, **4**, **7**, **8**, **9**, **11**, **13**, and **14** with increasing concentrations of E2 (from 0.01 pM to 1 nM) reduced both the potency and efficacy of pure agonists. The binding to the rhERα-LBD was validated by TR-FRET competitive binding assay and lignans bound to the rhERα with IC_50_ values from 0.16 μM (compound **14**) to 6 μM (compound **4**). Induced fit docking (IFD) and molecular dynamics (MD) simulations for compound **14** were carried out to further investigate the binding mode interactions. Finally, the in silico ADME predictions indicated that the most potent lignan derivatives exhibited good drug-likeness.

## 1. Introduction

Lignans are a large group of natural products derived from the shikimic acid biosynthetic pathway. They are formed by a β-β′ (8-8′) linkage between two phenylpropane units (C6-C3) with a different degree of oxidation in the side-chain and a different substitution pattern in the aromatic moieties [1]. On the basis of their structural patterns, including the way in which oxygen is incorporated into the skeletons, their carbon skeletons and the cyclization pattern lignans are classified into eight groups (arylnaphthalene, aryltetralin, dibenzocyclooctadiene, dibenzylbutane, dibenzylbutyrolactol, dibenzyl butyrolactone, furan, and furofuran) [1,2]. They exhibit a wide range of biological activities including antitumor activity [3,4,5], antiviral activity [6], antimitotic activity [7,8], inhibition of the activity of enzymes such as cAMP phosphodiesterase [9] and cytochrome oxidase [10], anti-inflammatory activity [11], hypolipidemic activity [12], and estrogenic activity [13]. Regarding the estrogenic activity, lignans together with isoflavones, stilbenes, and coumestans constitute the major groups of phytoestrogens [14,15].

These compounds are considered to be natural selective estrogen receptor modulators (SERMs), since they induce estrogenic and/or antiestrogenic effects by weakly binding to estrogen receptors (ER), competing with 17β-estradiol (E2) for the ligand-binding domain (LBD) of the ER [16]. Accordingly, lignans can interact with ER and modulate cellular development and proliferation in different ER-positive (ER+) organs. However, similar to SERM [16], the agonistic and/or antagonistic activities of lignans depend on the tissue, hormonal conditions, and interactions with other cellular pathways [17,18]. Accordingly, lignans can potentially interact with ER and can modulate cellular development and proliferation in normal cells and E2-related disorders such as ER+ cancer (i.e., prostate, ovary, or breast cancers), osteoporosis, neurodegeneration, insulin resistance, or hyperlipidaemia [3,4,12,14,19,20].

Due to our interest in compounds with estrogenic/antiestrogenic activity [13,21], some lignan derivatives were designed and semisynthesized. These compounds were obtained from two natural dibenzylbutyrolactone lignans, namely bursehernin (**1**) and dimethyl ether matairesinol (**2**), isolated from *Bupleurum salicifolium* in large amounts [22]. Chemical screening and functional evaluation of estrogenic/antiestrogenic activities were carried out to validate their interaction with ERα and biological activities. Docking, induced fit docking (IFD) and molecular dynamics (MD) simulations were performed in order to propose a possible binding mode with ER. Finally, in silico ADME predictions indicated that the most potent lignans exhibited good drug-likeness.

## 2. Results and Discussion

### 2.1. Chemistry

**Figure 1:**
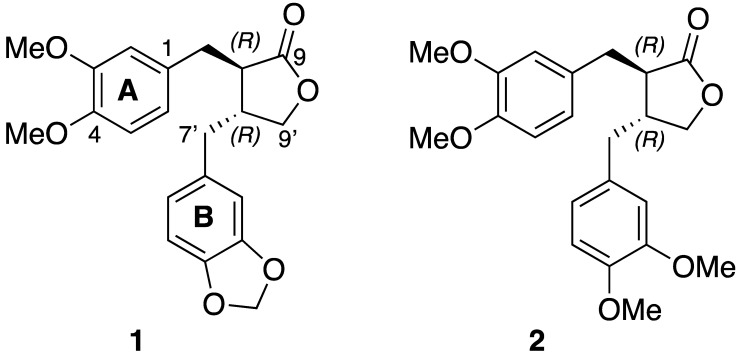
Structures of bursehernin **1** and dimethyl ether matairesinol **2**.

**Figure 2:**
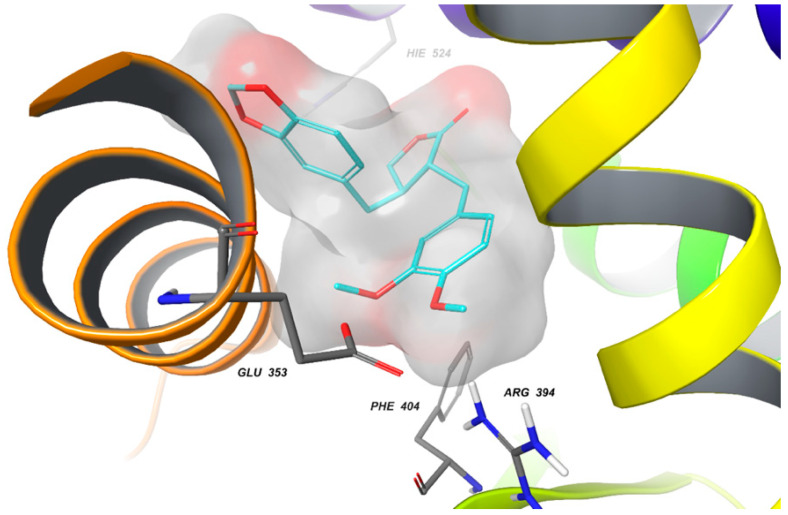
Docking of bursehernin **1** into ERα (PDB 3ERT).

The structures of bursehernin (**1**) and dimethyl ether matairesinol (**2**), are shown in Figure 1. These dibenzylbutyrolactones, in preliminary docking studies, fit well into the binding pocket of the Erα through hydrophobic interactions (Figure 2). Some key hydrogen bond interactions with the residues Glu353 and Arg394 as well as the π-stacking interaction with Phe 404, which are present in the most part of SERMs, are not observed. Probably, the presence of the methoxy groups in the ring A prevents the mentioned interactions while the existence of the methylendioxy group in the ring B avoids the formation of another representative hydrogen bond interaction with His524.

**Figure 3:**
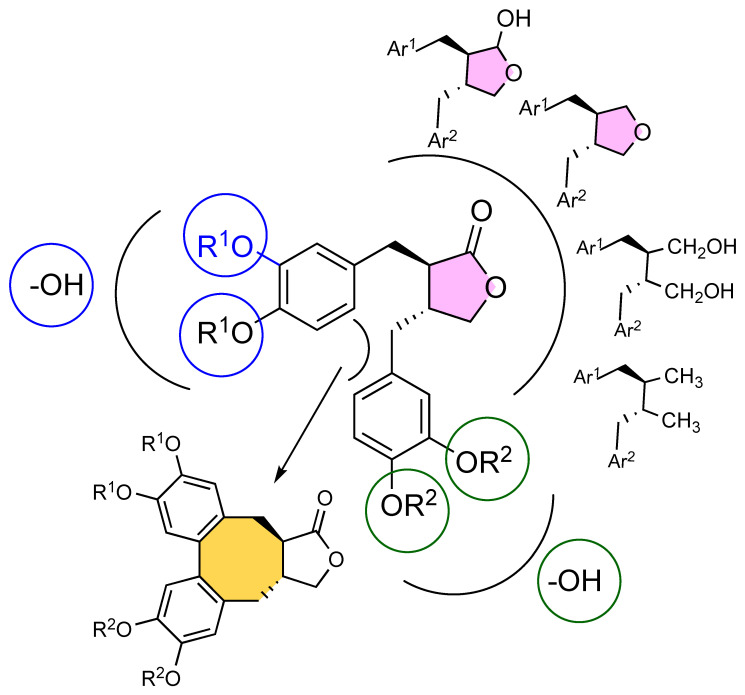
Designed modifications.

Aiming to obtain better affinities, a series of designed modifications were planned (Figure 3). The first designed modification was the replacement of the methoxy and the methylenedioxy groups with hydroxyl groups, since in docking studies the presence of these hydroxyl groups established hydrogen bond interactions. Thus, for instance, the tetrahydroxylated derivative **3** presented a docking score value of −11.58 kcal/mol and showed three hydrogen bond interactions between the hydroxyl groups at the A ring and the carbonyl group of the residue Glu353 and the imine group of the residue Arg394, and one hydrogen bond interaction between one of the hydroxyl groups at the B ring and the residue Thr347 (Supplementary Material). The next modifications were focused on the lactone ring. We reduced the lactone moiety to lactol and to tetrahydrofuran, and also opened the lactone to obtain dibenzyl butane-type lignans with higher conformational freedom. These transformations would allow us to see the role of the carbonyl group and the influence of different connectors of the dibenzyl units in the estrogenic activity. Docking studies on some of the derivatives to be obtained revealed good affinities toward the ERα. For instance, the hydroxylated dibenzylfuran-type lignan (**11**) had a docking score value of −10.87 kcal/mol. This compound exhibited three hydrogen bond interactions between the hydroxyl groups at the B ring and the carbonyl group of the residue Glu353 and Leu387, and the imine group of the residue Arg394 (Supplementary Material). The last transformation performed through a conjunctive approach was the conversion of the dibezylbutyrolactones into dibenzocyclooctadiene-type lignans to introduce conformational constraint.

**Scheme 1:**
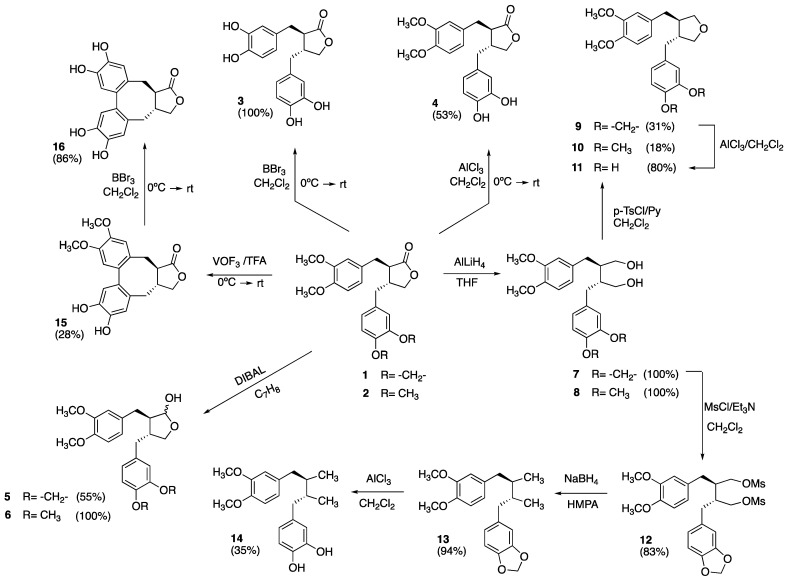
Semisynthesis of derivatives (**3**–**16**).

Based on the designed modifications mentioned above, the transformations shown in Scheme 1 were carried out. Thus, when bursehernin (**1**) was treated with boron tribromide [23,24], the tetrahydroxylated derivative (**3**) was quantitatively obtained while the use of aluminum trichloride [25] led to the transformation of the methylenedioxy group into the corresponding free hydroxyl groups yielding compound (**4**) in 53% yield. The treatment of bursehernin (**1**) and dimethyl ether matairesinol (**2**) with diisobutyl aluminum hydride (DIBAL) in toluene gave the corresponding mixture of lactol epimers (**5**) (1.0:1.6) and (**6**) (1.0:1.8). In the ^1^H NMR spectra, the hemiacetal proton was located at δ 5.23 for lactol epimers (**5**) and at δ 5.24 for lactol epimers (**6**). When lignans (**1**) and (**2**) were reacted with an excess of AlLiH_4_ in THF, the dihydroxylated dibenzyl butanes (**7**) and (**8**) were quantitatively obtained. The presence of two -CH_2_OH groups was observed in the NMR spectra, thus, for example, two doublet of triplets at δ 3.77 (2H, *J* = 11.4, 2.7 Hz) and at δ 3.49 (2H, *J* = 11.4, 4.8 Hz) were detected in the ^1^H NMR of compound **7**, and two signals at δ 60.4 ppm and δ 60.3 ppm corresponding to C-9 and C-9′ in the ^13^C NMR spectra. Lignans **7** and **8** under treatment with tosyl chloride/py in CH_2_Cl_2_ afforded the furan-type lignans **9** and **10**, respectively, in moderated yields. These compounds showed, in their ^1^H-NMR spectra, the typical signals of the CH_2_ next to the heterocyclic oxygen, for instance, at δ 3.90 (2H, dd, *J* = 8.7, 6.5 Hz, H-9b, H-9′b) and δ 3.52 (2H, dd, *J* = 8.7, 6.0 Hz, H-9a, H-9′a) in the case of compound **10**. Compound **9**, in the presence of AlCl_3_/CH_2_Cl_2_, yielded the dihydroxy derivative (**11**) (80%). The reaction of compounds (**7**) with mesyl chloride gave the corresponding mesylate **12** (83%), which was converted into dibenzyl butane **13** (94%) under treatment with NaBH_4_/HMPA. Compound **13** exhibited, in the ^1^H NMR, the characteristic doublet methyls (*J* = 6.2 Hz) at δ 0.80 and δ 0.82 ppm. The last transformation performed was the conversion of bursehernin (**1**) into dibenzocyclooctadiene (**15**) through a vanadium oxyfluoride mediated oxidative cyclization. Under the reaction conditions, the cleavage of the methylenedioxy group was detected. The treatment of compound **15** with BBr_3_ afforded the tetrahydroxylated derivative (**16**). The presence of four aromatic hydrogens as singlets at δ 6.72 (H-2, H-2′), 6.68 (H-5′), and 6.64 (H-5) for compound **15**, and at δ 6.66 (H-2), 6.64 (H-2′), 6.56 (H-5′), and 6.54 (H-5) for compound **16**, corroborated the corresponding cyclization.

### 2.2. Biological Activity

#### 2.2.1. Lignan Derivatives Modulate ER-Dependent Transcriptional Activity in ER+ Breast Cancer Cells

The analysis of estrogenic and antiestrogenic activities of the lignan derivatives (**1**–**16**) was performed by using stably transfected T47D-KBluc cells, a human ER+ breast cancer cell line which contains an estrogen receptor element coupled to the luciferase reporter gene [26]. Lactols (**5**) and (**6**) were tested as a mixture of the two epimers obtained. In this cell line, maximal ER-dependent transcriptional activity (i.e., Emax) was induced by the pure agonist E2 in a dose-effect dependent manner (EC_50_ = 0.0004 ± 0.0001 nM). This effect was abolished by coincubation with the antiestrogens ICI-182.780 (IC_50_ = 0.0002 ± 0.00008 nM) and 4-hydroxytamoxifen (4-OHTAM) (IC_50_ = 0.38 ± 0.009 μM). The effects of lignans (**1**–**16**) (from1 μM to 10 μM) were analyzed in the absence or in the presence of E2. Table 1 shows that lignan compounds **3**, **5**, **9**, **11**, **12**, and **14** exerted a weak estrogenic activity in the absence of E2 that reached 24% maximal transcriptional activity induced by 0.1 nM E2 (i.e., Emax). In addition, coincubation of E2 with 5 μM of these lignan derivatives enhanced the maximal transcriptional activity induced by E2. Conversely, coincubation of E2 with 5 μM of lignan compounds **8**, **10**, or **16** led to a significant decrease in E2-ERE-luciferase activity. As the previous experiment showed that some lignan derivatives possessed weak estrogenic activity in the absence of E2, selected compounds were coincubated with the pure agonist E2 and a dose-response analysis (from 1 μM to 10 μM) in the presence of 0.1 nM E2 was performed (Table 1). The results revealed that compounds **1**, **7**, **9**, **11**, and **13** decreased the E2-induced ERE-luciferase activity in a dose-dependent manner with calculated IC_50_ values lower than 20 μM (Table 1). Therefore, these results indicate that, in addition to a slight yet significant increment of basal luciferase activity, some lignans can cause reduction of maximal E2-induced ERE activation which suggests that they are partial agonists/antagonists [27]. These data also indicate that these lignan derivatives can exert their effects similar to natural lignans which are considered to be partial agonists/antagonists or SERM-like compounds since they induce estrogenic and/or antiestrogenic effects by weakly binding to ER and competing with E2 for LBD [13,28,29,30]. In contrast, the inhibitory effects of lignan derivatives **1**, **7**, **9**, **11**, and **13** on E2-induced ERE activation in T47DKBluc cells were different from the pure estrogen antagonist ICI-182.780 which did not cause estrogenic effects [31]. Furthermore, the inhibitory effects of lignan derivatives on E2-induced luciferase activity did not involve a reduction in the amount of total protein (data not shown), but rather suggested it caused a specific inhibition of ER-dependent transcriptional activity in the T47D-KBluc cells.

**Figure 4:**
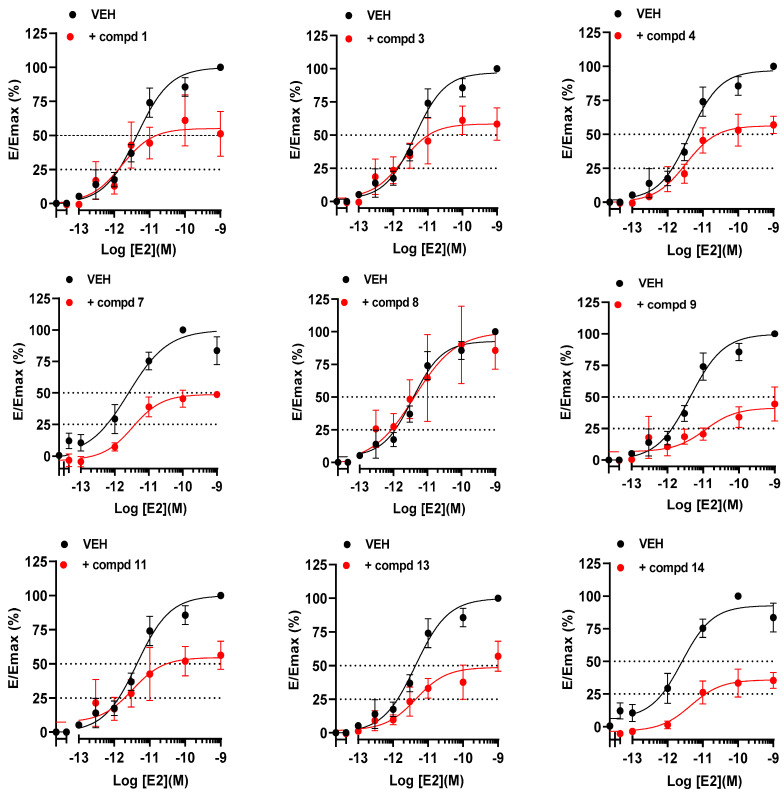
Antagonisms of lignan derivatives on ERE-mediated transcription. T47D-KBRluc cells were seeded in E2-depleted growth media (5%DCC-FBS) and pretreated with lignan derivatives at 5 μM for 3 h followed by increasing E2 concentrations (from 0.01 pM to 1 nM). Thus, the dose-effect relationship of E2 on luciferase activity was tested in the absence (●) or in the presence (●) of lignan derivatives. Luciferase activity (relative luminescence units or RLU) from each sample was measured in a luminescence microplate reader, as described in the Material and Methods. Then, RLUs from each sample were normalized by protein concentration and converted to fold induction with respect to the vehicle-treated control. The maximal increase in luciferase activity or Emax (5.19 ± 1.71 fold induction) was induced by the pure agonist E2 and the efficacy (E) of each treatment, as compared with Emax, was calculated (E/Emax (%)). Data are expressed as mean ± SEM for at least three independent experiments, where each treatment is tested in triplicate.

To further assess the antiestrogenic activity of lignan derivatives, the T47D-KB luc cells were treated with selected compounds at 5 μM for 3 h followed by increasing E2 concentrations (from 0.01 pM to 1 nM). This pretreatment period was enough to detect significant antagonism with compounds **1**, **3**, **4**, **7**, **9**, **11**, **13**, and **14** which caused a significant decrease in potency (i.e., increased EC_50_) and/or maximal E2-ERE-luciferase activity (i.e., Emax) (Figure 4 and Table 2). Interestingly, antagonisms of compounds **3**, **4**, and **14** were not detected when the T47D cells were pretreated for 1 h before addition of 0.1 nM E2 (Table 1), which indicated that they need a longer period than similar lignan derivatives to cause inhibition on E2-induced luciferase activity. Taken together, these findings suggest that lignan derivatives are able to compete with E2 for binding to ER but with lower affinity and efficacy than pure agonists and, therefore, when they are combined with E2, inhibit E2-induced ERE activation.

**Figure 5:**
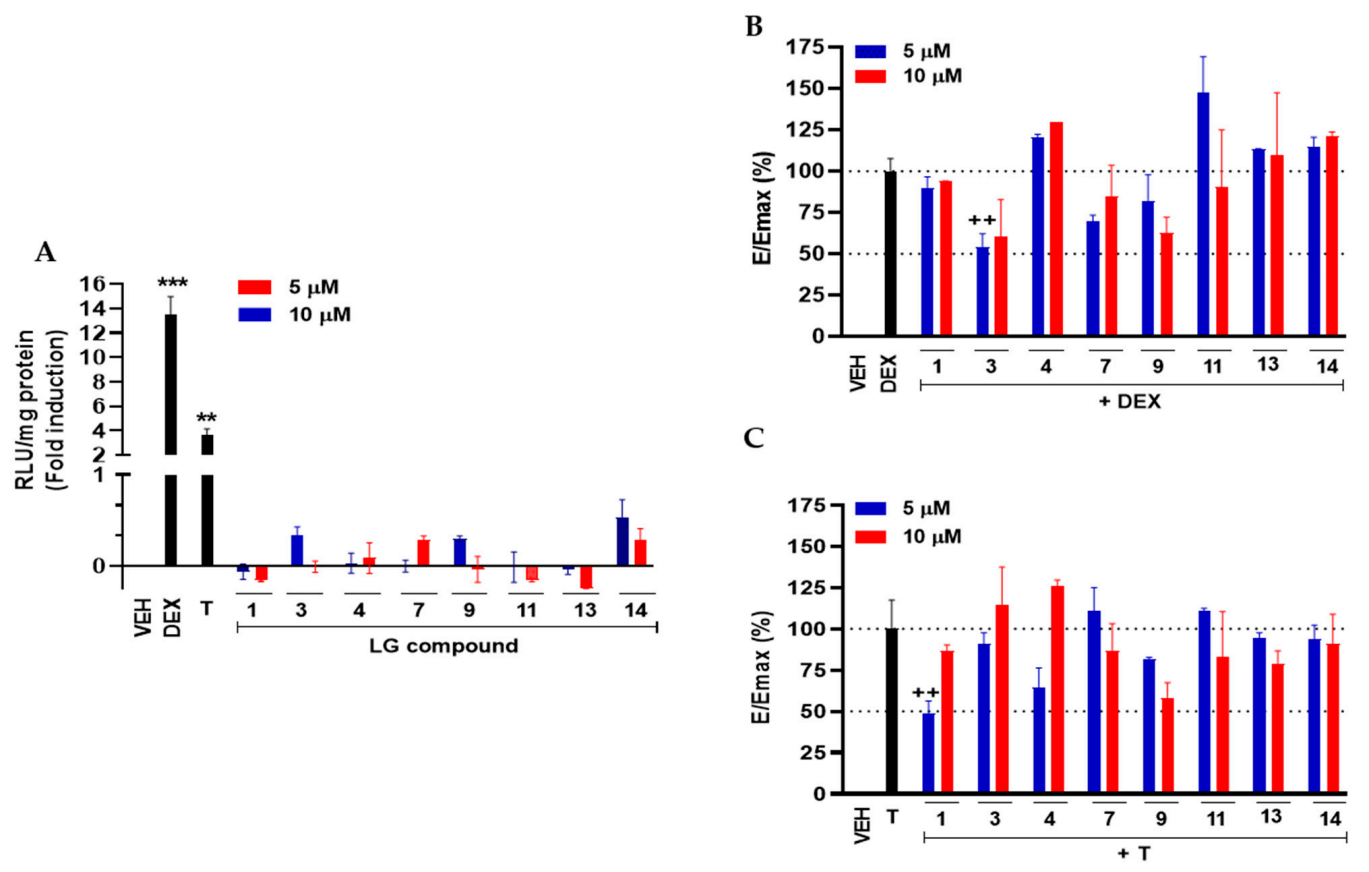
Effects of lignan derivatives on AR- and GR-mediated transcription. MDKBrluc cells were pretreated with lignan derivatives at 5 μM (blue) or 10 μM (red) for 3 h. Then, the cells were treated with vehicle (005% DMSO) (**A**), 0.1 μM testosterone (T) (**B**), or 0.1 μM dexamethasone (DEX) (**C**) for 18 h. Luciferase activity (RLUs) from each sample was measured in a luminescence microplate reader, as described in the Material and Methods. Then, RLUs from each sample were normalized by protein concentration and converted to fold induction with respect to the vehicle-treated control. The maximal increase in luciferase activity (i.e., Emax) was induced by the pure agonists DEX or T and the efficacy (E) of each treatment, as compared with Emax, was calculated (E/Emax (%). Data are expressed as mean ± SEM for at least three independent experiments, where each treatment is tested in triplicate. ** *p* < 0.01 and *** *p* < 0.001 versus VEH-treated cells. ^++^ *p* < 0.01 versus DEX- or T-treated cells.

Since there is high homology among LBD of estrogen (ER), androgen (AR), and glucocorticoid (GR) receptors [16], we further assessed the effects of lignan derivatives on AR and GR-dependent transcriptional activity. The triple negative breast cancer cell line MDA-kb2 [32], was screened with compounds in the absence or presence of 100 nM testosterone (T) or 100 nM dexamethasone (DEX), the lowest concentrations that produced maximal androgenic or glucocorticoid response in MDA-kb2 cells, respectively. Neither, 4OH-TAM (data not shown) nor most of lignans displayed cross-activation on basal luciferase activity (Figure 5A).

Notably, 5 μM of compounds **3** (Figure 5B) and **1** (Figure 5C) caused 50% inhibition of DEX- and T-induced Emax, respectively. However, whether these lignan derivatives can provoke an inhibition of AR- or GR-dependent activities still deserves further research.

#### 2.2.2. Lignan Derivatives Inhibit ER+ Breast Cancer Cells Viability

**Figure 6:**
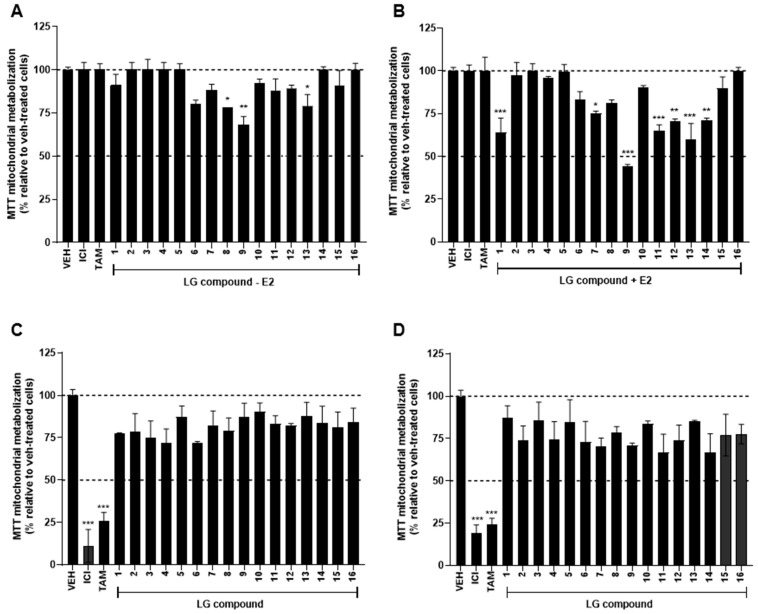
Effects of lignans on ER+ breast cancer cell viability. E2-deprived T47D BC cells were treated with vehicle (VEH, 0.05% DMSO), ICI (1 μM), TAM (10 μM), or lignans (10 μM) for 24 h in the absence (**A**) or in the presence of 0.1 nM E2 (**B**). In addition, E2-non-depleted T47D (**C**) or MCF-7 (**D**) cells were treated with VEH or lignans at 10 μM for 72 h. Then, the mitochondrial metabolization of MTT was used as an indicator of cell viability, as described in the Material and Methods. Data are expressed as mean ± SEM for at least three independent experiments, where each concentration is tested in triplicate. * *p* < 0.05, ** *p* < 0.01, and *** *p* < 0.001 versus VEH-treated cells.

The effects of lignan derivatives were evaluated on human ER+ BC cells. First, E2-depleted T47D cells were exposed to compounds at 10 μM for 24 h. In the absence of E2 (Figure 6A), compounds **1**, **6**, **8**, **9**, **11**, **12**, **13**, and **14** reduced cell viability between 16.6% (compound **6**) and 55.6% (compound **9**). Interestingly, the reduction in T47D cell viability was higher in the presence of E2 (Figure 6B). In addition, treatment of E2 non-depleted T47D (Figure 6C) and MCF7 (Figure 6D) cells with lignans for 72 h only reduced cell viability up to 33.4% (compound **14**). However, this inhibitory effect was potentiated when treatment was prolonged for 5 days (data not shown), a time-dependent effect that suggests the presence of growth-promoting factors that partially prevented the effects of lignans. These data also indicate that the effects of lignan derivatives were related to the level of E2 present in the cell cultured medium and may function via an interference with the effects of E2 on cell viability.

#### 2.2.3. Lignan Derivatives Bind to Recombinant Erα (rhERα)

**Figure 7:**
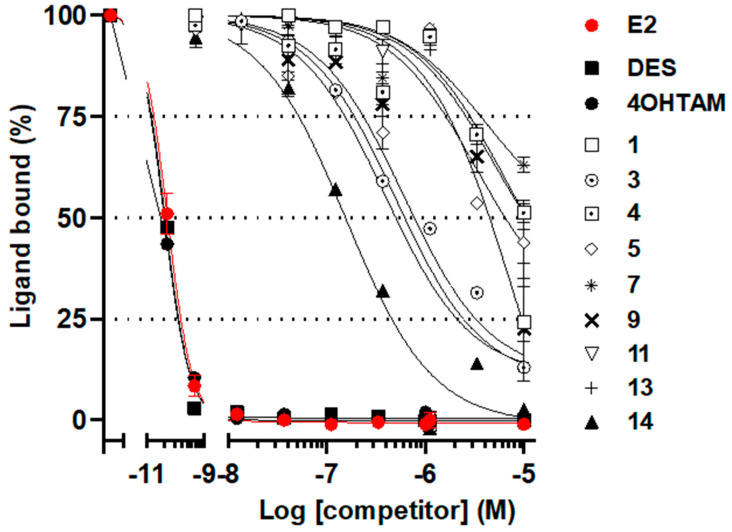
Lignan derivatives bind to the recombinant human Erα (rhERα). The binding of competitors to rhERα was evaluated in 10-point titration competition curves (from 0.01 pM to 20 μM) by using the LanthaScreen TR-FRET binding assay, as described in the Material and Methods. Data are expressed as mean ± SEM for from at least three independent experiments, where each concentration is tested in triplicate.

Next, the binding of lignan derivatives to the LBD of rhERα was analyzed by using time-resolved fluorescence resonance energy transfer (TR-FRET) competitive binding assay [33]. This binding assay uses the competition of selective fluorescent ligand Fluormone^TM^ for rhERα (Kd = 0.23 nM), an E2-like compound. The IC_50_ values for E2, 4-OHTAM, and the synthetic estrogen diethylstilbestrol (DES) were determined to be 0.051 ± 0.010 nM, 0.032 ± 0.010 nM, and 0.042 ± 0.011 nM, respectively. Interestingly, the competition binding curves (from 1 nM to 20 μM) (Figure 7) showed that lignans bound to rhERα [33] with IC_50_ values between 0.16 μM and 8 μM for compounds **14** and **9**, respectively (Table 3).

Finally, rat uterine cytosol (RUC) from ovariectomized rats was used to validate the binding of the most active compound **14** to native Erα by using a competitive radioligand binding assay [34]. Accordingly, compound **14** effectively competed with radio-labeled E2 by binding to ERα from RUC with an IC_50_ value of 3.96 ± 0.98 μM.

In general terms, the best results were obtained with lignan derivatives having two hydroxyl groups in the B ring, among which the dibenzylbutane-type lignans showed the best activities.

### 2.3. Molecular Docking and Induced Fit (IFD)

In order to explain the obtained biological activity data and propose a possible binding mode of the lignan derivatives, we carried out a molecular docking study on the antagonistic conformation of the crystal structure of hERα LBD in complex with active SERM 4-OHTAM. The ERα is an intracellular transcription factor that belongs to the large superfamily of nuclear receptors and is responsible for mediating most of the biological activity attributed to estrogens. The LBD of hERα, a well-defined domain, has allowed the pharmacological development of agonists and antagonists for the treatment of various disorders. This domain is located in the middle of the carboxy-terminal region of the receptor, the final portion of which is critical and it is responsible for ligand binding, receptor dimerization, and nuclear translocation [35].

The LBD consists of twelve α-helices (H-1 to H-12) and a beta sheet/hairpin. The amino acid residues that line the ligand-binding cavity or interact with bound ligand span from helix 3 (H-3) to helix 12 (H-12). When the receptor binds to the ligand a change in its three-dimensional structure is produced, the LBD forms a bag-shaped structure, hydrophobic in nature, that lodges the ligand. In ERα-LBD, only helix 12 (H12), on the C-terminus is highly dynamic and plays an important role as a molecular switch by serving as a gate to regulate the binding of coactivators [36,37]. Thus, H12 is an essential element in ERα function by adopting different key ligand-dependent conformations that allow the activation of the receptor. When it binds to an agonist, the LBD adopts an active conformation where H-12 rests across H-3 and H-11, and forms a groove to accommodate coregulator binding and facilitate the downstream activation process for gene transcription. When it binds to an antagonist, H-12 is displaced from this position and adopts a new conformation which results in the distortion of this coregulator binding groove and prevents receptor activation [38,39].

Protein-ligand docking is an effective method commonly used to predict the binding mode and the affinity of a ligand in a protein-binding site [40,41]. Therefore, we have docked all lignan derivatives into the hydrophobic binding pocket of LBD, to determine the likely binding pattern, key interactions within the active site as well as the binding free energy approximation of each of them.

According to the docking results, it was observed that the best docking poses for the most compounds share a common binding mode into hydrophobic binding pocket. However, only those compounds that have free phenolic hydroxyl groups in its structure are located towards the end of the pocket where the cognate ligand is usually found in the crystal structure of ERα forming two hydrogen bond interactions.

On the one hand, in the predicted pose of the most active compound **14**, this lignan with antagonistic activity is able to form two stable hydrogen bond interactions with residues Glu353 and Arg394, which are key interactions for biological activity, as well as displays multiple potential hydrophobic interactions involving residues such as Leu525, Leu349, Met343, Leu346, Asp351, Leu391, Thr347, Trp383, Leu384, and Leu387, which probably play a predominant roles in protein-ligand interactions. According to the predicted binding mode, the best docking scores were found in the range from −8.95 to −8.79 kcal·mol^−1^, suggesting that these interactions were so effective that they provided a stabilizing effect on the active antiestrogenic conformation of ER-LBD. On the other hand, compounds with methoxy groups on the aromatic rings form a bulky hindrance that prevents a proper interaction with the receptor, due to the presence of new additional hydrophobic interactions, less efficient than hydrogen bonds mentioned above.

Furthermore, it is widely accepted that protein flexibility should be incorporated into docking studies to more realistically model protein-ligand binding. In this sense, given the plasticity and flexibility of the ER-LBD, the binding modes of the all active compounds were regenerated through glide/induced fit docking (IFD) [42,43,44].

Interestingly, the IFD binding modes for the most active compound **14** showed an optimized network of protein ligand interactions as compared with previous docking results. The results revealed that both free phenolic hydroxyl groups were primarily oriented towards the Glu353, forming two hydrogen bonds with this residue, which was also stabilized by forming a new hydrogen bond between the oxygen of one of the bulky methoxy group located on the second aromatic ring with the Trp383 residue located in the hydrophobic channel. Additionally, these results showed a considerable increase in the GlideScore value −10.84 kcal·mol^−1^ for the most stable conformation. These findings suggested that the novel interaction observed by compound **14** as well as the new orientation of the ligand, provided a stabilizing effect on the active antiestrogenic conformation on ER-LBD, which could explain the high affinity value for the receptor binding site, as evidenced by the biological activity data.

**Figure 8:**
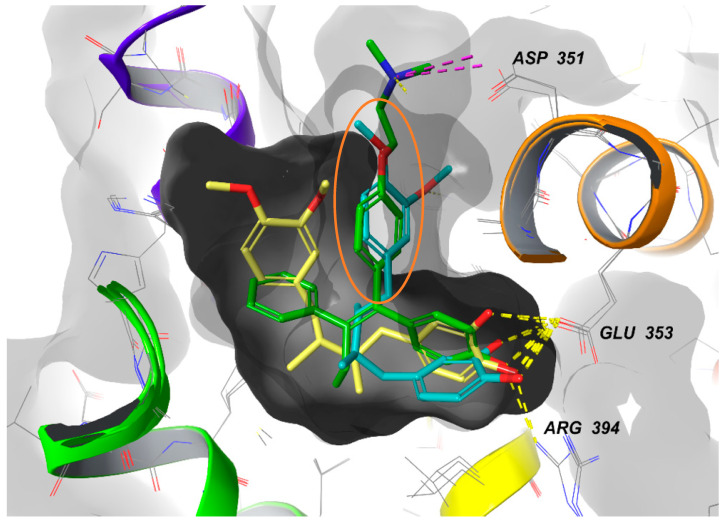
Predicted binding modes of compound **14** (yellow and turquoise) into the binding site of ERα. Superimposed on the crystal structure of 4-hydroxy-tamoxifen (green, PDB 3ERT). The orange circle indicates how the aromatic ring with two methoxyl groups occupies the region where the dimethylamino-ethoxy-phenyl group is found.

In addition, in an attempt to explain the antiestrogenic activity of the most active compound, an overlay of the crystal structure of ERα with 4-hydroxy-tamoxifen and the best conformations of compound **14** bound to the Erα-LBD was made. It revealed how this compound, through a conformational change, housed the aromatic ring with the two methoxyl groups in the same region where the dimethylamino-ethoxy-phenyl group of 4-hydroxy-tamoxifen is found (Figure 8).

Thus, we hypothesized that the antiestrogenic activity could result from an alternative binding mode which might correspond to the antagonists’ conformations that fitted into this cavity, forming hydrophobic interactions similar to those of the cognate ligand. The root-mean-square distance (RMSD) value obtained between this top-ranked docking solution and the crystal structure was 2.0 Å. The induced fit docking studies revealed that the most active compounds had appreciable binding free energies and affinities for the ERα.

### 2.4. Molecular Dynamic Simulations

To further assess the reliability of the obtained results and to confirm the stability of the system, we combined the docking protocols with molecular dynamics (MD) simulation techniques to study conformational changes in the ligand–receptor complex over simulation time [45,46]. Simulations provide every minute detail about every atom movement with respect to time and can help in answering questions arising on the deviation and fluctuation pattern of protein.

**Figure 9:**
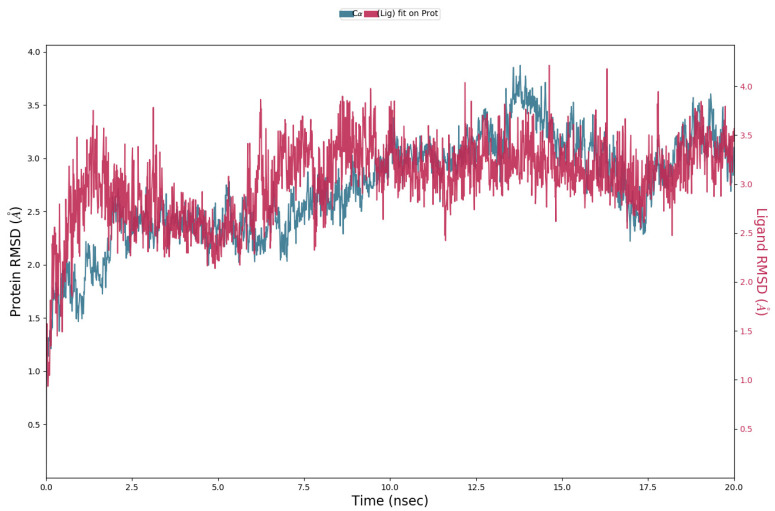
RMSDs of carbon atoms of the protein and compound **14** through MD simulation.

The MD simulation was performed on the best scoring complex of compound **14** obtained from the IFD for 20 ns in an explicit aqueous solution environment with periodic boundary conditions. The OPLS3e force field and the TIP3P solvent model employing the Desmond simulation package seamlessly integrated into the Maestro software were used. The root-mean-square deviation (RMSD) for the complex backbone atoms along the entire trajectory were measured to validate the dynamic stability of the complexes (Figure 9). We observed that after an initial period of disturbance, the RMSD curves of the protein backbone (Cα) and the ligand (Lig fit Prot) demonstrated that our simulation had equilibrated and converged into equilibrium. The RMSD of compound **14** in the binding pocket during the 20 ns simulation showed deviations between 0.96 and 4.00 Å. The ERα (PDB 3ERT) altered its RMSD from 1.5 Å in the starting frame to a maximum of 3.85 Å. Higher fluctuations were observed between 13 and 15 ns followed by a stable RMSD up to the final 20 ns. The convergence of RMSD values indicated that compound **14** and ERα maintained its interaction through the simulation.

**Figure 10:**
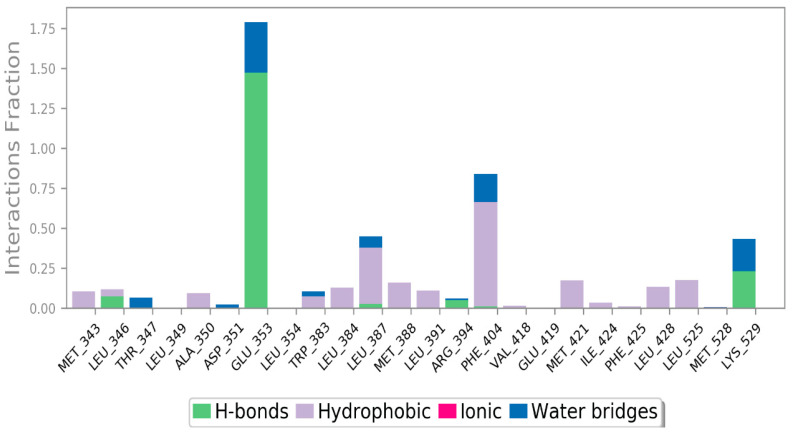
Stacked bar charts designating the contacts of ERα (3ERT) residues with ligands. These interactions are of four types: hydrogen bonds, hydrophobic, ionic, and water bridges. A value of 1.0 signifies that during 100% of the simulation time a particular interaction is maintained. Values above 1.0 indicate that a single amino acid residue forms more than one interaction with the ligand.

In addition, the analysis of the trajectory as well as the protein-ligand contact analysis reveals that the compound does not leave the binding cavity of the protein and remains in the same orientation during the entire simulation. It also efficiently interacts with the Glu353 residue forming the double hydrogen bond mentioned above which occurs more than 90.0% of the simulation time in the selected trajectory (Figure 10).

The graphical snapshot of the production phase of this compound also reveals the presence of a new hydrophobic π–π stacking interaction edge-to-face that had not been previously detected. This interaction occurs between the aromatic ring having the two free hydroxyl groups and a Phe404 residue, which could explain the great affinity of this derivative.

**Figure 11:**
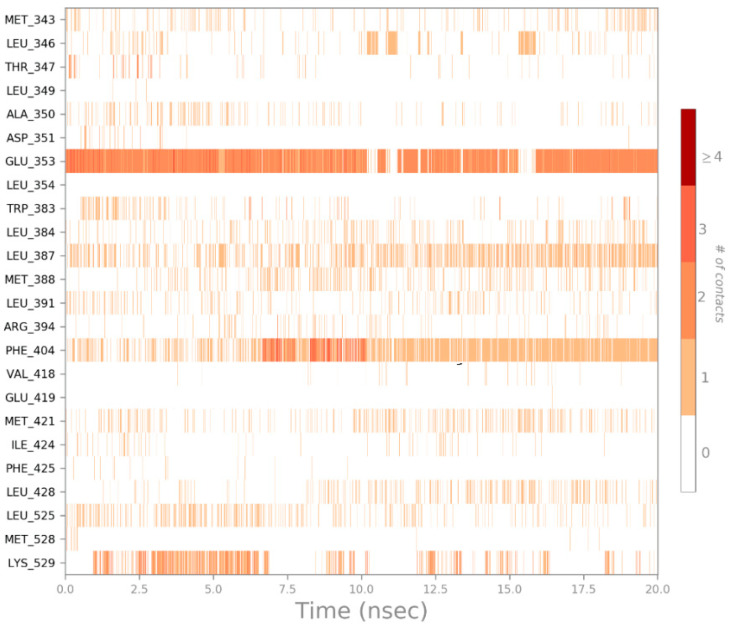
Interaction diagram for compound **14** and ERα ligand binding domain (LBD) obtained in molecular dynamic simulation study.

**Figure 12:**
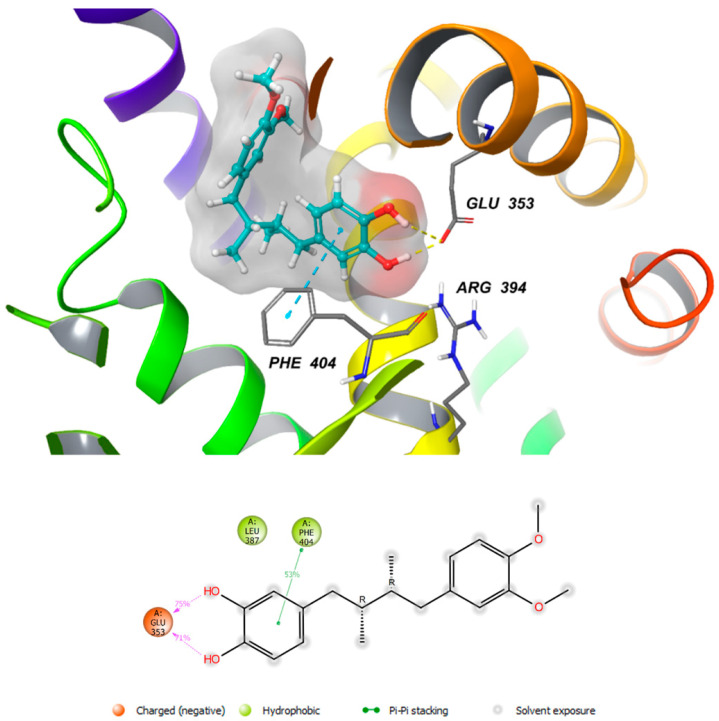
Interactions of compound **14** with the key amino acid residues at the binding pocket of ERα (IFD score −10.84 kcal·mol^−1^). The colors indicate residue type: green are lipophilic residues, orange is a charged polar residue, ligand atoms that are exposed to solvent are marked with grey spheres.

Interestingly, a flashing hydrogen bond interaction is observed between the oxygens of the methoxy groups with a Lys529 residue of the H12 that is not part of the hydrophobic pocket, but which could, perhaps, explain that the H12 remains displaced in its antagonistic conformation during the course of the molecular dynamics simulation. These interactions of the protein with the ligand are observed as interaction fraction throughout the molecular dynamics simulation (Figure 11 and Figure 12). The stacked bar charts are normalized over the course of the trajectory, for example, a value of 0.7 suggests that 70% of the simulation time the specific interaction is maintained. This demonstrates the role of each of the particular bonds with the amino acid residues responsible for the stabilization of the complex. Values over 1.0 are possible as some protein residue may make multiple contacts of the same subtype with the ligand. This model and subsequent dynamical observations can help to explain the activity of compound **14,** confirming the affinity for the ERα.

### 2.5. In Silico ADME Predictions

To further explore the antiestrogenic potential of our most active compounds, predictions of pharmacokinetic properties such as absorption, distribution, metabolism, and excretion (ADME) were made in order to understand their pharmacokinetic profiles. It is worth mentioning that in many cases, unfavorable pharmacokinetic profiles are responsible for the failure of many drug candidates, therefore, it is important to incorporate the prediction of these types of properties in the selection of new drug candidates. Properties such as drug-likeness, permeability, solubility, bioavailability, and oral absorption provide insights into key aspects for the new drug development [47,48]. Ideally, the drug should be readily absorbed from the site of administration, transported to the active site without non-specific interactions, and have maximum efficacy and safety, as well as metabolize without producing toxic metabolites, and then be able to be easily eliminated. ADMET properties of the compounds **1**, **3**, **4**, **7**, **9**, **11**, **13**, and **14** were calculated using the Qikprop program (Qikprop, version 6.3, Schrödinger, LLC, New York, NY, 2020), which provided predicted values for physically and pharmaceutically relevant parameters and its recommended range of values (Table 4).

As a first test of the drug-likeness of the ligands, we applied Lipinski’s rule of 5. As can be seen, the partition coefficient (QPlogPo/w), the hydrogen bond donors (HB donor), the hydrogen bond acceptors (HB acceptor), the molecular weight (mol. wt.), and the human oral absorption percentage exhibited satisfactory results and also most of them showed values according to the Lipinski′s rules. The aqueous solubility of a drug candidate is also a crucial property for its bioavailability, all ligands, except for compound **13,** are satisfactory with respect to their QPlogS values. As can be seen, the high absorption and permeability of the compounds were confirmed by the non-violation of any of the Lipinski’s rules, and also by the high values for parameters concerning cell permeability as blood–brain barrier mimic MDCK cell permeability (QPPMDCK) and by the predicted Caco-2 cells permeability (QPPCaco) used as a model for the gut–blood barrier. QPlogKhsa is the prediction of binding to human serum albumin and all compounds lie within the expected range for 95% of known drugs. The QPlogBB (brain/blood) barrier coefficient is satisfactory for the most active compounds. Therefore, the above data indicate that compounds **1**, **3**, **4**, **7**, **9**, **11**, **13**, and **14** exhibit very good drug-likeness, as well as meet all the pharmacokinetic criteria. Therefore, they can be considered to be candidate leads.

## 3. Materials and Methods

### 3.1. General Experimental Procedures

The optical rotations were measured on a Perkin Elmer (Shelton, CT, USA) 241 polarimeter. The IR spectra were obtained using a Bruker IFS28/55 spectrophotometer. The NMR spectra were recorded in CDCl_3_ or MeOD-*d*_4_ at 500 or 600 MHz for ^1^H NMR and 125 or 150 MHz for ^13^C NMR. Chemical shifts (δ) are given in parts per million and coupling constants (*J*) in hertz (Hz). The ^1^H and ^13^C NMR spectra were referenced using the solvent signal as internal standard. The HREIMS were recorded using a high-resolution magnetic trisector (EBE) mass analyzer. The analytical TLC plates used were Polygram-Sil G/UV254. The preparative TLC was carried out with Analtech silica gel GF plates (20 × 20 cm, 1000 μm) using appropriate mixtures of ethyl acetate and hexanes. All solvents and reagents were purified by standard techniques reported [49] or used as supplied from commercial sources. The (−)-bursehernin and (−)-matairesinol dimethyl ether used as starting materials were isolated from *Bupleurum salicifolium*, following the procedure described in reference [22].

### 3.2. (−)-Bursehernin *(**1**)*

^1^H-NMR (CDCl_3_, 500 MHz) *δ* 6.76 (1H, d, *J* = 8.1 Hz, H-5), 6.62–6.67 (3H, m, H-2, H-5′, H-6), 6.42 (1H, d, *J* = 8.1 Hz, H-6′), 6.40 (1H, s, H-2′), 5.88 (2H, d, *J* = 3.7 Hz, -OCH_2_O-), 4.08 (1H, dd, *J* = 7.3, 7.0 Hz, H-9′b), 3.86 (4H, brs, CH_3_, H-9′a), 3.84 (3H, s, CH_3_) 2.93 (1H, dd, *J* = 14.0, 5.1 Hz, H-7b), 2.84 (1H, dd, *J* = 14.0, 7.0 Hz, H-7a), 2.49–2.58 (2H, m, H-7b′, H-8), 2.39–2.48 (2H, m, H-7′a, H-8′); ^13^C-NMR (CDCl_3_, 125 MHz) *δ* 178.5 (C, C-9), 148.9 (C, C-3), 147.8 (C, C-3′), 147.7 (C, C-4), 146.2 (C, C-4′), 131.6 (C, C-1′), 130.1 (C, C-1), 121.4 (CH, C-6′), 121.2 (CH, C-6), 112.2 (CH, C-2), 111.1 (CH, C-5), 108.6 (CH, C-2′), 108.1 (CH, C-5′), 100.9 (CH_2_), 71.0 (CH_2_, C-9′), 55.7 (CH_3_), 55.6 (CH_3_), 46.3 (CH, C-8), 40.9 (CH, C-8′), 38.1 (CH_2_, C-7′), 34.5 (CH_2_, C-7).EIMS *m*/*z* 370 ([M]^+^, 94) 234 (51), 208 (50), 185 (43), 161 (42), 152 (71), 151 (100), 136 (60), 135 (83), 77 (66); HREIMS *m*/*z* 370.1432 [M]^+^ (calcd for C_21_H_22_O_6_, 370.1416).

### 3.3. (−)-Matairesinol Dimethyl Ether *(**2**)*

^1^H-NMR (CDCl_3_, 500 MHz) *δ* 6.71–6.75 (2H, m, H-5, H-5′), 6.65 (1H, s, H-2), 6.63 (1H, d, *J* = 8.6 Hz, H-6), 6.52 (1H, d, *J* = 8.6 Hz, H-6′), 6.46 (1H, s, H-2′), 4.09 (1H, dd, *J* = 7.2, 7.1 Hz, H-9′b), 3.85 (1H, d, *J* = 8.2 Hz, H-9′a), 3.83 (3H, s, CH_3_), 3.82 (3H, s, CH_3_), 3.80 (3H, s, CH_3_), 3.79 (3H, s, CH_3_), 2.94 (1H, dd, *J* = 14.1, 5.5 Hz, H-7b), 2.88 (1H, dd, *J* = 14.1, 6.6 Hz, H-7a), 2.53–2.64 (2H, m, H-7′b, H-8), 2.42–2.52 (2H, m, H-7′a, H-8′); ^13^C-NMR (CDCl_3_, 125 MHz) *δ* 178.1 (C, C-9), 149.1 (2xC, C-3, C-3′), 147.9 (C, C-4/C-4′), 147.8 (C, C-4/C-4′), 130.5 (C, C-1), 130.2 (C, C-1′), 121.4 (CH, C-6), 120.6 (CH, C-6′), 112.4 (CH, C-2), 111.9 (CH, C-2′), 111.4 (CH, C-5/C-5′), 111.1 (CH, C-5/C-5′), 71.2 (CH_2_, C-9′), 55.9 (CH_3_), 55.8 (3 × CH_3_), 46.6 (CH, C-8), 41.1 (CH, C-8′), 38.2 (CH_2_, C-7′), 34.5 (CH_2_, C-7); EIMS *m*/*z* 386 ([M]^+^, 97), 193 (49), 177 (61), 151 (100), 121 (48), 107 (64), 106 (57), 91 (53), 79 (51), 78 (51), 77 (51); HREIMS *m*/*z* 386.1746 [M]^+^ (calcd for C_22_H_26_O_6_, 386.1729).

### 3.4. General Procedure for the Demethylation

To a solution of the corresponding methoxylated lignan in 5 mL of dry DCM, under argon atmosphere at 0 °C, BBr_3_ (1 M solution in DCM, 1.5–5.0 equiv) was added dropwise. Upon complete addition of BBr_3_, the reaction mixture was stirred at 0 °C for 1 h. After this time, it was allowed to warm to room temperature for 18 h. Then, the mixture was cooled to 0 °C and carefully quenched with H_2_O (15 mL). It was repeatedly extracted with EtOAc (3 × 15 mL), and the organic layers dried over anhydrous Mg_2_SO_4_. After solvent removal, the product was purified by silica gel preparative TLC using 40–50% Hex/EtOAc as eluent to afford the desired product.

### 3.5. General Procedure for Methylenedioxy Deprotection

To a stirred suspension of anhydrous AlCl_3_ (5 equiv) in 3 mL of dry DCM at 0 °C under argon atmosphere, 20 mg of the corresponding lignan in 2 mL of dry DCM was added. The reaction mixture was maintained at 0 °C for 1 h, and then stirred at room temperature for 18 h. The mixture was cooled to 0 °C and carefully quenched with H_2_O (15 mL). Then, it was repeatedly extracted with EtOAc (3 × 15 mL) and the organic layers were collected and dried over anhydrous Mg_2_SO_4_. Upon solvent removal, the residue was purified by silica gel preparative TLC using 30–60% Hex/EtOAc as eluent to afford the desired products.

### 3.6. (3R,4R)-3,4-Bis(3,4-dihydroxybenzyl)dihydrofuran-2(3H)-one *(**3**)*

Following the general demethylation procedure, 25.9 mg (0.072 mmol) of (−)-bursehernin (**1**) in 5 mL of DCM were treated with 216 µL (0.216 mmol, 3 equiv) of a 1 M BBr_3_ solution in DCM. The crude was purified by preparative TLC using Hex/EtOAc (3:2) to provide 23.7 mg (100%) of compound **3** as colorless oil. [α]^20^_D_ −27.9 (*c* 0.003, CHCl_3_); ^1^H-NMR (MeOD-*d*_4_, 500 MHz) *δ* 6.70 (1H, d, *J* = 8.0 Hz, H-5′), 6.64–6.67 (2H, m, H-2, H-5), 6.51 (1H, d, *J* = 2.0 Hz, H-2′), 6.49 (1H, dd, *J* = 8.1, 2.0 Hz, H-6), 6.39 (1H, dd, *J* = 8.1, 2.0 Hz, H-6′), 4.02 (1H, dd, *J* = 9.0, 7.6 Hz, H-9′b), 3.84 (1H, t, *J* = 8.5 Hz, H-9′a), 2.83 (1H, dd, *J* = 14.1, 5.4 Hz, H-7b), 2.77 (1H, dd, *J* = 14.0, 6.6 Hz, H-7a), 2.55–2.60 (1H, m, H-8), 2.42–2.52 (2H, m, H-7′b, H-8′), 2.31–2.37 (1H, m, H-7′a); ^13^C-NMR (MeOD-*d*_4_, 125 MHz) *δ* 181.6 (C, C-9), 146.3 (C, C-3/C-3′), 146.2 (C, C-3/C-3′), 145.1 (C, C-4), 144.8 (C, C-4′), 131.5 (C, C-1), 130.8 (C, C-1′), 121.9 (CH, C-6), 121.1 (CH, C-6′), 117.4 (CH, C-2), 116.8 (CH, C-2′), 116.4 (CH, C-5′), 116.3 (CH, C-5), 72.8 (CH_2_, C-9′), 47.7 (CH, C-8), 42.5 (CH, C-8′), 38.5 (CH_2_, C-7′), 35.1 (CH_2_, C-7); EIMS *m*/*z* 330 ([M]^+^, 33), 151 (19), 125 (15), 124 (57), 123 (100), 77 (15), 55 (14); HREIMS *m*/*z* 330.1123 [M]^+^ (calcd for C_18_H_18_O_6_, 330.1103).

### 3.7. (3R,4R)-4-(3,4-Dihydroxybenzyl)-3-(3,4-dimethoxybenzyl)dihydrofuran-2(3H)-one *(**4**)*

Following the general procedure for removing the methylenedioxy, 17.1 mg of (−)-bursehernin (**1**) (0.047 mmol) were treated with 32.3 mg of anhydrous AlCl_3_ (0.239 mmol). The crude was purified by preparative TLC using Hex/EtOAc (1:1) to provide 8.92 mg (53%) of compound **4** as colorless oil. [α]^20^_D_ −18.5 (*c* 0.003, CHCl_3_); ^1^H-NMR (CDCl_3_, 500 MHz) *δ* 6.78 (1H, d, *J* = 8.3 Hz, H-5), 6.72 (1H, d, *J* = 8.2 Hz, H-5′), 6.68 (1H, dd, *J* = 8.2, 1.8 Hz, H-6), 6.64 (1H, d, *J* = 1.8 Hz, H-2), 6.46 (1H, d, *J* = 1.8 Hz, H-2′), 6.41 (1H, dd, *J* = 8.1, 1.8 Hz, H-6′), 5.58 (1H, brs, OH), 5.41 (1H, brs, OH), 4.12 (1H, dd, *J* = 9.2, 7.0 Hz, H-9′b), 3.86 (3H, s, CH_3_), 3.80–3.84 (1H, m, H-9′a), 3.82 (3H, s, CH_3_), 2.96 (1H, dd, *J* = 14.0, 5.0 Hz, H-7b), 2.87 (1H, dd, *J* = 14.0, 7.0 Hz, H-7a), 2.51–2.59 (2H, m, H-7′b, H-8), 2.42–2.50 (2H, m, H-7′a, H-8′); ^13^C-NMR (CDCl_3_, 125 MHz) *δ* 179.5 (C, C-9), 149.1 (C, C-3), 148.0 (C, C-4), 144.2 (C, C-3′), 142.7 (C, C-4′), 130.7 (C, C-1′), 130.3 (C, C-1), 121.7 (CH, C-6), 120.9 (CH, C-6′), 115.8 (CH, C-2′), 115.6 (CH, C-5), 112.6 (CH, C-2), 111.5 (CH, C-5), 71.6 (CH_2_, C-9′), 56.0 (2 × CH_3_), 46.7 (CH, C-8), 41.0 (CH, C-8′), 37.9 (CH_2_, C-7′), 34.7 (CH_2_, C-7); EIMS *m*/*z* 358 ([M]^+^, 90), 208 (41), 152 (68), 151 (100), 135 (48), 131 (45), 123 (60), 77 (46); HREIMS *m*/*z* 358.1426 [M]^+^ (calcd for C_20_H_22_O_6_, 358.1416).

### 3.8. (3R,4R)-4-(Benzo[d][1,3]dioxol-5-ylmethyl)-3-(3,4-dimethoxybenzyl) Tetrahydro Furan-2-ol *(**5**)*

To a solution of (−)-bursehernin (**1**) (42 mg, 0.113 mmol) in anhydrous toluene (3 mL), a solution of diisobutylaluminum hydride (1.0 M in hexane, 0.34 mL, 0.340 mmol) was added over a period of 5 min at −78 °C. After 2 h, the reaction mixture was warmed to room temperature and treated with 10 mL of a saturated aqueous NH_4_Cl solution. The reaction mixture was extracted with EtOAc (3 × 10 mL) and the combined organic layers were dried over anhydrous MgSO_4_, filtered, and concentrated under reduced pressure. The residue was purified by silica gel preparative TLC using Hex/EtOAc (1:1) as eluent to afford 23.1 mg (55%) of lignan **5** as an inseparable mixture of epimers (1:1.6), as a pale-yellow oil. ^1^H-NMR (CDCl_3_, 500 MHz) *δ* 6.76 (1H, m, H-5, *major epimer*), 6.76 (3H, m, H-2, H-5, H-6, *minor epimer*), 6.71 (1H, d, *J* = 7.8 Hz, H-5′, *minor epimer*), 6.65 (2H, d, *J* = 7.8 Hz, H-5′, H-6, *major epimer*), 6.61 (1H, s, H-2′, *minor epimer*), 6.58 (1H, s, H-2, *major epimer*), 6.57 (1H, d, *J* = 7.8 Hz, H-6′, *minor epimer*), 6.49 (1H, d, *J* = 7.8 Hz, H-6′, *major epimer*), 6.47 (1H, s, H-2′, *major epimer*), 5.89–5.92 (2H, d, *J* = 4.6 Hz, -OCH_2_O-), 5.23 (1H, s, H-9), 4.07–4.13 (1H, m, H-9′b, *minor epimer*), 3.99 (1H, t, *J* = 7.8 Hz, H-9′b, *major epimer*), 3.86 (3H, s, CH_3_, *minor epimer*), 3.85 (3H, s, CH_3_, *minor epimer*), 3.85 (3H, s, CH_3_, *major epimer*), 3.82 (3H, s, CH_3_, *major epimer*), 3.78 (1H, t, *J* = 7.8 Hz, H-9′a, *major epimer*), 3.57 (1H, t, *J* = 7.8 Hz, H-9′a, *minor epimer*), 3.05 (1H, brs, OH), 2.71–2.81 (2H, m, H-7′b, H-7b, *minor epimer*), 2.65–2.71 (1H, m, H-7b, *major epimer*), 2.59–2.64 (1H, m, H-7a, *minor epimer*), 2.55–2.58 (2H, m, H-7′b, H-7′a, *major epimer*), 2.41–2.48 (1H, m, H-7a, *major epimer*), 2.41–2.48 (2H, m, H-7′a, H-8′a, *minor epimer*), 2.12–2.19 (2H, m, H-8, H-8′, *major epimer*), 2.00–2.06 (1H, m, H-8, *minor epimer*); ^13^C-NMR (CDCl_3_, 125 MHz) *δ* 148.9 (C, C-3′, *major epimer*), 148.8 (C, C-3′, *minor epimer*), 147.8 (C, C-3, *minor epimer*), 147.7 (C, C-3, *major epimer*), 147.5 (C, C-4′, *major epimer*), 147.4 (C, C-4′, *minor epimer*), 146.0 (C, C-4, *minor epimer*), 145.9 (C, C-4, *major epimer*), 134.2 (C, C-1′, *major epimer*), 134.0 (C, C-1′, *minor epimer*), 133.4 (C, C-1, *minor epimer*), 133.2 (C, C-1, *major epimer*), 121.5 (CH, C-6′), 120.9 (CH, C-6, *major epimer*), 120.8 (CH, C-6, *minor epimer*), 112.2 (CH, C-2, *minor epimer*), 112.0 (CH, C-2, *major epimer*), 111.2 (CH, C-5, *minor epimer*), 111.1 (CH, C-5, *major epimer*), 109.0 (CH, C-2′, *minor epimer*), 108.9 (CH, C-2′, *major epimer*), 108.3 (CH, C-5′, *minor epimer*), 108.1 (CH, C-5′, *major epimer*), 103.5 (CH, C-9, *major epimer*), 101.0 (CH_2_, *minor epimer*), 100.9 (CH_2_, *major epimer*), 99.0 (CH, C-9, *minor epimer*), 72.7 (CH_2_, C-9′, *minor epimer*), 72.3 (CH_2_, C-9′, *major epimer*), 56.0 (CH_3_, *minor epimer*), 55.9 (CH_3_), 55.8 (CH_3_, *major epimer*), 53.1 (CH, C-8, *major epimer*), 51.9 (CH, C-8, *minor epimer*), 46.0 (CH, C-8′, *major epimer*), 43.0 (CH, C-8′, *minor epimer*), 39.3 (CH_2_, C-7′, *major epimer*), 38.9 (CH_2_, C-7′, *minor epimer*), 38.4 (CH_2_, C-7, *major epimer*), 33.5 (CH_2_, C-7, *minor epimer*); EIMS *m*/*z* 372 ([M]^+^, 42), 354 (29), 219 (50), 218 (29), 152 (35), 151 (100), 135 (46), 81 (76); HREIMS *m*/*z* 372.1577 [M]^+^ (calcd for C_21_H_24_O_6_, 372.1573).

### 3.9. (3R,4R)-3,4-Bis(3,4-dimethoxybenzyl)tetrahydrofuran-2-ol *(**6**)*

To a solution of (−)-matairesinol dimethyl ether (**2**) (42 mg, 0.108 mmol) in anhydrous toluene (3 mL), a solution of diisobutylaluminum hydride (1.0 M in hexane, 0.33 mL, 0.326 mmol) was added over a period of 5 min at −78 °C. After 2 h at −78 °C, the reaction mixture was warmed to room temperature and treated with 10 mL of a saturated aqueous NH_4_Cl solution. Then, it was extracted with EtOAc (3 × 10 mL) and the combined organic layer was dried with MgSO_4_, filtered, and concentrated under reduced pressure. The residue was purified by silica gel preparative TLC using Hex/EtOAc (3:2) as eluent to afford lignan **6** as an inseparable mixture of epimers (1:1.8) as a pale-yellow oil (44.4 mg, 0.108 mmol, 100% yield). ^1^H-NMR (CDCl_3_, 500 MHz) *δ* 6.76–6.79 (1H, m, H-5/H-5′, *major epimer*), 6.76–6.79 (3H, m, H-2, H-5/H-5′, H-6, *minor epimer*), 6.74 (1H, d, *J* = 3.7 Hz, H-5/H-5′, *major epimer*), 6.72 (1H, s, H-5/H-5′, *minor epimer*), 6.68 (1H, dd, *J* = 8.1, 1.8 Hz, H-6′, *minor epimer*), 6.63–6.66 (1H, m, H-6, *major epimer*), 6.63–6.66 (1H, m, H-2′, *minor epimer*), 6.61 (1H, d, *J* = 1.8 Hz, H-2, *major epimer*), 6.59 (1H, dd, *J* = 8.1, 1.8 Hz, H-6′, *major epimer*), 6.54 (1H, d, *J* = 1.8 Hz, H-2′, *major epimer*), 5.24 (1H, brs, H-9), 4.10 (1H, t, *J* = 8.1 Hz, H-9′b, *minor epimer*), 4.00 (1H, dd, *J* = 8.7, 6.7 Hz, H-9′b, *major epimer*), 3.81–3.87 (13H, m, H-9′a, 4 × CH_3_, *major epimer*), 3.81–3.87 (12H, m, 4 × CH_3_, *minor epimer*), 3.60 (1H, t, *J* = 8.0 Hz, H-9′a, *minor epimer*), 2.77–2.83 (2H, m, H-7b, H-7′b, *minor epimer*), 2.58–2.71 (3H, m, H-7b, H-7′a, H-7′b, *major epimer*), 2.58–2.71 (2H, m, H-7a, H-7′a, *minor epimer*), 2.42–2.51 (1H, m, H-7a, *major epimer*), 2.42–2.51 (1H, m, H-8′, *minor epimer*), 2.16–2.22 (2H, m, H-8, H-8′, *major epimer*), 2.03–2.09 (1H, m, H-8, *minor epimer*); ^13^C-NMR (CDCl_3_, 125 MHz) *δ* 149.00 (C, C-3/C-3′, *minor epimer*), 148.96 (C, C-3/C-3′, *major epimer*), 148.91 (C, C-3/C-3′, *minor epimer*), 148.89 (C, C-3/C-3′, *major epimer*), 147.6 (C, C-4/C-4′, *minor epimer*), 147.5 (C, C-4/C-4′, *major epimer*), 147.4 (C, C-4/C-4′), 133.4 (C, C-1′, *minor epimer*), 133.1 (C, C-1′, *major epimer*), 132.8 (C, C-1, *minor epimer*), 132.3 (C, C-1, *major epimer*), 120.9 (CH, C-6, *major epimer*), 120.8 (CH, C-6, *minor epimer*), 120.6 (CH, C-6′, *minor epimer*), 120.5 (CH, C-6′, *major epimer*), 112.3 (CH, C-2, *minor epimer*), 112.2 (CH, C-2, *major epimer*), 111.9 (CH, C-2′, *major epimer*), 111.8 (CH, C-2′, *minor epimer*), 111.29 (CH, C-5/C-5′, *minor epimer*), 111.28 (CH, C-5/C-5′, *minor epimer*), 111.22 (CH, C-5/C-5′, *major epimer*), 111.17 (CH, C-5/C-5′, *major epimer*), 103.5 (CH, C-9, *major epimer*), 99.1 (CH, C-9, *minor epimer*), 72.9 (CH_2_, C-9′, *minor epimer*), 72.5 (CH_2_, C-9′, *major epimer*), 56.01 (2 × CH_3_, *minor epimer*), 55.98 (2 × CH_3_, *major epimer*), 55.97 (2 × CH_3_, *major epimer*), 55.88 (2 × CH_3_, *minor epimer*), 53.3 (CH, C-8, *major epimer*), 52.1 (CH, C-8, *minor epimer*), 46.2 (CH, C-8′, *major epimer*), 42.9 (CH, C-8′, *minor epimer*), 39.2 (CH_2_, C-7′, *major epimer*), 38.9 (CH_2_, C-7′, *minor epimer*), 38.5 (CH_2_, C-7, *major epimer*), 33.6 (CH_2_, C-7, *minor epimer*); HRESIMS *m*/*z* 411.1788 [M]^−^ (calcd for C_22_H_28_O_6_Na, 411.1784).

### 3.10. (2R,3R)-2-(Benzo[d][1,3]dioxol-5-ylmethyl)-3-(3,4-dimethoxybenzyl)butane-1,4-diol *(**7**)*

A solution of 51.4 mg (0.139 mmol) of (−)-bursehernin (**1**) in 3 mL of dry THF was added dropwise to a stirring suspension of 53.0 mg (1.39 mmol) of LiAlH_4_ in dry THF under argon atmosphere. The mixture was stirred for 1 h, and then quenched with 5 mL of a saturated aqueous NH_4_Cl solution and acidified with 1 M HCl. The solution was extracted with EtOAc (3 × 15 mL) and the organic layers were dried over anhydrous Mg_2_SO_4_. The solvent was removed under reduced pressure and the residue was purified by silica gel preparative TLC using Hex/EtOAc (3:2) as eluent to afford 51.6 mg (100%) of compound **7** as a colorless oil. [α]^20^_D_ −19.2 (*c* 0.003, CHCl_3_); ^1^H-NMR (CDCl_3_, 500 MHz) *δ* 6.75 (1H, d, *J* = 8.0 Hz, H-5), 6.60–6.70 (2H, m, H-5′, H-6), 6.64 (1H, d, *J* = 1.9 Hz, H-2), 6.61 (1H, d, *J* = 1.6 Hz, H-2′), 6.58 (1H, dd, *J* = 7.9, 1.7 Hz, H-6′), 5.89 (2H, s, -OCH_2_O-), 3.83 (3H, s, CH_3_), 3.81 (3H, s, CH_3_), 3.77 (2H, dt, *J* = 11.4, 2.7 Hz, H-9b/H-9′b), 3.49 (2H, dt, *J* = 11.4, 4.8 Hz, H-9a/H-9′a), 2.69–2.78 (2H, m, H-7b/H-7′b), 2.58–2.67 (2H, m, H-7a/H-7′a), 1.80–1.90 (2H, m, H-8/H-8′); ^13^C-NMR (CDCl_3_, 125 MHz) *δ* 148.9 (C, C-3), 147.6 (C, C-3′), 147.4 (C, C-4), 145.8 (C, C-4′), 134.5 (C, C-1′), 133.2 (C, C-1), 121.9 (CH, C-6′), 121.1 (CH, C-6), 112.2 (CH, C-2), 111.3 (CH, C-5), 109.4 (CH, C-2′), 108.2 (CH, C-5′), 100.8 (CH_2_), 60.4 (CH_2_, C-9/C-9′), 60.3 (CH_2_, C-9/C-9′), 56.0 (CH_3_), 55.9 (CH_3_), 44.2 (CH, C-8/C-8′), 44.0 (CH, C-8/C-8′), 36.0 (CH_2_, C-7/C-7′), 35.8 (CH_2_, C-7/C-7′); EIMS *m*/*z* 356 ([M-H_2_O]^+^, 73), 152 (78), 151 (100), 136 (52), 135 (70), 95 (44), 71 (70), 57 (81), 55 (60); HREIMS *m*/*z* 356.1635 ([M-H_2_O]^+^ (calcd for C_21_H_24_O_5_, 356.1624).

### 3.11. (2R,3R)-2,3-Bis(3,4-dimethoxybenzyl)butane-1,4-diol *(**8**)*

A solution of 30 mg (0.078 mmol) of (−)-matairesinol dimethyl ether (**2**) in 3 mL of dry THF was added dropwise to a stirring suspension of 26.6 mg (0.780 mmol) of LiAlH_4_ in dry THF under argon atmosphere. The mixture was stirred for 1 h, and then quenched with 5 mL of a saturated aqueous NH_4_Cl solution and acidified with 1M aqueous HCl solution. The solution was extracted with EtOAc (3 × 15 mL) and the organic layers dried over anhydrous Mg_2_SO_4_. The solvent was removed and the residue was purified by silica gel preparative TLC with Hex/EtOAc (3:2) as eluent to afford 30.4 mg (100%) of compound **8** as a colorless oil. [α]^20^_D_–27.7 (*c* 0.003, CHCl_3_); ^1^H-NMR (CDCl_3_, 500 MHz) *δ* 6.75 (2H, d, *J* = 8.0 Hz, H-5/H-5′), 6.67 (2H, d, *J* = 8.0 Hz, H-6/H-6′), 6.64 (2H, s, H-2/H-2′), 3.83 (6H, s, 2 × CH_3_), 3.81 (6H, s, 2 × CH_3_), 3.79–3.82 (2H, m, H-9b/H-9′b), 3.52 (2H, dd, *J* = 11.0, 4.0 Hz, H-9a/H-9′a), 2.36 (2H, brs, 2 × OH), 2.76 (2H, dd, *J* = 13.9, 8.2 Hz, H-7b/H-7′b), 2.66 (2H, dd, *J* = 13.8, 6.3 Hz, H-7a/H-7′a), 1.87 (2H, s, H-8/H-8′); ^13^C-NMR (CDCl_3_, 125 MHz) *δ* 148.9 (C, C-3, C-3′), 147.4 (C, C-4, C-4′), 133.2 (C, C-1, C-1′), 121.1 (CH, C-6, C-6′), 112.3 (CH, C-2, C-2′), 111.3 (CH, C-5, C-5′), 60.6 (CH_2_, C-9, C-9′), 56.0 (2 × CH_3_), 55.9 (2 × CH_3_), 44.0 (CH, C-8, C-8′), 35.9 (CH_2_, C-7, C-7′); EIMS *m*/*z* 390 ([M]^+^, 73), 372 (23), 177 (19), 152 (72), 151 (100), 137 (19), 121 (17), 107 (25), 91 (20); HREIMS *m*/*z* 390.2044 [M]^+^ (calcd for C_22_H_30_O_6_, 390.2042).

### 3.12. 5-((3R,4R)-4-(3,4-Dimethoxybenzyl)tetrahydrofuran-3-yl)methyl)benzo [d][1,3] Dioxole *(**9**)*

To a solution of 89.9 mg (0.240 mmol) of diol **7** and 136 µL of pyridine (1.68 mmol) in 3 mL of DCM at 0 °C, 55.5 mg (0.288 mmol) of TsCl was added. The reaction mixture was stirred at room temperature for 24 h, then 10 mL of H_2_O and 10 mL of DCM were added. The organic layer was separated, washed with 10 mL of a 2.0 M aqueous HCl solution and 10 mL of a saturated aqueous NaHCO_3_ solution, and dried over anhydrous Mg_2_SO_4_. The solvent was removed and the residue was purified by silica gel preparative TLC with Hex/AcOEt (1:1) to afford 26.5 mg (31%) of compound **9** as a colorless oil. [α]^20^_D_ −19.6 (*c* 0.005, CHCl_3_); ^1^H-NMR (CDCl_3_, 500 MHz) *δ* 6.76 (1H, d, *J* = 7.8 Hz, H-5), 6.69 (1H, d, *J* = 8.1 Hz, H-5′), 6.63 (1H, d, *J* = 8.1 Hz, H-6), 6.58–6.51 (3H, m, H-2, H-2′, H-6′), 5.92 (2H, s, -OCH_2_O-), 3.93–3.87 (2H, m, H-9b, H-9′b), 3.85 (3H, s, -OCH_3_), 3.84 (3H, s, -OCH_3_), 3.55–3.47 (2H, m, H-9a, H-9′a), 2.63–2.55 (2H, m, H-7b, H-7′b), 2.54–2.47 (2H, m, H-7a, H-7′a), 2.21–2.12 (2H, m, H-8, H-8′); ^13^C-NMR (CDCl_3_, 125 MHz) *δ* 149.0 (C, C-3), 147.7 (C, C-3′), 147.5 (C, C-4), 146.0 (C, C-4′), 134.3 (C, C-1′), 133.1 (C, C-1), 121.6 (CH, C-6′), 120.7 (CH, C-6), 111.9 (CH, C-2), 111.3 (CH, C-5), 109.1 (CH, C-2′), 108.2 (CH, C-5′), 101.0 (CH_2_), 73.5 (CH_2_, C-9/C-9′), 73.4 (CH_2_, C-9/C-9′), 56.0 (CH_3_), 55.9 (CH_3_), 46.7 (CH, C-8/C-8′), 46.6 (CH, C-8/C-8′), 39.3 (CH_2_, C-7/C-7′), 39.2 (CH_2_, C-7/C-7′); EIMS *m*/*z* 356 ([M]^+^, 100), 152 (94), 151 (98), 136 (81), 135 (81), 121 (40), 77 (37); HREIMS *m*/*z* 356.1615 [M]^+^ (calcd for C_21_H_24_O_5_, 356.1624).

### 3.13. (3R,4R)-3,4-Bis(3,4-dimethoxybenzyl)tetrahydrofuran *(**10**)*

To a solution of 14.6 mg (0.037 mmol) of diol **8** and 21.7 µL (0.262 mmol) of pyridine in 3 mL of DCM at 0 °C, 8.3 mg (0.044 mmol) of TsCl was added. After the reaction mixture was stirred at room temperature for 24 h, 10 mL of H_2_O and 10 mL of DCM were added. The organic layer was separated, washed with 10 mL of a 2.0 M aqueous HCl solution and 10 mL of a saturated aqueous NaHCO_3_ solution, and dried over anhydrous Mg_2_SO_4_. The solvent was removed and the residue was purified by silica gel preparative TLC using Hex/EtOAc (3:2) as eluent to afford 2.47 mg (18%) of compound **10** as a colorless oil. [α]^20^_D_ −9.88 (*c* 0.002, CHCl_3_); ^1^H-NMR (CDCl_3_, 500 MHz) *δ* 6.75 (2H, d, *J* = 8.2 Hz, H-5, H-5′), 6.63 (2H, dd, *J* = 8.0, 2.1 Hz, H-6, H-6′), 6.58 (2H, d, *J* = 1.9 Hz, H-2, H-2′), 3.90 (2H, dd, *J* = 8.7, 6.5 Hz, H-9b, H-9′b), 3.85 (6H, s, 2 × CH_3_), 3.84 (6H, s, 2 × CH_3_), 3.52 (2H, dd, *J* = 8.7, 6.0 Hz, H-9a, H-9′a), 2.63 (2H, dd, *J* = 13.7, 6.0 Hz, H-7b, H-7′b), 2.53 (2H, dd, *J* = 13.7, 8.3 Hz, H-7a, H-7′a), 2.22–2.16 (2H, m, H-8, H-8′); ^13^C-NMR (CDCl_3_, 125 MHz) *δ* 148.9 (C, C-3, C-3′), 147.5 (C, C-4, C-4′), 133.1 (C, C-1, C-1′), 120.6 (CH, C-6, C-6′), 112.1 (CH, C-2, C-2′), 111.3 (CH, C-5, C-5′), 73.4 (CH_2_, C-9, C-9′), 56.0 (2 × CH_3_), 55.9 (2 × CH_3_), 46.7 (CH, C-8, C-8′), 39.2 (CH_2_, C-7, C-7′); EIMS *m*/*z* 372 ([M]^+^, 82), 152 (95), 151 (100), 137 (33), 121 (34), 107 (25); HREIMS *m/z* 372.1942 [M]^+^ (calcd for C_22_H_28_O_5_, 372.1937).

### 3.14. 4-(3R,4R)-4-(3,4-Dimethoxybenzyl)tetrahydrofuran-3-yl)methyl)benzene-1,2-diol *(**11**)*

Following the general procedure for removing the methylenedioxy moiety, 17.1 mg (0.047 mmol) of compound **9** was treated with 32.3 mg (0.239 mmol) of anhydrous AlCl_3_. The residue was purified by silica gel preparative TLC with Hex/EtOAc (1:1) to provide 12.9 mg (80%) of compound **11** as a colorless oil. [α]^20^_D_–27.5 (*c* 0.004, CHCl_3_); ^1^H-NMR (CDCl_3_, 500 MHz) *δ* 6.77 (1H, d, *J* = 8.1 Hz, H-5), 6.72 (1H, d, *J* = 8.1 Hz, H-5′), 6.63 (1H, dd, *J* = 8.2, 1.7 Hz, H-6), 6.56 (1H, s, H-2), 6.54 (1H, s, H-2′), 6.48 (1H, d, *J* = 8.2 Hz, H-6′), 3.94–3.89 (2H, m, H-9b, H-9′b), 3.86 (3H, s, CH_3_), 3.81 (3H, s, CH_3_), 3.55–3.50 (2H, m, H-9a, H-9′a), 2.63–2.43 (4H, m, H-7b, H-7′b, H-7a, H-7′a), 2.20–2.13 (2H, m, H-8, H-8′); ^13^C-NMR (CDCl_3_, 125 MHz) *δ* 148.9 (C, C-3), 147.5 (C, C-4), 143.7 (C, C-3′), 142.0 (C, C-4′), 133.4 (C, C-1′), 133.1 (C, C-1), 121.2 (CH, C-6′), 120.8 (CH, C-6), 115.8 (CH, C-2′), 115.4 (CH, C-5′), 112.1 (CH, C-2), 111.3 (CH, C-5), 73.5 (CH_2_, C-9/C-9′), 73.4 (CH_2_, C-9/C-9′), 56.1 (CH_3_), 56.0 (CH_3_), 46.5 (CH, C-8/C-8′), 46.4 (CH, C-8/C-8′), 39.1 (CH_2_, C-7/C-7′), 38.8 (CH_2_, C-7/C-7′); EIMS *m/z* 344 ([M]^+^, 63), 153 (61), 152 (71), 151 (100), 137 (57), 123 (85), 121 (63), 81 (49), 77 (48), 69 (81), 57 (63), 55 (66); HREIMS *m/z* 344.1619 [M]^+^ (calcd for C_20_H_24_O_5_, 344.1624).

### 3.15. (2R,3R)-2-(Benzo[d][1,3]dioxol-5-ylmethyl)-3-(3,4-dimethoxybenzyl)butane-1,4-diyl Dimethanesulfonate *(**12**)*

To a solution of 91.4 mg (0.244 mmol) of diol **7** and 247 µL (1.75 mmol) of Et_3_N in 3 mL of DCM at 0 °C, 115 µL (1.46 mmol) of MsCl wasadded. After the reaction mixture was stirred at room temperature for 6 h, 10 mL of H_2_O and 10 mL of DCM were added. The organic layer was separated, washed with 10 mL of 2.0 M aqueous HCl solution and 10 mL of a saturated aqueous NaHCO_3_ solution, and dried over anhydrous Mg_2_SO_4_. The solvent was removed and the residue was purified by preparative TLC using Hex/EtOAc (1:1) as eluent to afford 107.4 mg (83%) of compound **12** as a colorless oil. [α]^20^_D_–1.62 (*c* 0.008, CHCl_3_); ^1^H-NMR (CDCl_3_, 500 MHz) *δ* 6.67 (1H, d, *J* = 8.6 Hz, H-5), 6.71 (1H, d, *J* = 7.7 Hz, H-5′), 6.68- 6.65 (2H, m, H-2, H-6), 6.60–6.56 (2H, m, H-2′, H-6′), 5.92 (2H, d, *J* = 1.8 Hz, -OCH_2_O-), 4.25 -4.16 (4H, m, H-9b, H-9a, H-9′b, H-9′a), 3.85 (3H, s, -OCH_3_), 3.83 (3H, s, -OCH_3_), 2.98 (6H, s, Ms), 2.84–2.78 (2H, m, H-7b, H-7′b), 2.62–2.53 (2H, m, H-7a, H-7′a), 2.28–2.20 (2H, m, H-8, H-8′); ^13^C-NMR (CDCl_3_, 125 MHz) *δ* 149.3 (C, C-3), 148.0 (C, C-3′), 147.8 (C, C-4), 146.4 (C, C-4′), 132.3 (C, C-1′), 131.2 (C, C-1), 122.1 (CH, C-6′), 121.1 (CH, C-6), 112.1 (CH, C-2), 111.4 (CH, C-5), 109.2 (CH, C-2′), 108.4 (CH, C-5′), 101.1 (CH_2_), 69.2 (CH_2_, C-9, C-9′), 56.0 (-OCH_3_), 55.9 (-OCH_3_), 40.4 (CH, C-8/C-8′), 40.3 (CH, C-8/C-8′), 37.3 (CH_3_, 2 × Ms), 34.1 (CH_2_, C-7/C-7′), 33.9 (CH_2_, C-7/C-7′); EIMS *m*/*z* 530 ([M]^+^, 100), 434 (99), 338 (99), 326 (99), 203 (99), 189 (99), 187 (99), 152 (99), 151 (99), 136 (99), 135 (99), 96 (99), 79 (99); HREIMS *m/z* 530.1291 [M]^+^ (calcd for C_23_H_30_O_10_S_2_, 530.1280).

### 3.16. 5-((2R,3R)-4-(3,4-Dimethoxyphenyl)-2,3-dimethylbutyl)benzo[d][1,3]dioxole *(**13**)*

A solution of 107.9 mg (0.203 mmol) of dimesylate **12** and 47 mg (1.21 mmol) of NaBH_4_ in 5 mL of HMPA was stirred at 60 °C for 24 h. Then, the reaction mixture was cooled to room temperature and 10 mL of a saturated aqueous NH_4_Cl solution was added. The solution was extracted with EtOAc (3 × 15 mL), the organic layers were separated and dried over anhydrous Mg_2_SO_4_. The solvent was removed and the residue was purified by silica gel preparative TLC with Hex/EtOAc (7:3) as eluent to afford 65.3 mg (94%) of compound **13** as a colorless oil. [α]^20^_D_ −20.4 (*c* 0.004, CHCl_3_); ^1^H-NMR (CDCl_3_, 500 MHz) *δ* 6.76 (1H, d, *J* = 8.0 Hz, H-5), 6.69 (1H, d, *J* = 7.7 Hz, H-5′), 6.63 (1H, dd, *J* = 8.0, 1.8 Hz, H-6), 6.59 (1H, dd, *J* = 1.9 Hz, H-2), 6.57 (1H, dd, *J* = 1.5 Hz, H-2′), 6.53 (1H, dd, *J* = 7.8, 1.7 Hz, H-6′), 5.91 (2H, s, -OCH_2_O-), 3.85 (3H, s, -CH_3_), 3.83 (3H, s, -CH_3_), 2.58–2.51 (2H, m, H-7b, H-7′b), 2.41–2.32 (2H, m, H-7a, H-7′a), 1.80–1.68 (2H, m, H-8, H-8′), 0.81 (3H, d, *J* = 6.8 Hz, H_3_-9), 0.80 (3H, d, *J* = 6.8 Hz, H_3_-9′); ^13^C-NMR (CDCl_3_, 125 MHz) *δ* 148.8 (C, C-3), 147.5 (C, C-3′), 147.2 (C, C-4), 145.5 (C, C-4′), 135.6 (C, C-1′), 134.3 (C, C-1), 121.8 (CH, C-6′), 121.0 (CH, C-6), 112.2 (CH, C-2), 111.1 (CH, C-5), 109.4 (CH, C-2′), 108.0 (CH, C-5′), 100.8 (CH_2_), 56.0 (CH_3_), 55.9 (CH_3_), 41.2 (CH_2_, C-7/C-7′), 41.1 (CH_2_, C-7/C-7′), 38.1 (CH_,_ C-8/C-8′), 37.9 (CH_,_ C-8/C-8′), 14.0 (CH_3_, C-9/C-9′), 13.9 (CH_3_, C-9/C-9′); EIMS *m/z* 342 ([M]^+^, 43), 179 (15), 151 (100), 136 (21), 135 (54), 105 (11), 69 (11), 57 (13); HREIMS *m/z* 342.1830 [M]^+^ (calcd for C_21_H_26_O_4_, 342.1831).

### 3.17. 4-((2R,3R)-4-(3,4-Dimethoxyphenyl)-2,3-dimethylbutyl)benzene-1,2-diol *(**14**)*

Following the general procedure for removing the methylenedioxy, 36.6 mg (0.107 mmol) of **13** were treated with 71.9 mg (0.534 mmol) of anhydrous AlCl_3_. The crude was purified by silica gel preparative TLC with Hex/EtOAc (1:1) to provide 12.3 mg (35%) of compound **14** as a colorless oil. [α]^20^_D_ −14.8 (*c* 0.007, CHCl_3_); ^1^H-NMR (MeOD-*d*_4_, 500 MHz) *δ* 6.80 (1H, d, *J* = 8.6 Hz, H-5), 6.64–6.60 (3H, m, H-2, H-5′, H-6), 6.50 (1H, d, *J* = 1.9 Hz, H-2′), 6.37 (1H, dd, *J* = 8.2, 2.0 Hz, H-6′), 3.78 (3H, s, CH_3_), 3.74 (3H, s, CH_3_), 2.52 (1H, dd, *J* = 13.5, 7.2 Hz, H-7b), 2.44 (1H, dd, *J* = 13.5, 7.5 Hz, H-7′b), 2.38 (1H, dd, *J* = 13.5, 7.8 Hz, H-7a), 2.30 (1H, dd, *J* = 13.5, 7.5 Hz, H-7′a), 1.80–1.74 (1H, m, H-8), 1.72 –1.64 (1H, m, H-8′), 0.81 (3H, d, *J* = 6.8 Hz, H_3_-9), 0.80 (3H, d, *J* = 6.8 Hz, H_3_-9′); ^13^C-NMR (MeOD-*d*_4_, 125 MHz) *δ* 150.2 (C, C-3), 148.5 (C, C-4), 146.9 (C, C-3′), 144.1 (C, C-4′), 135.9 (C, C-1), 134.5 (C, C-1′), 122.3 (CH, C-6), 121.3 (CH, C-6′), 117.0 (CH, C-2′), 116.0 (CH, C-5′), 113.7 (CH, C-2), 112.9 (CH, C-5), 56.5 (CH_3_), 56.3 (CH_3_), 42.1 (CH_2_, C-7), 41.9 (CH_2_, C-7′), 38.9 (CH, C-8′), 38.5 (CH, C-8), 14.2 (CH_3_, C-9/C-9′), 14.1 (CH_3_, C-9/C-9′); EIMS *m*/*z* 330 ([M]^+^, 82), 165 (41), 152 (96), 151 (100), 137 (55), 123 (97), 121 (44), 107 (48), 105 (47); HREIMS *m*/*z* 330.1837 [M]^+^ (calcd for C_20_H_26_O_4_, 330.1831).

### 3.18. (3aR,13aR)-6,7-Dihydroxy-10,11-dimethoxy-3a,4,13,13a-tetrahydro Dibenzo [4,5:6,7]Cycloocta [1,2-c]Furan-1(3H)-one *(**15**)*

A solution of (−)-bursehernin (**1**) (95.5 mg, 0.247 mmol) in 5 mL of dry DCM was added dropwise to a solution of TFA anhydride (0.1 mL) and VOF_3_ (91.8 mg, 0.741 mmol) in 3 mL of TFA-DCM (2:1) at −15 °C under argon. The reaction mixture was stirred from −15 °C to room temperature for 24 h. Then, the reaction was quenched by addition of a saturated aqueous NaHCO_3_ solution (10 mL). The solution was extracted with DCM (3 × 15 mL) and the organic layers were separated and dried over anhydrous Mg_2_SO_4_. The solvent was removed and the residue was purified by silica gel preparative TLC with Hex/EtOAc (3:1) to afford 24.55 mg (28%) of compound **15** as a colorless oil. [α]^20^_D_ −21.4 (*c* 0.005, CHCl_3_); ^1^H-NMR (CDCl_3_, 500 MHz) *δ* 6.72 (2H, s, H-2, H-2′), 6.68 (1H, s, H-5′), 6.64 (1H, s, H-5), 6.15 (2H, brs, 2 × OH), 4.34 (1H, dd, *J* = 8.3, 6.4 Hz, H-9′b), 3.88 (3H, s, CH_3_), 3.82 (3H, s, CH_3_), 3.76 (1H, dd, *J* = 10.9, 8.5 Hz, H-9′a), 3.09 (1H, d, *J* = 13.6 Hz, H-7b), 2.57 (1H, d, *J* = 13.6 Hz, H-7′b), 2.31 (1H, dd, *J* = 13.6, 9.5 Hz, H-7′a), 2.25 (1H, dd, *J* = 13.6, 9.5 Hz, H-7a), 2.10–2.19 (2H, m, H-8, H-8′); ^13^C-NMR (CDCl_3_, 125 MHz) *δ* 177.7 (C, C-9), 148.7 (C, C-3), 147.1 (C, C-4), 143.9 (C, C-3′), 142.1 (C, C-4′), 132.7 (C, C-6′), 132.4 (C, C-6), 131.8 (C, C-1), 131.4 (C, C-1′), 118.1 (CH, C-5′), 115.9 (CH, C-2′), 114.1 (CH, C-5), 111.8 (CH, C-2), 70.5 (CH_2_, C-9′), 56.1 (2 × CH_3_), 50.2 (CH, C-8), 47.2 (CH, C-8′), 33.9 (CH_2_, C-7′), 32.1 (CH_2_, C-7); HRESIMS *m*/*z* 355.1181 [M]^−^ (calcd for C_20_H_19_O_6_, 355.1182).

### 3.19. (3aR,13aR)-6,7,10,11-Tetrahydroxy-3a,4,13,13a-tetrahydrodibenzo [4,5:6,7] Cycloocta [1,2-c]Furan-1(3H)-One *(**16**)*

Following the general demethylation procedure, 26.2 mg (0.071 mmol) of compound **15** was treated with 355 µL (0.355 mmol) of 1 M BBr_3_ solution in DCM. The crude was purified by silica gel preparative TLC with Hex/EtOAc (1:1) to provide 20.1 mg (86%) of compound **16** as a colorless oil. [α]^20^_D_ −28.8 (*c* 0.005, CHCl_3_); ^1^H-NMR (MeOD-*d*_4_, 500 MHz) *δ* 6.66 (1H, s, H-2), 6.64 (1H, s, H-2′), 6.56 (1H, s, H-5′), 6.54 (1H, s, H-5), 4.36 (1H, dd, *J* = 8.3, 6.7 Hz, H-9′b), 3.80 (1H, dd, *J* = 10.6, 8.3 Hz, H-9′a), 2.94 (1H, d, *J* = 12.1 Hz, H-7b), 2.56 (1H, d, *J* = 13.1 Hz, H-7′b), 2.26 (1H, dd, *J* = 13.1, 9.1 Hz, H-7′a), 2.11–2.19 (3H, m, H-7a, H-8, H-8′); ^13^C-NMR (MeOD-*d*_4_, 125 MHz) *δ* 179.6 (C, C-9), 146.0 (C, C-3/C-3′), 145.9 (C, C-3/C-3′), 144.5 (C, C-4/C-4′), 144.4 (C, C-4/C-4′), 133.7 (C, C-6), 133.4 (C, C-6′), 132.5 (C, C-1), 131.9 (C, C-1′), 119.0 (CH, C-5/C-5′), 118.9 (CH, C-5/C-5′), 117.0 (CH, C-2′), 116.5 (CH, C-2), 71.6 (CH_2_, C-9′), 51.6 (CH, C-8), 48.8 (CH, C-8′), 34.7 (CH_2_, C-7′), 32.9 (CH_2_, C-7); HRESIMS *m*/*z* 327.0869 [M]^−^ (calcd for C_18_H_15_O_6_, 327.0869).

### 3.20. Cell Culture

The ERα-positive (ERα+) human breast cancer (BC) cell lines, human ductal carcinoma T47D (HTB 133) and human adenocarcinoma MCF7 (HTB 22) were purchased from ATCC (*American Type Culture Collection*). Cells were maintained in RPMI (Lonza, Basel, Switzerland) growth media, without phenol red, supplemented with 10% fetal bovine serum (FBS) (Lonza), 2 mM glutamine, 10 mM HEPES, 1 mM sodium pyruvate, 100 UI/mL penicillin and 100 μg/mL streptomycin. Cells were growth in a humidified incubator with 5% CO_2_ at 37 °C. When the E2-deprived experiments were carried out, the ERα+ BC cell line was placed in growth media modified by replacement of 10% FBS with 10% dextran-charcoal treated FBS (DCC-FBS) (Biowest, Riverside, MO, USA) one week prior to assay.

### 3.21. Chemical Screening

Chemical screening was carried out by using T47D-KBluc cells, an ER+ BC cell line that is stably transfected with the pGL2.TATA.Inr.Luc.neo containing three ER-responsive elements (ERE) [26]. The T47D-KBluc cells were maintained in standard growth media as detailed above. Dosing media was further modified by reduction of DCC-FBS to 5%. The T47D-KBluc cells were screened using E2 positive, E2 negative (vehicle), antagonist (E2 plus ICI-182.780), and background (vehicle plus ICI-182.780) controls on every plate. For the agonist assessment, cells were treated with test compound, in the absence of E2. For the antagonist assessment, T47D-KBluc cells were incubated with test compound in the presence of pure ERα agonist E2 at 0.1 nM, a concentration corresponding to the maximal luciferase activity (Emax) [26]. To further assess the estrogenic/antiestrogenic activities of test compounds, the dose-effect relationship of E2 (from 0.01 pM to 1 nM) was tested in the absence or in the presence of 5 μM of selected lignans. Chemical screening was also carried out by using the triple negative BC cell line MDA-kb2 cells that stably expresses pMMTV.neo.luc gene, an AR- and GR-responsive reporter gene [34]. The MDA-kb2 cells were screened with test compounds in the absence (vehicle) or presence of 100 nM testosterone (T) or 100 nM dexamethasone (DEX), a dose corresponding to the Emax of T- or DEX-dependent luciferase activity. The T47D-KBluc cells or the MDA-kb2 cells were seeded at 100,000 cells per well in 24-well plates (Nunclon, Sigma-Aldrich, Darmstadt, Germany ) and allowed to attach overnight. Then, the media was replaced with 1 mL/well of dosing media and test compounds incubated 24 h. Then, the cells were harvested in 100 µL passive lysis buffer (Promega, Madison, WI, USA) per well. The relative light units (RLUs), which correspond to luciferase activity from each sample, were measured by using Luciferase Assay Reagent (Promega) in a Clarity luminescence microplate reader (BIOTEK, Winooski, VT, USA). The RLUs from each sample were normalized by protein concentration and converted to fold induction with respect to vehicle-treated control. The maximal increase in luciferase activity was induced by the pure agonist E2 and, therefore, it was considered to be maximal efficacy or Emax. Then, the percent of efficacy (E) of each tested compound, as compared with Emax, was calculated. The antagonists ICI-182.780 [31] and 4-hydroxy-tamoxifen (4-OHTAM) [27] were used as antagonism controls. The agonist or antagonistic effects of lignan derivatives were analyzed by comparing the RLU/mg protein in the absence or in the presence of E2, respectively. Data are expressed as mean ± SEM of triplicate independent experiments, where each treatment was assayed in triplicate. The concentration of tested compound that caused a 50% reduction in Emax (i.e., IC50) and the concentration of pure agonist E2 that induced 50% Emax (i.e., EC50) were obtained by using nonlinear regression analysis in the GraphPad software 8. Duplicate plates were dosed in parallel to control the effects of compounds on cell viability [50]. The protein concentration was measured in cell lysate using colorimetric assay reagent (BioRad, CA, USA) [51].

### 3.22. Cell Viability Assay

The cells were maintained in standard growth media as detailed above and seeded at exponential growth density (6000 cells per well) in 96-well plates (BD Falcon, France). The cells were then treated with vehicle or tests compounds (from 0.01 μM to 10 μM) for 24–72 h. The mitochondrial metabolization of the tetrazolium salt 3-(4,5-methyltiazol-2yl-)-2,5diphenyl-tetrazolium bromide] (MTT) (Applichen, Darmstadt, Germany) was used as an indicator of cell viability [45]. The optical density was measured at 595 nm with an iMark Microplate Reader (BioRad, CA, USA).

### 3.23. Human ERα Competitor Binding Assay

The LanthaScreen^TM^ TR-FRET Nuclear Receptor (NR) binding assay (SelectScreen^TM^ Profiling Service, Life Technologies, Carlsbad, CA, USA) was used for screening of potential binding of lignan derivatives to human ERα [33]. Briefly, the kit uses the recombinant human ERα (rhERα) protein and a tight binding, selective fluorescent ligand, Fluormone^TM^ tracer. The assay is optimized to bind 80% of the tracer for optimal assay without right shiffting IC_50_ values. The rhERα protein and the tracer form rhERα/tracer complex, resulting in a high FP value. Compounds that displace tracer tumble rapidly, resulting in a low FP value but the FP value remains high in the presence of compounds which do not displace the tracer from the complex. The shift in FP values in the presence of test compounds (from 0.010 pM to 20 μM) was used to determine relative affinity of compounds for the rhERα protein. Dose-response competition curves were fitted by nonlinear regression analyses in the GraphPad software 8 (GraphPad Software, San Diego, CA, USA) to obtain IC_50_ values [34].

### 3.24. Rat ER Competitor Binding Assay

Rat uterine cytosol (RUC) was obtained from ten-week-old Sprague Dawley rats, 13–16 days after they were ovariectomized under ketamine (80 mg/kg)/medetomidine (1 mg/kg) anesthesia and buprenorfine (0.05 mg/kg/8 h). The experimental protocols used in this study were revised and approved by the Animal Ethics Committee of the University of Las Palmas de Gran Canaria and authorized by the competent authority of the Canary Islands Govermment (OEBA-ULPGC 40/2020 R1).

Then, uteri were removed, trimmed free of adipose tissue, blotted, weighed, and frozen on liquid nitrogen until used to obtain RUC. Briefly, 50–100 mg of uteri per ml of ice-cold TEGM buffer (10 mM Tris, 1.5 mM EDTA, 10% glycerol, 3 mM MgCl_2_, 1 mM PMSF, 1 mM DTT, pH 7.4) were homogenized by using a Polytron PT3000 homogenizer (Kinematica, Göteborg, Sweden) at 15,000 rpm for 3 burst of 30” each. The homogenate was sedimented at 1000 g for 10 min at 4 °C and the supernatant centrifuged at 105,000× *g* for 60 min at 4 °C to obtain RUC. The protein concentration of the cytosol fraction was adjusted to 2 mg/mL after being determined by Bradford′s method [48]. RUC (100 μL) was incubated with 3 nM [^3^H]E2 (estradiol [2,4,6,7-^3^H(N)], SA 70–115 Ci/mmol, >97% purity) (Perkin Elmer) and increasing concentrations of unlabeled competitors (from 0.1E-9 M to 50 E-6 M) for 18 h at 4 °C [29]. Then, 200 μL of DCC suspension (0.8% charcoal: 0.08% dextran, w:w) in cold TE buffer was added to each tube and incubated for 10 min before DCC was centrifuged at 3000 g for 10 min at 4 °C. The supernatant (200 μL) was obtained to measure total and non-specific bound radioactivity in TRICARB 4810 LSC counter (Perkin Elmer). Relative binding affinity (RBA) was calculated as the ratio (%) of compound and E2 specific binding. The dose-response competition curves (from 0.001 μM to 50 μM) were fitted to four-parameter logistic equations by nonlinear regression analyses in the GraphPad software 8 (GraphPad Software, San Diego, CA, USA) to obtain IC_50_ [34].

### 3.25. Protein Preparation and Docking

The docking studies were performed using Glide v8.6. The X-ray coordinates of hERα ligand binding domains (LBD) were extracted from the Protein Data Bank (PDB code 3ERT). The PDB structures were prepared for docking using the Protein Preparation Workflow (Schrodinger, LLC, New York, NY, USA, 2020), accessible from the Maestro program (Maestro, version 12.3, Schrodinger, LLC, New York, NY, USA, 2020). The substrate and water molecules were removed beyond 5 Å, bond corrections were applied to the cocrystallized ligand, and an exhaustive sampling of the orientations of groups was performed. Finally, the receptors were optimized in Maestro 12.3 by using OPL S_2005 force field before the docking study. In the final stage the optimization and minimization on the ligand–protein complexes were performed and the default value for RMSD of 0.30 Å for non-hydrogen atoms was used. The receptor grids were generated using the prepared proteins, with the docking grids centered at the bound ligand for each receptor. A receptor grid was generated using a 1.00 van der Waals (vdW) radius scaling factor and 0.25 partial charge cutoff. The binding sites were enclosed in a grid box of 20 Å3 without constrains. The three-dimensional structures of the ligands to be docked were generated and prepared using LigPrep as implemented in Maestro 12.3 (LigPrep, Schrodinger, LLC: New York, NY, USA, 2020) to generate the most probable ionization states at pH 7 ± 1 (retaining the original ionization state). In this stage a series of treatments were applied to the structures. Finally, the geometries were optimized using OPLS_2005 force field. These conformations were used as the initial input structures for the docking. The ligands were docked using the extra precision mode (XP) [52] without using any constraints and a 0.80 van der Waals (vdW) radius scaling factor and 0.15 partial charge cutoff. The dockings were carried out with flexibility of the residues of the pocket near to the ligand. The generated ligand poses were evaluated with empirical scoring function implemented in Glide, GlideScore, which was used to estimate binding affinity and rank ligands [53]. The XP Pose Rank was used to select the best-docked pose for each ligand.

### 3.26. Induced Fit Docking

The induced fit docking (IFD) experiment was carried out to confer flexibility to the protein side chains, allowing the ligand to adjust and optimize binding interactions within the active site. The ligands were docked by means of the IFD procedure [54,55] based on the Glide search algorithm using the standard protocol and OPLS3e force field (Induced Fit Docking Protocol 2020, Glide version 8.6, Prime version 5.9, Schrodinger, LLC, New York, NY, 2020). The centroid of the cocrystallized ligand residue was selected as center of the Glide grid (inner box side = 10 Å and outer box side = auto). The ligands were initially docked into the receptor by applying a scaling factor of 0.5 to both ligand and protein van der Waals radius. Up to 20 poses per ligand were collected and the side chains of residues within 5 Å of the ligand were refined with Prime. After the Prime minimization of the selected residues and the ligand for each pose, a Glide redocking of each protein ligand complex structure within 30 kcal/mol of the lowest energy structure was performed.

Ligands were redocked into the newly generated receptor conformations generating up to 10 poses using the extra precision mode (XP) [52] scoring function and reverting the vdW radius scaling factors to their default values. Finally, binding energy (IFDScore) for each output pose was estimated and the poses for each protein–ligand complex were visually inspected.

### 3.27. Molecular Dynamics Simulation

Optimized Potentials for Liquid Simulations (OPLS3e) [56] force field in Desmond Molecular Dynamic System was used in order to study the behavior of the ligand–target complexes. The best IFD docking resulting complexes were solvated with an orthorhombic box of TIP3P (transferable intermolecular potential 3-point) water [57] and counter ions were added creating an overall neutral system simulating approximately 0.15 M NaCl. The ions were equally distributed in a water box. The final system was subjected to a MD simulation up to 20 ns using Desmond [58]. The method selected was NPT (Noose–Hover chain thermostat at 300 K, Martyna–Tobias–Klein barostat method at 1.01325 bar with a relaxation time of 2 ps, isotropic coupling, and a 9 Å radius cut-off was used for coulombic short range interaction) constraints were not applied. During the simulations process, the smooth particle-mesh Ewald method was used to calculate long-range electrostatic interactions. For multiple time step integration, the reversible reference system propagator algorithm (RESPA) was applied to integrate the equation of motion with Fourier-space electrostatics computed every 6 fs, and all remaining interactions computed every 2 fs [59]. The MD simulations were carried out on these equilibrated systems for a time period of 20 ns, frames of energy and trajectory were captured after every 1.2 ps and 9.6 ps, respectively. The quality of MD simulations was assessed using the Simulation Event Analysis tool; ligand–receptor interactions were identified using the Simulation Interaction Diagram tool.

### 3.28. ADME Property Predictions

The physicochemical parameters and ADME descriptors were predicted using the QikProp program version 6.3 (Schrödinger, LLC, NY, 2020) [60] in Fast mode and based on the method of Jorgensen [61,62]. The preparation of compounds and the 2D-to-3D conversion was performed using the LigPrep tool, a module of the Small-Molecule Drug Discovery Suite in the Schrödinger software package, followed by MacroModel v12.3 (Schrödinger, LLC, New York, NY, USA, 2020). A conformational search was implemented using molecular mechanics, followed by a minimization of the energy of each conformer. The global minimum energy conformer of each compound was used as input for the ADME studies.

### 3.29. Statistical Analysis

Data are expressed as mean ± SEM. Statistical analysis was performed by using the Student–Newman–Keuls t-test to determine differences between treatment means and positive or negative controls in assessments. The one-way ANOVA test followed by Tukey’s post hoc test was also used. The dose-response curves were fitted using a logistic equation for the nonlinear regression analysis in GraphPad Prism 8 (GraphPad Software, San Diego, CA, USA).

## 4. Conclusions

Based on molecular docking studies on the ERα receptor a set of lignan derivatives was semisynthesized from the natural butyrolactones bursehernin and matairesinol. The effects of these compounds on ER-mediated transcription were analyzed and some of them (**1**, **3**, **4**, **7**, **9**, **11**, **13**, and **14**) showed antiestrogenic activity. These compounds also affected viability human ER+ breast cancer MCF-7 and T47D cells. A TR-FRET competitive binding assay showed that lignan derivatives interacted with rhERα, reaching compound **14**, the best for binding to the rhERα-LBD (IC_50_ = 0.16 μM). Induced fit docking (IFD) and molecular dynamic simulations suggested an appropriate binding mode which corresponded to the antagonists’ conformations that fitted into the binding pocket, forming polar and hydrophobic interactions similar to the antiestrogen 4-OHTAM. Furthermore, the QikProp module of the Schrödinger software was used as a computational method for analyzing the pharmacokinetic descriptors of the compounds with the best antiestrogenic activities. The results displayed herein pave the way for future design and discovery of more selective and active compounds. Although lignans are considered to be phytoestrogens, there are not many studies that have reported the antiestrogenic capacity of lignans. Compound **14** might be a potential candidate for development as a novel chemopreventive agent for hormone-dependent cancer. Furthermore, these lignan compounds can be considered to be partial agonists/antagonists whose estrogenic/antiestrogenic effects should be studied further in detail to gain a better understanding of their potential benefits in ER-dependent diseases.

## Figures and Tables

**Table 1:** Screening of compounds (**1**–**16**) on ERE-dependent transcriptional activity.

	A		B
	VEH	E2	
	E/Emax (%)	E/Emax (%)	IC_50_ (μM)
Compd	Mean ± SEM	Mean ± SEM	Mean ± SE
**E2**	99.98 ± 11.29	nd	nd
**1**	17.58 ± 5.40	125.75 ± 25.33	16.92 ± 1.21
**2**	12,97 ± 3.91	77.09 ± 12.34	nd ± nd
**3**	24.51 ± 6.53	215.45 ± 36.44	nd ± nd
**4**	15.81 ± 2.15	143.16 ± 42.23	47.72 ± 2.24
**5**	22.74 ± 4.26	202.49 ± 56.61	nd ± nd
**6**	5.86 ± 0.84	142.74 ± 43.44	nd ± nd
**7**	13.14 ± 2.58	120.25 ± 32.33	6.41 ± 1.29
**8**	9.41 ± 1.88	32.86 ± 9.12	nd ± nd
**9**	23.80 ± 6.34	132.68 ± 32.46	5.94 ± 1.17
**10**	3.91 ± 1.55	44.05 ± 13.23	nd ± nd
**11**	12.08 ± 4.33	216.52 ± 54.96	10.34 ± 1.30
**12**	22.20 ± 3.89	268.92 ± 53.60	nd ± nd
**13**	16.70 ± 3.37	174.60 ± 25.67	9.18 ± 1.20
**14**	21.67 ± 4.12	156.84 ± 30.22	nd ± nd
**15**	15.63 ± 5.66	87.16 ± 0.54	nd ± nd
**16**	11.30 ± 3.47	53.42 ± 10.24	nd ± nd

T47D-KBluc cells were seeded in E2-depleted growth media (5%DCC-FBS) and pretreated with lignans at 5 μM (A) or with increasing concentration of lignan derivatives (from 1 μM to 10 μM) (B) for 1 h before addition of 0.1 nM E2 for 18 h. The antagonists ICI (10 nM) and 4-OHTAM (1 μM) were used as antagonism controls. Luciferase activity (relative light units or RLU) from each sample was measured in a luminescence microplate reader, as described in the Material and Methods. Then, RLUs from each sample were normalized by protein concentration and converted to fold induction with respect to the vehicle-treated control. The agonist or antagonist effect of each treatment was analyzed by comparing RLU/mg protein in the absence (VEH) or in the presence of E2, respectively. The maximal increase in luciferase activity or Emax (5.19 ± 1.71 fold induction) was induced by the pure agonist E2 and the efficacy (E) of each treatment, as compared with Emax, was calculated (E/Emax %). The concentration of the tested compound that caused 50% reduction of Emax (i.e., IC_50_) was predicted by using nonlinear regression analysis in the GraphPad software 8. Data are expressed as mean ± SE for at least three independent experiments, where each treatment is tested in triplicate; nd indicates that the IC_50_ values is not determined.

**Table 2:** Effects of lignans **1**, **3**, **4**, **7**, **8**, **9**, **11**, **13,** and **14** on potency (EC_50_) and efficacy (Emax) of E2-induced transcription.

	EC_50_ (nM)	Emax (%)
Compd	Mean ± SE	Mean ± SE
**VEH**	0.004 ± 0.0001	96.02 ± 5.63
**1**	0.097 ± 0.032	55.12 ± 7.71
**3**	0.052 ± 0.022	58.45 ± 6.80
**4**	0.102 ± 0.019	56.33 ± 5.41
**7**	0.479 ± 0.018	48.83 ± 2.90
**8**	0.004 ± 0.020	87.49 ± 12.72
**9**	2.110 ± 0.349	41.27 ± 6.82
**11**	0.130 ± 0.029	54.61 ± 8.16
**13**	0.353 ± 0.020	48.65 ± 5.82
**14**	0.032 ± 0.259	35.89 ± 3.48

**Table 3:** Lignan derivatives bind to human ERα.

	IC_50_ (μM)
Competitor	Mean ± SE
E2	0.00005 ± 0.00001
DES	0.00004 ± 0.00001
TAM	0.00004 ± 0.00001
1	0.39 ± 0.19
3	0.60 ± 0.11
4	6.04 ± 1.72
5	3.24 ± 2.03
7	4.00 ± 1.60
9	7.65 ± 1.77
11	0.50 ± 0.17
13	5.02 ± 1.54
14	0.16 ± 0.11

The binding of competitors to rhERα was evaluated in 10-point titration competition curves (from 0.010 pM to 20 μM) by using the LanthaScreen TR-FRET competitive binding assay, as described in the Material and Methods. The concentration of tested compound that caused 50% inhibition of Fluormone^TM^ tracer binding to rhERα (i.e., IC_50_) was determined by using non-linear regression analysis in the GraphPad software 8. Data are expressed as mean ± SEM from two independent experiments where each concentration was tested in duplicate.

**Table 4:** Computational pharmacokinetic parameters (ADME) of lignan derivatives **1**, **3**, **4**, **7**, **9**, **11**, **13**, and **14**.

Property/ Descriptor	1	3	4	7	9	11	13	14	Range/Recommended Values
Mol MW	370.40	330.34	358.39	374.43	356.42	344.41	342.434	330.423	130.0 to 725.0
donorHB	0	4	2	2	0	2	0	2	0.0 to 6.0
accptHB	6	6	6	6,4	4,7	4,7	3	3	2.0 to 20.0
QPlogPo/w	3.16	1.02	2.55	3.33	3.15	3.46	4.31	4.14	−2.0 to 6.5
QPlogS	−2.38	−2.82	−3.63	−3.56	−4.98	−4.00	−6.78	−4.13	−6.5 to 0.5
QPPCaco	3614.92	34.43	308.61	1560.41	9906.04	1104.89	9906.04	1214.07	<25 poor, >500 great
QPPMDCK	1984.22	12.97	138.82	800.24	5899.29	551.02	5899.29	610.11	<25 poor, >500 great
QPlogKhsa	−0.17	−0.26	0.06	0.03	0.23	0.28	0.74	0.52	−1.5 to 1.5
QPlogBB	−0.16	−2.22	−1.35	−0.88	−1.16	−0.82	−0.34	−0.81	−3.0 to 1.2

Mol MW (molecular weight), donorHB (number of hydrogen bond donors), accptHB (number of hydrogen bond acceptors), QPlogPo/w (predicted octanol/water partition coefficient), QPlogS (predicted aqueous solubility), QPPCaco (predicted human epithelial colorectal adenocarcinoma cell line permeability in nm/s), QPPMDCK (predicted Madin–Darby canine kidney permeability in nm/s), QPlogKhsa (predicted binding to human serum albumin), QPlogBB (predicted brain/blood partition coefficient).

## Data Availability

Data is contained within the article or Supplementary Materials.

## Supplementary Materials

The following supporting information can be downloaded at https://www.mdpi.com/article/10.3390/ph15050585/s1: ^1^HNMR and ^13^C NMR of compounds **1**–**16**, Docking of compounds **3** and **11**, and Computacional pharmacokinetic parameters.
