# Supplementary material for: Design, Semisynthesis, and Estrogenic Activity of Lignan Derivatives from Natural Dibenzylbutyrolactones
Source: Pharmaceuticals (Basel). 2022 May 9;15(5):585. doi: 10.3390/ph15050585 (PMC9145393; doi:10.3390/ph15050585)

Supplementary Material

Design, semisynthesis and estrogenic activity of lignan derivatives from natural dibenzylbutyrolactones

Priscila López-Rojas[1], Ángel Amesty[1,*], Miguel Guerra-Rodríguez[2], Yeray Brito-Casillas[2], Borja Guerra,[2] Leandro Fernández-Pérez,[2,*] Ana Estévez-Braun[1,*]

[1]Instituto Universitario de Bio-Orgánica Antonio González, Departamento de Química Orgánica, Universidad de La Laguna, Avda. Astrofísico Fco. Sánchez 2. 38206. La Laguna, Tenerife, Spain.

[2]Instituto Universitario de Investigaciones Biomédicas y Sanitarias (IUIBS), Universidad de Las Palmas de Gran Canaria, Blas Cabrera Felipe s/n, 35016, Las Palmas de Gran Canaria, Spain.

Contents

^{1}H-NMR and ^{13}C-NMR spectra of compounds **1-16.**

^{1}H-NMR (CDCl$_3$, 500 MHz) of (–)-bursehernin (**1**).

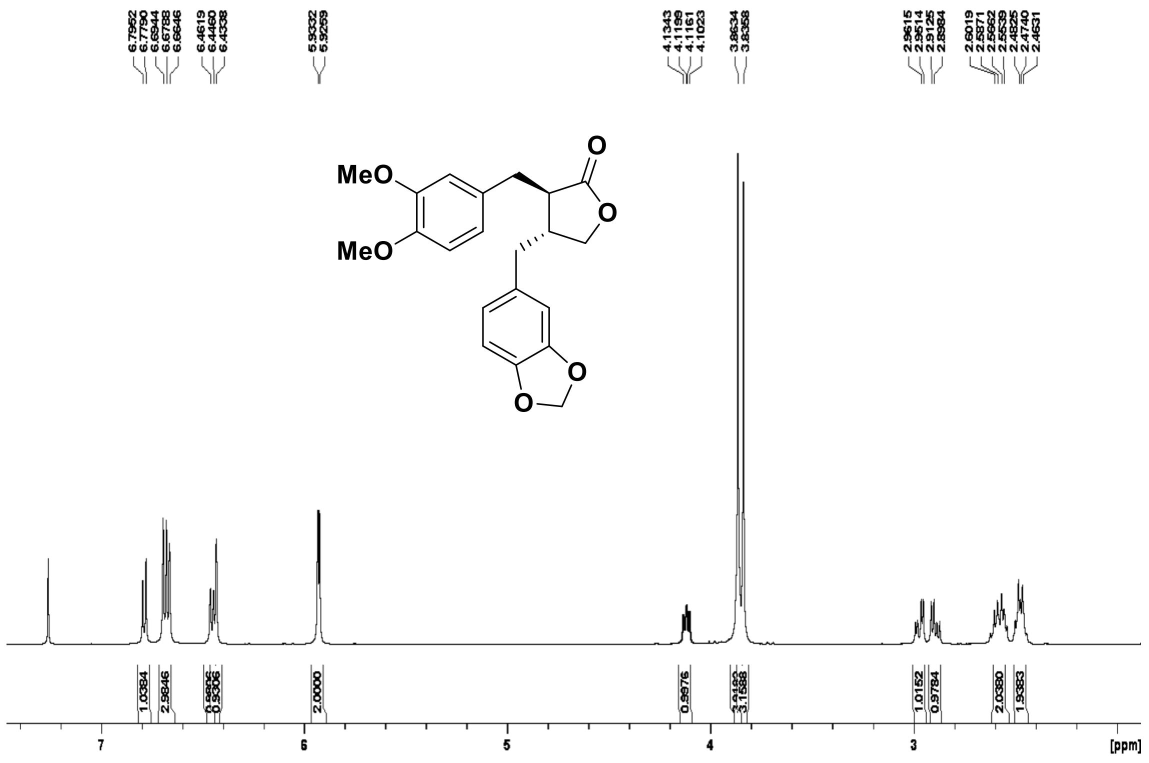

^{13}C-NMR (CDCl$_3$, 500 MHz) of (–)-bursehernin (**1**).

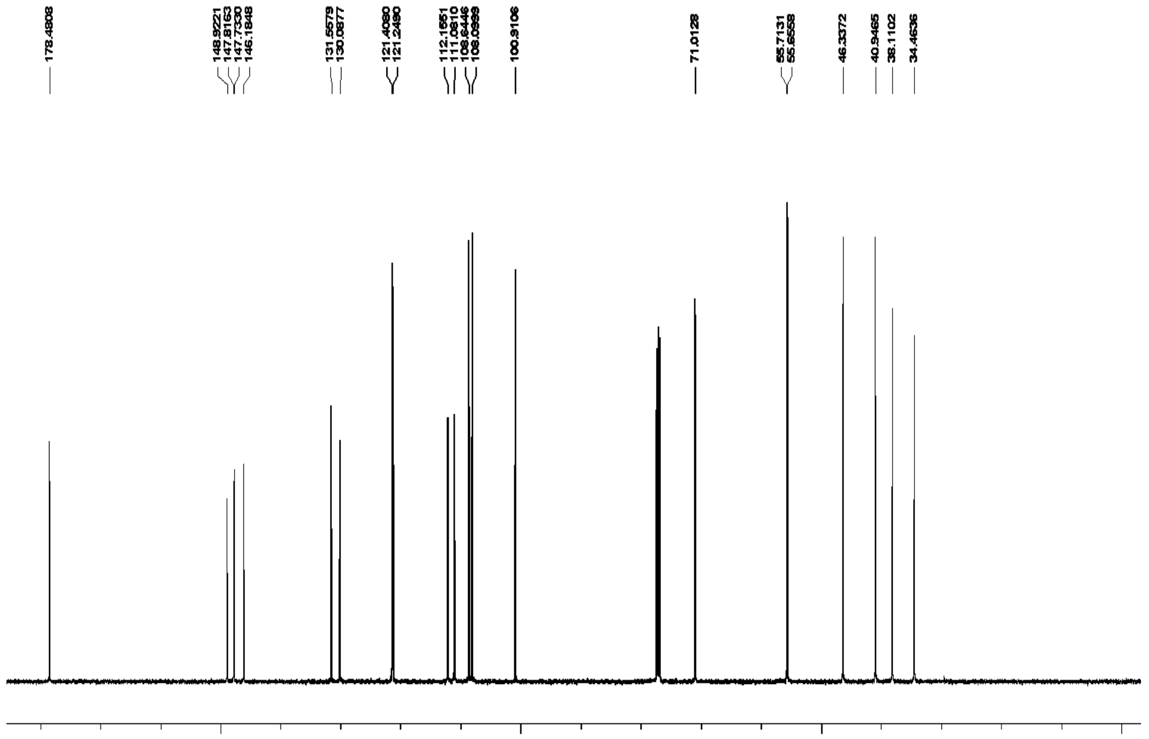

^{1}H-NMR ($CDCl_3$, 500 MHz) of (–)-matairesinol dimethyl ether (**2**).

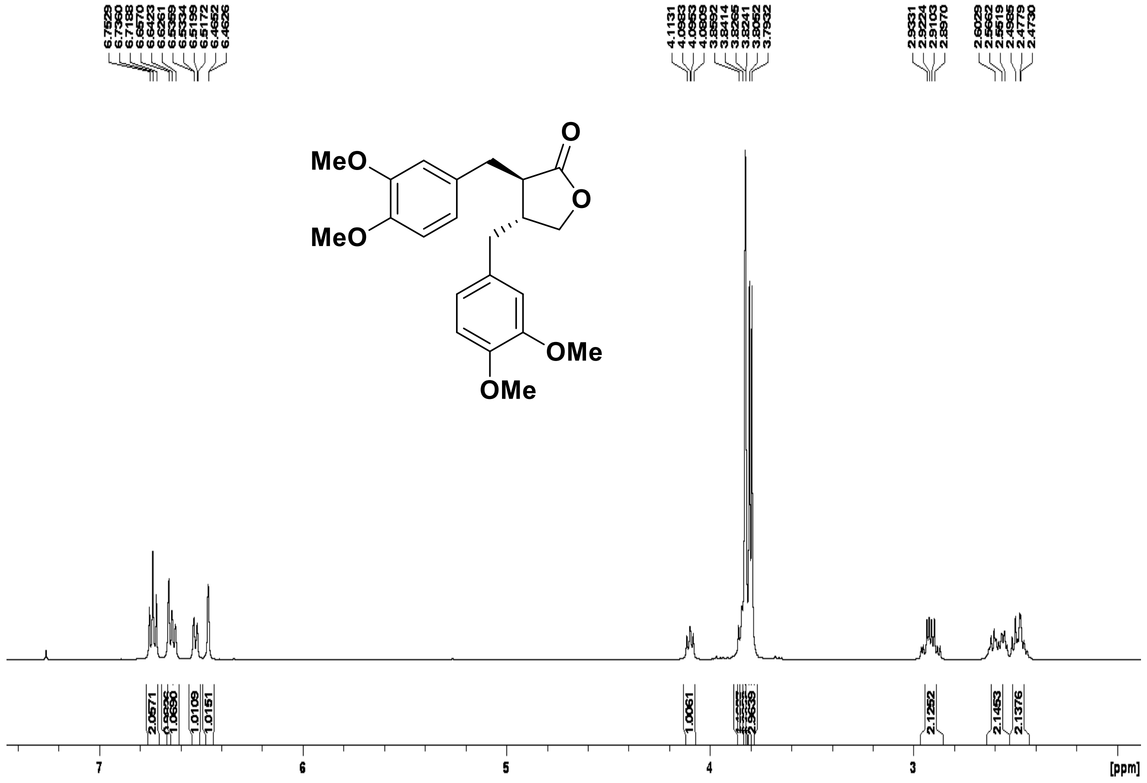

^{13}C-NMR ($CDCl_3$, 500 MHz) of (–)-matairesinol dimethyl ether (**2**).

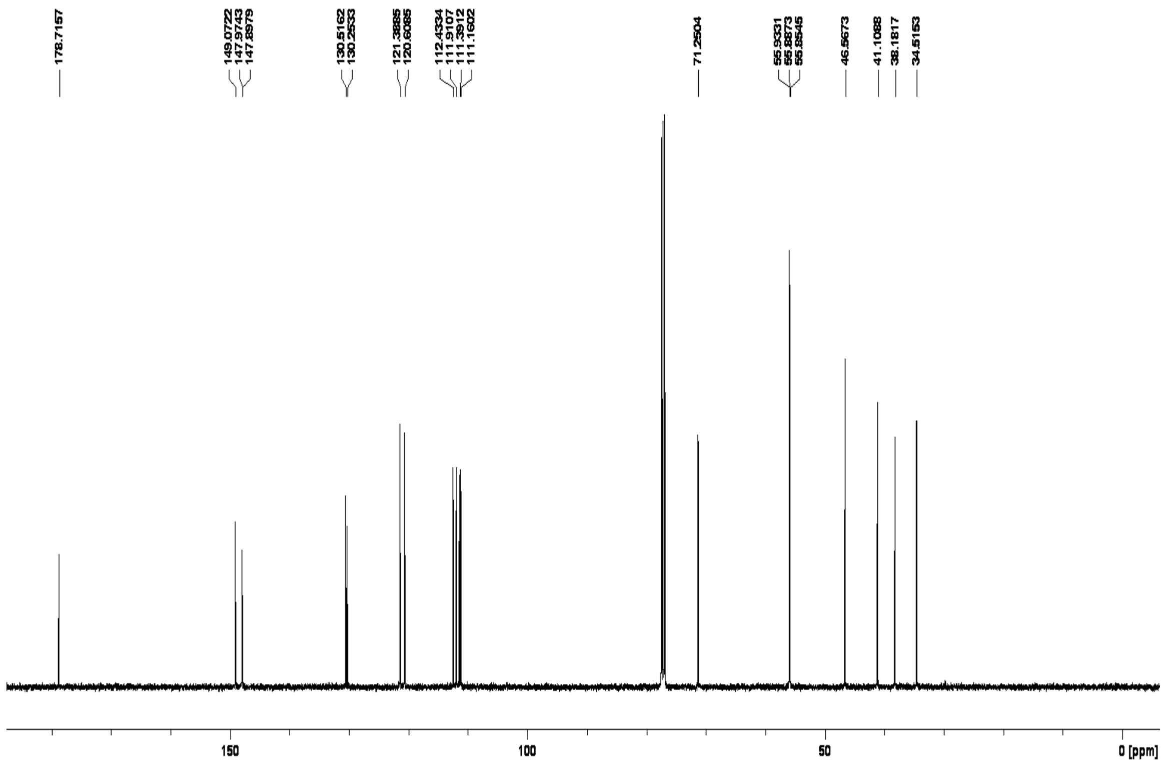

^{1}H-NMR (MeOD-d$_4$, 500 MHz) of compound **3**.

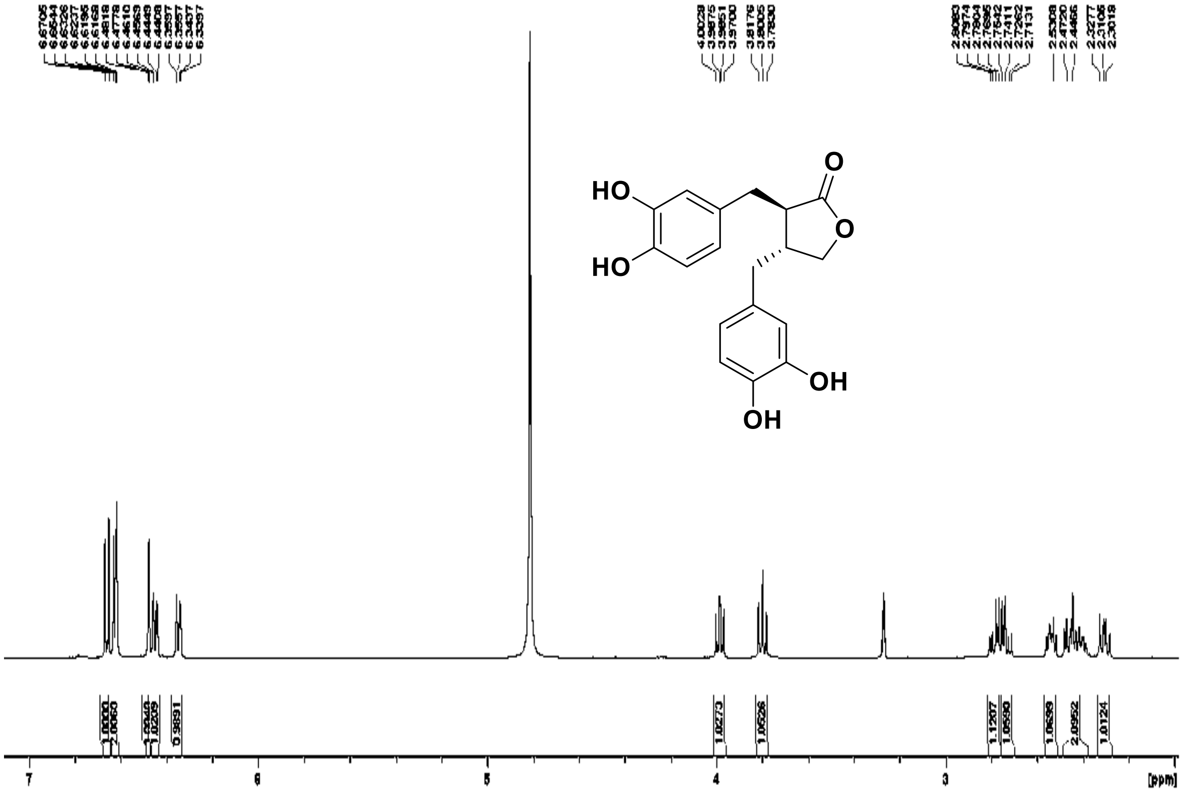

^{13}C-NMR (MeOD-d$_4$, 500 MHz) of compound **3**.

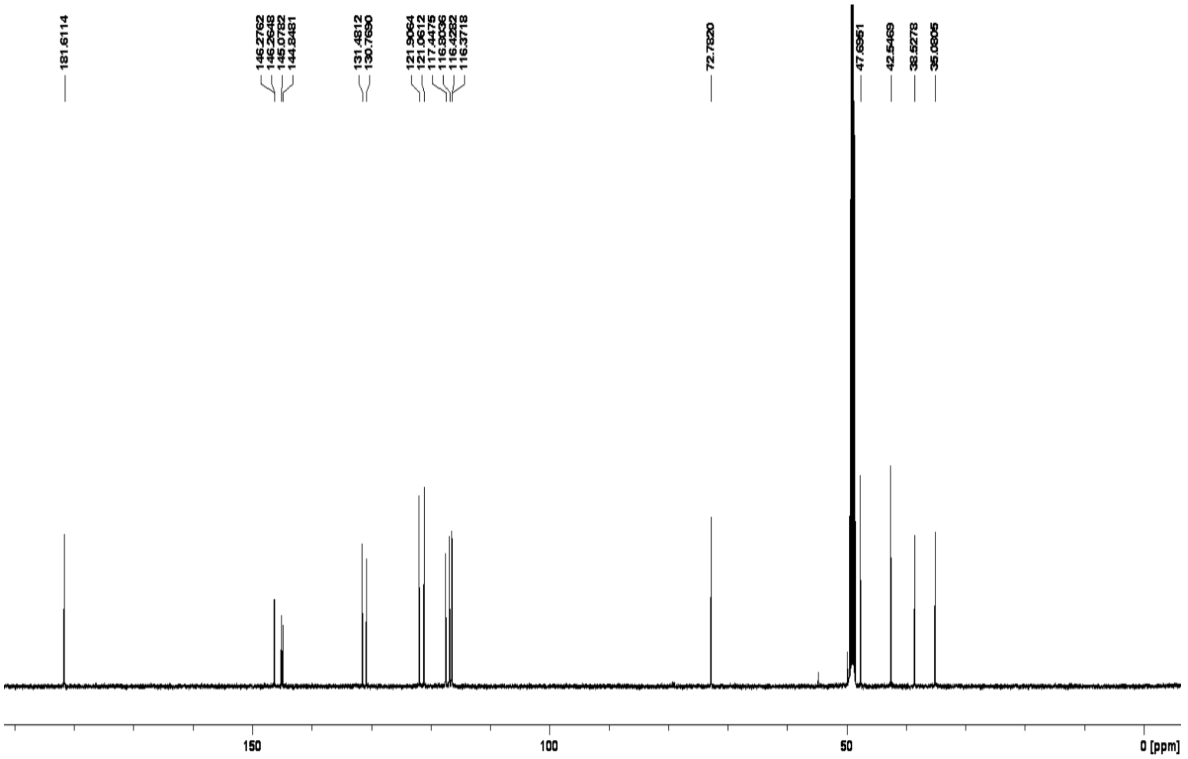

^{1}H-NMR ($CDCl_3$, 500 MHz) of compound **4**.

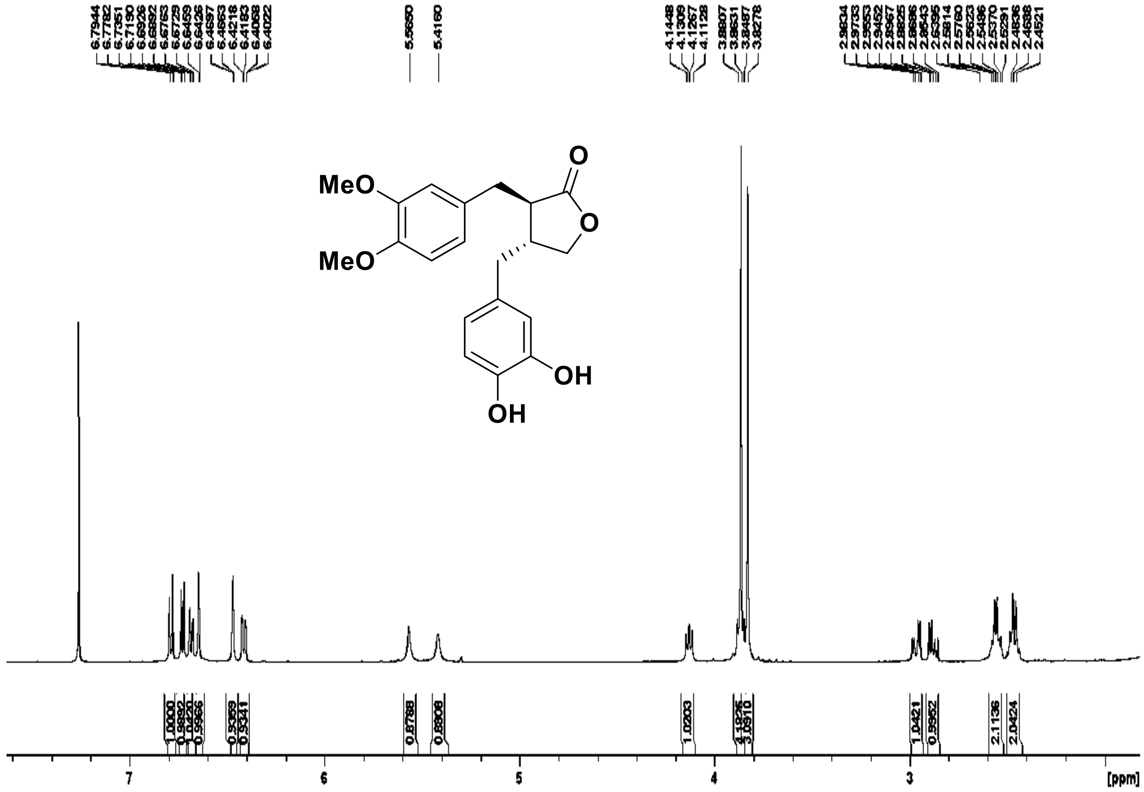

^{13}C-NMR ($CDCl_3$, 500 MHz) of compound **4**.

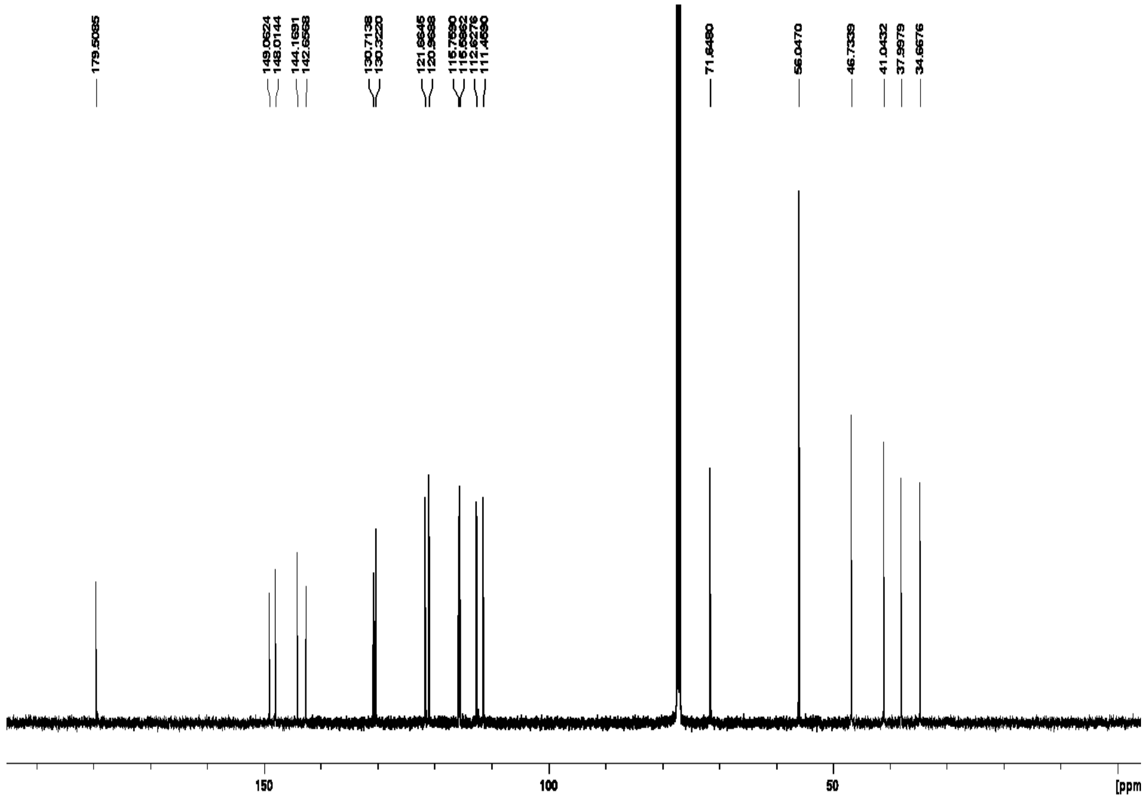

^{1}H-NMR ($CDCl_3$, 500 MHz) of compound **5**.

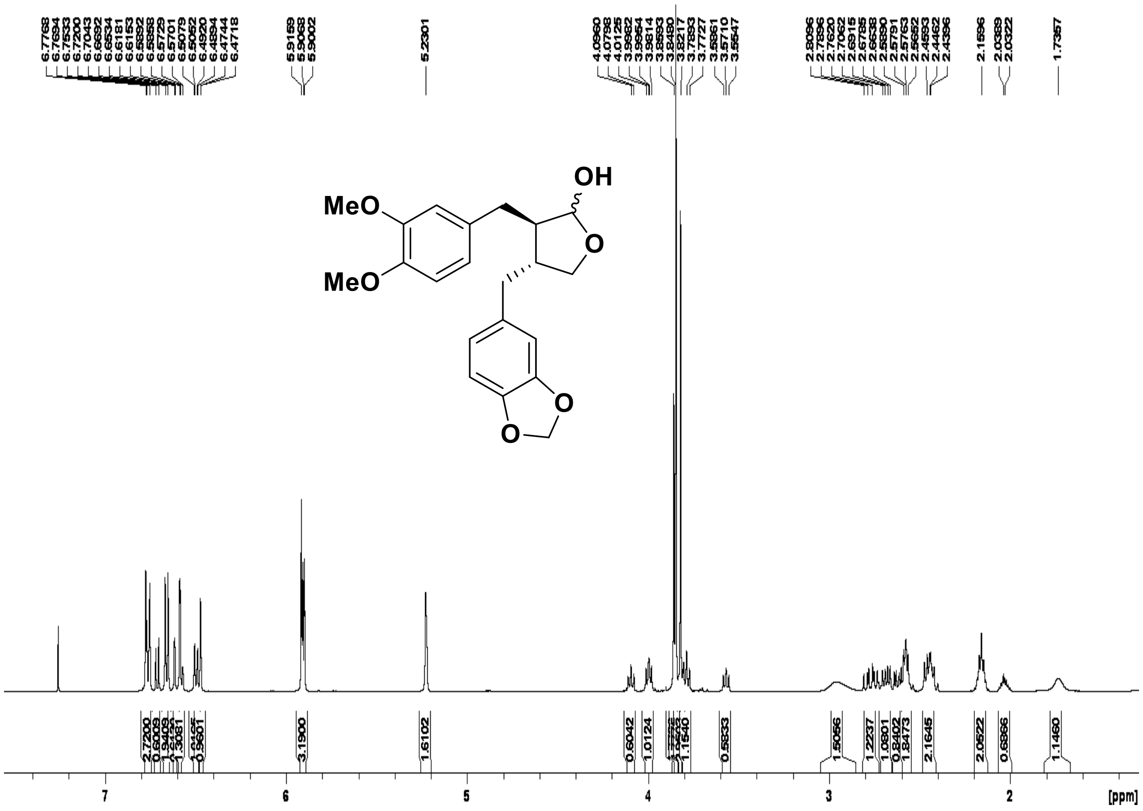

^{13}C-NMR ($CDCl_3$, 500 MHz) of compound **5**.

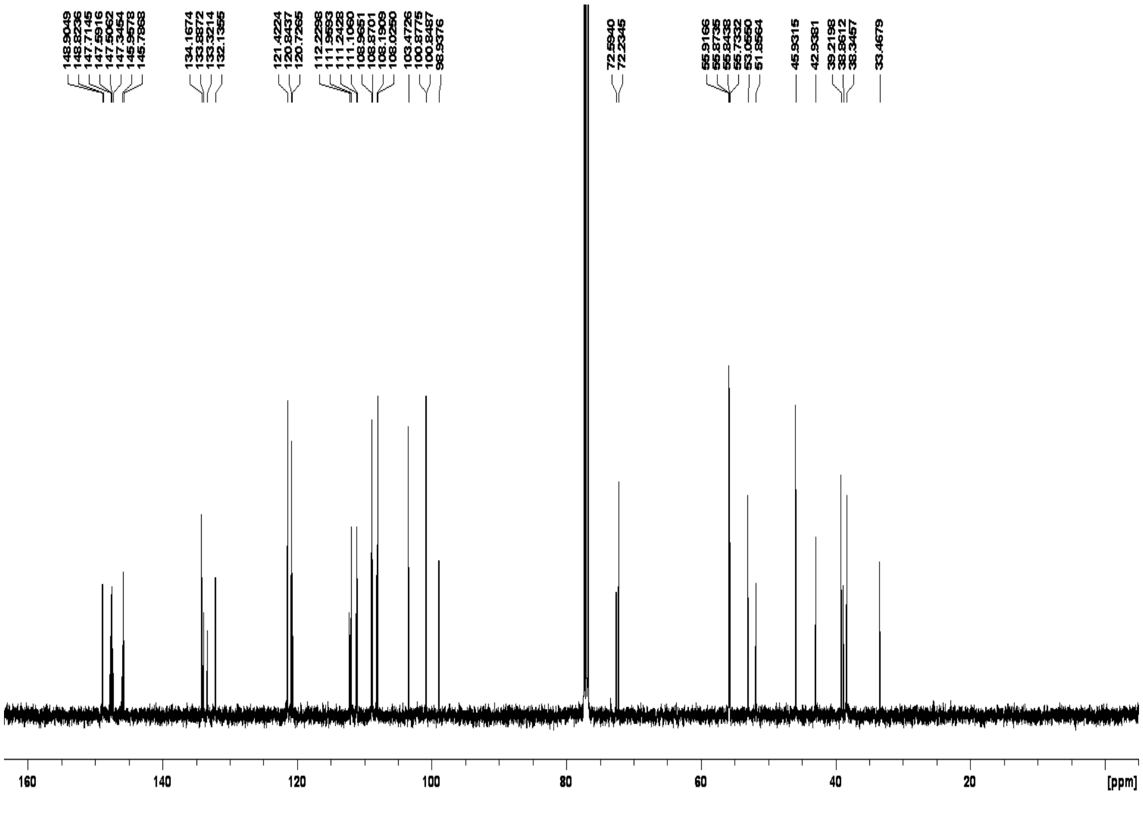

^{1}H-NMR ($CDCl_3$, 500 MHz) of compound **6**.

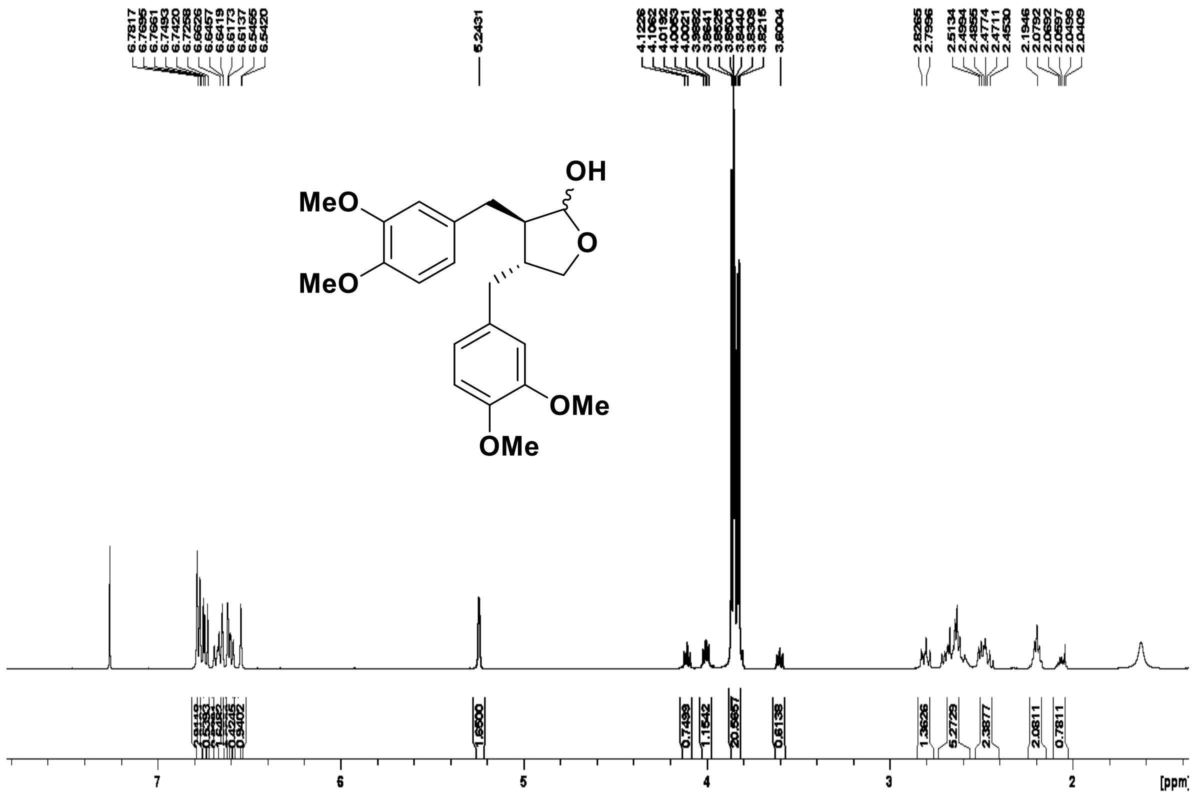

^{13}C-NMR ($CDCl_3$, 500 MHz) of compound **6**.

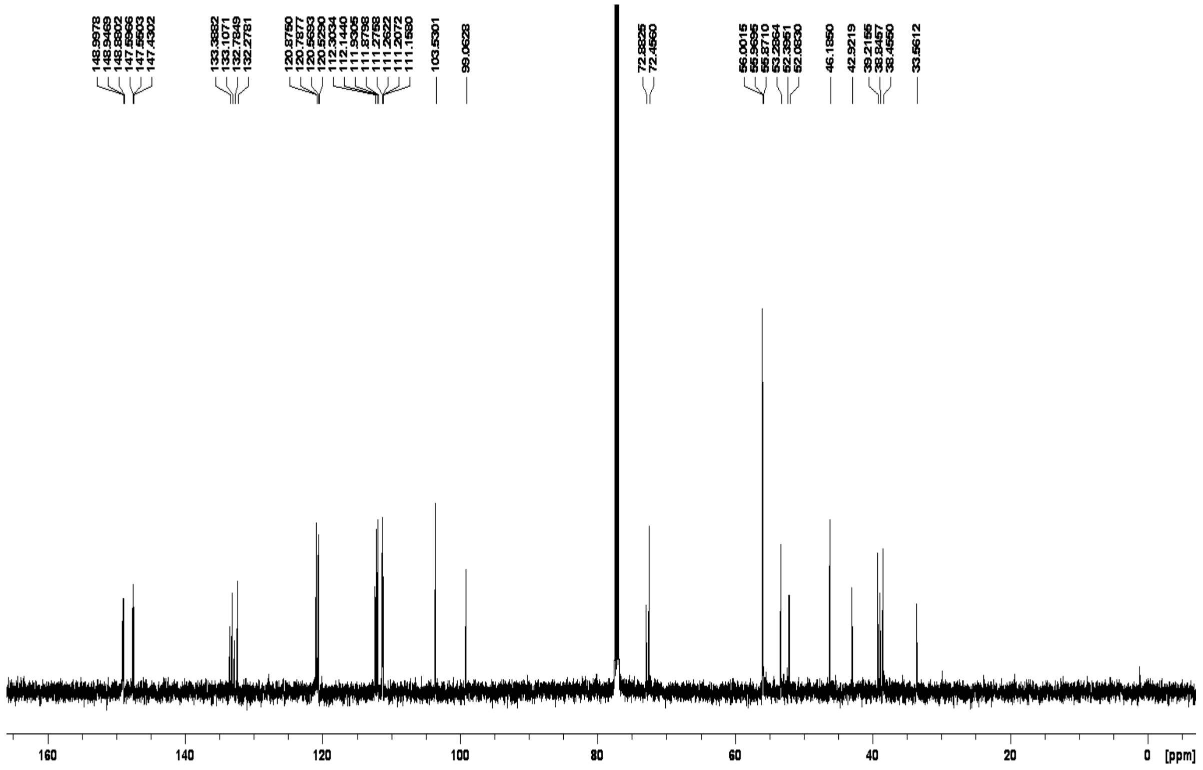

^{1}H-NMR ($CDCl_3$, 500 MHz) of compound **7**.

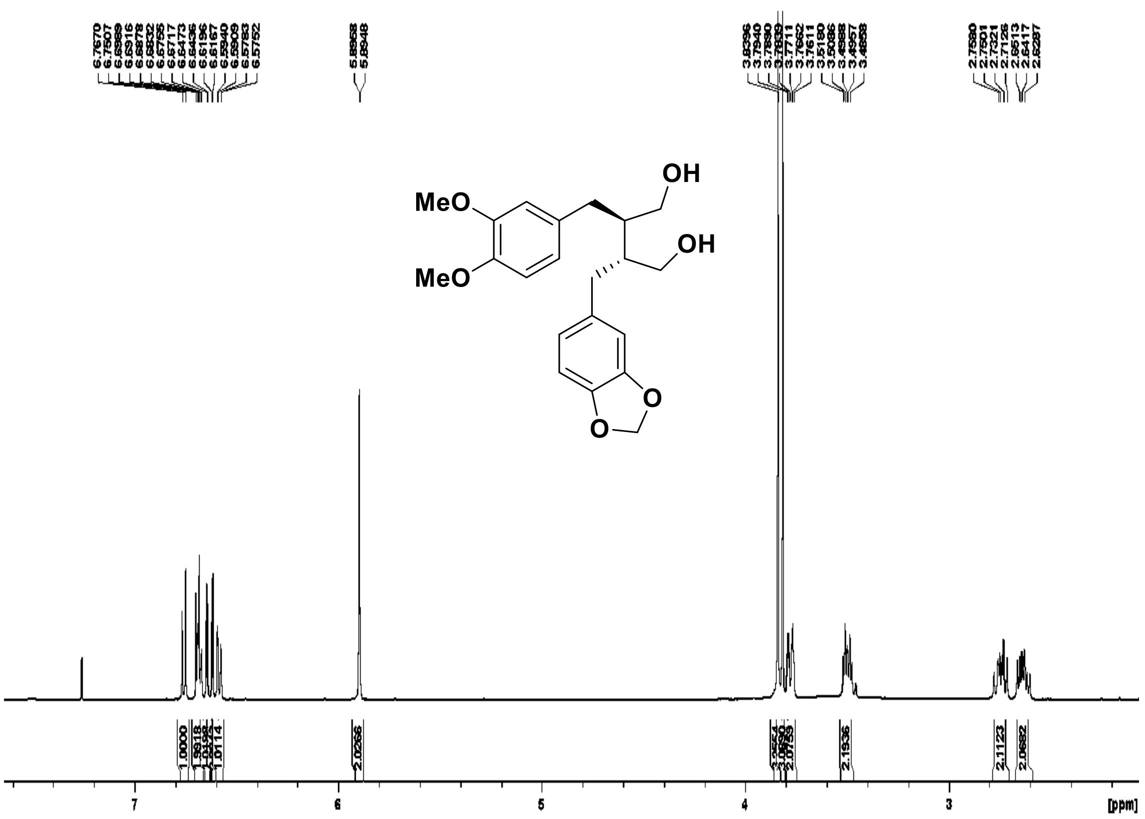

^{13}C-NMR ($CDCl_3$, 500 MHz) of compound **7**.

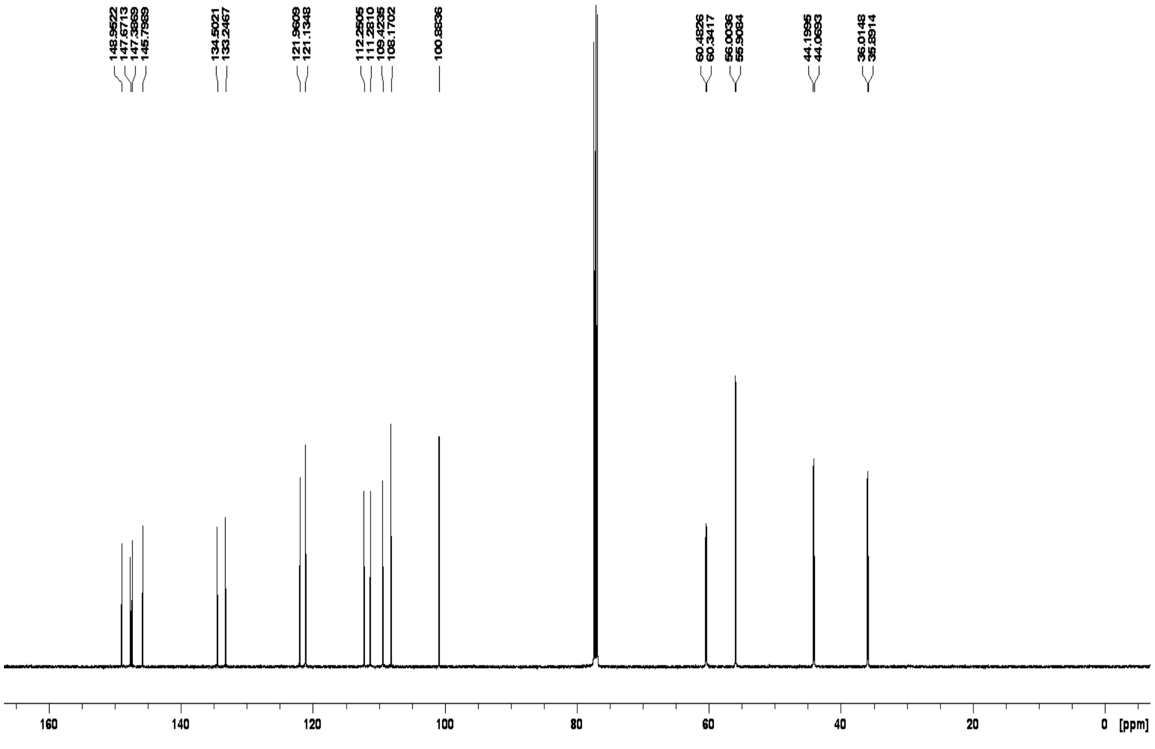

^{1}H-NMR ($CDCl_3$, 500 MHz) of compound **8**.

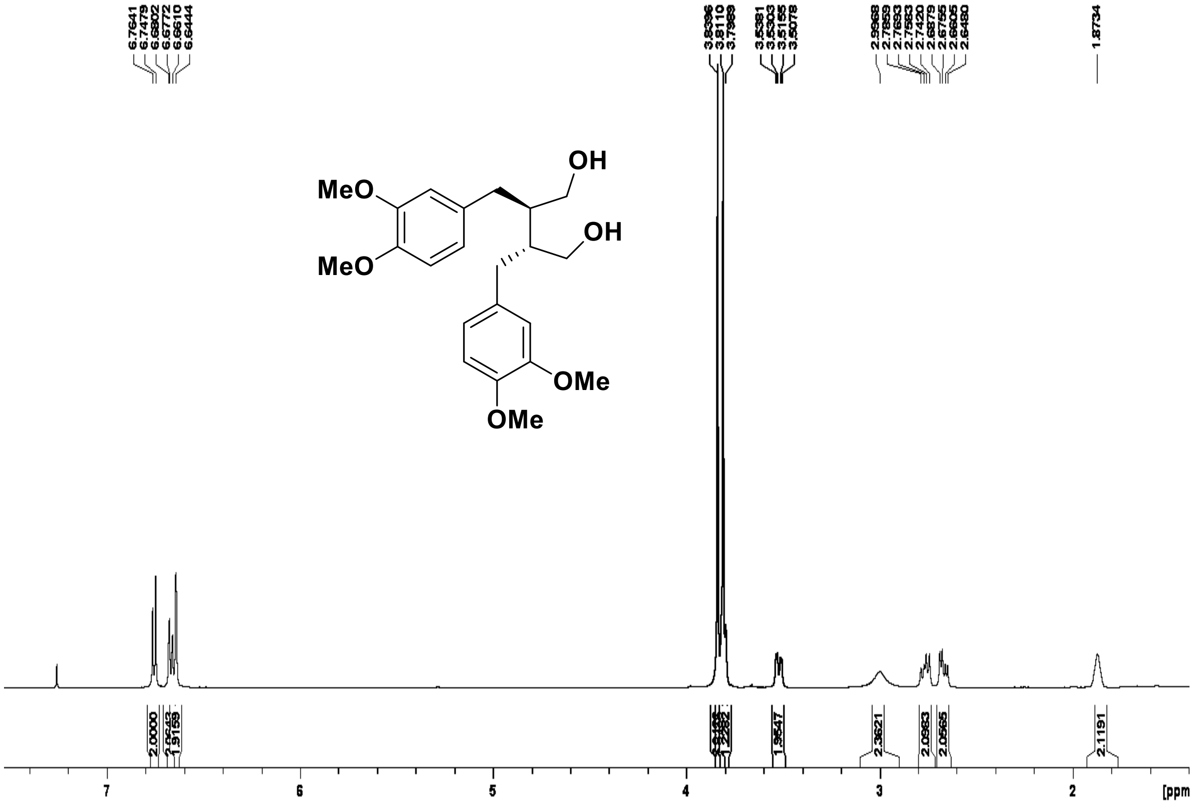

^{13}C-NMR ($CDCl_3$, 500 MHz) of compound **8**.

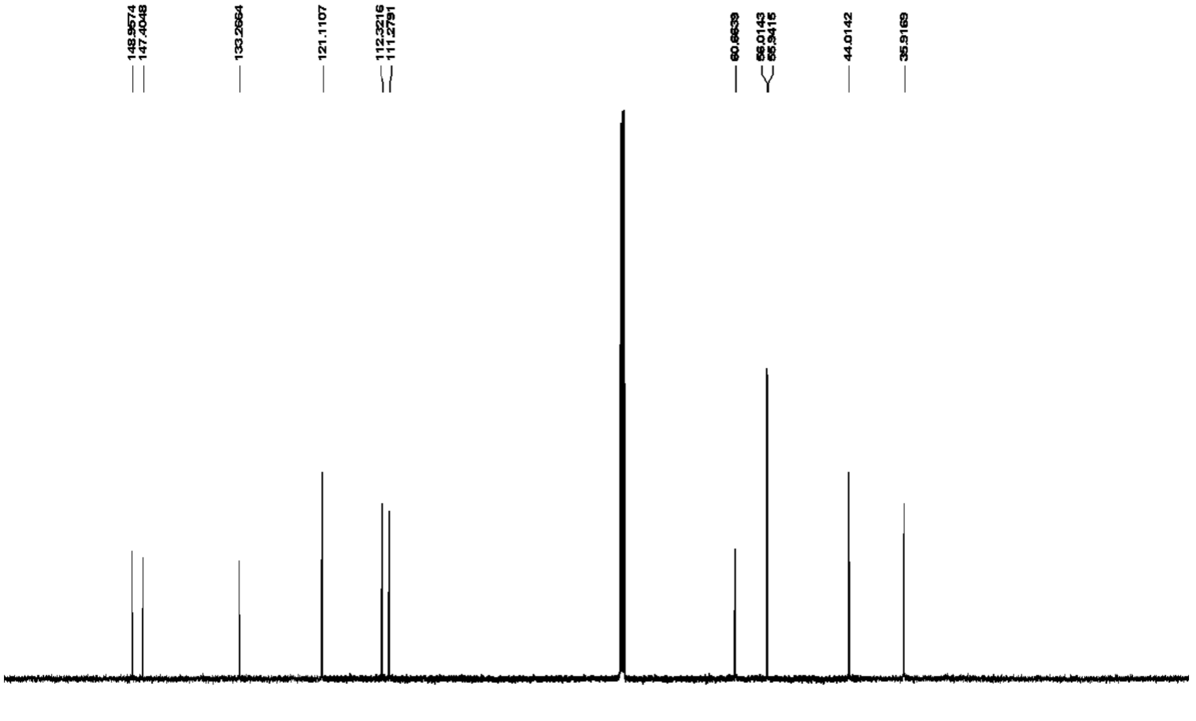

^{1}H-NMR ($CDCl_3$, 500 MHz) of compound **9**.

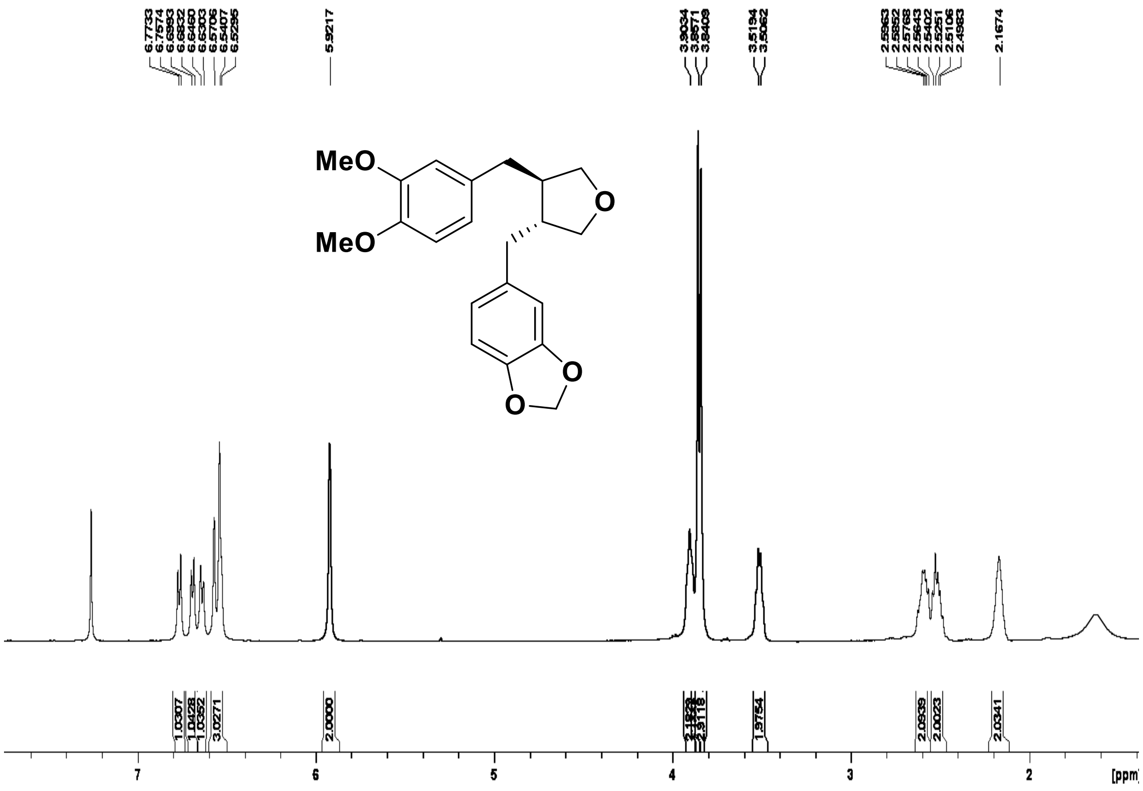

^{13}C-NMR ($CDCl_3$, 500 MHz) of compound **9**.

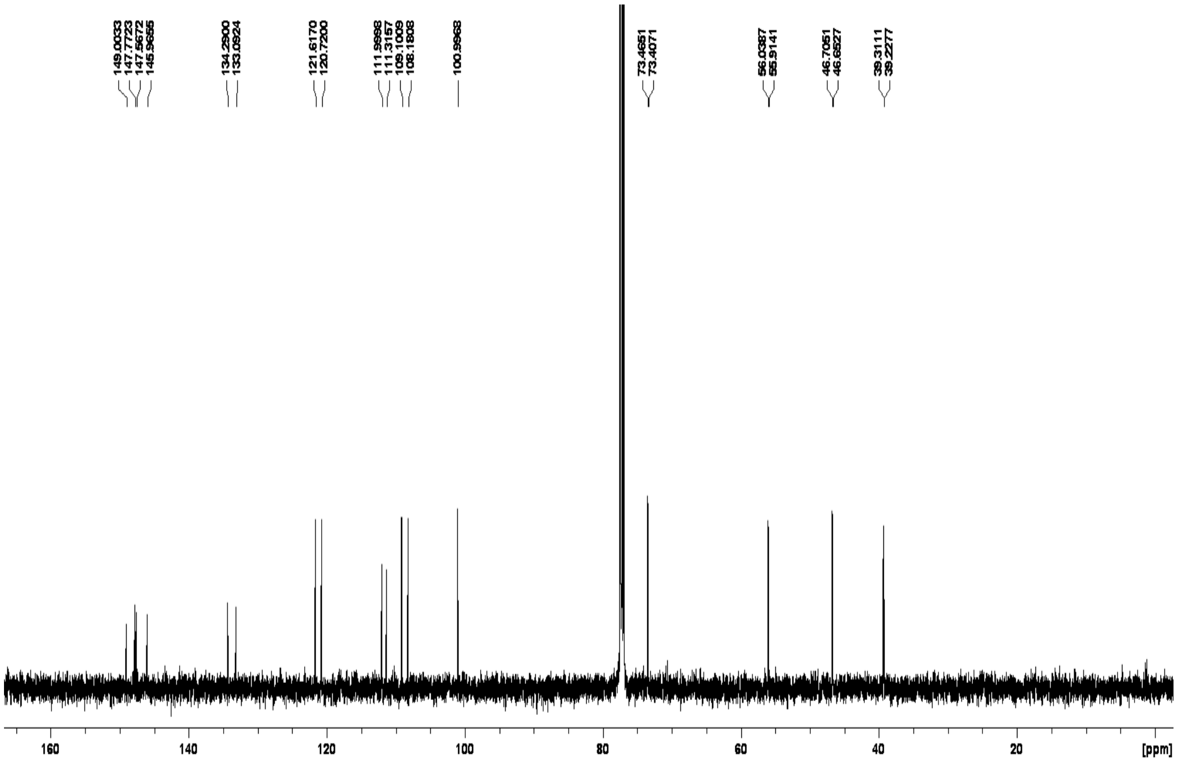

^{1}H-NMR ($CDCl_3$, 500 MHz) of compound **10**.

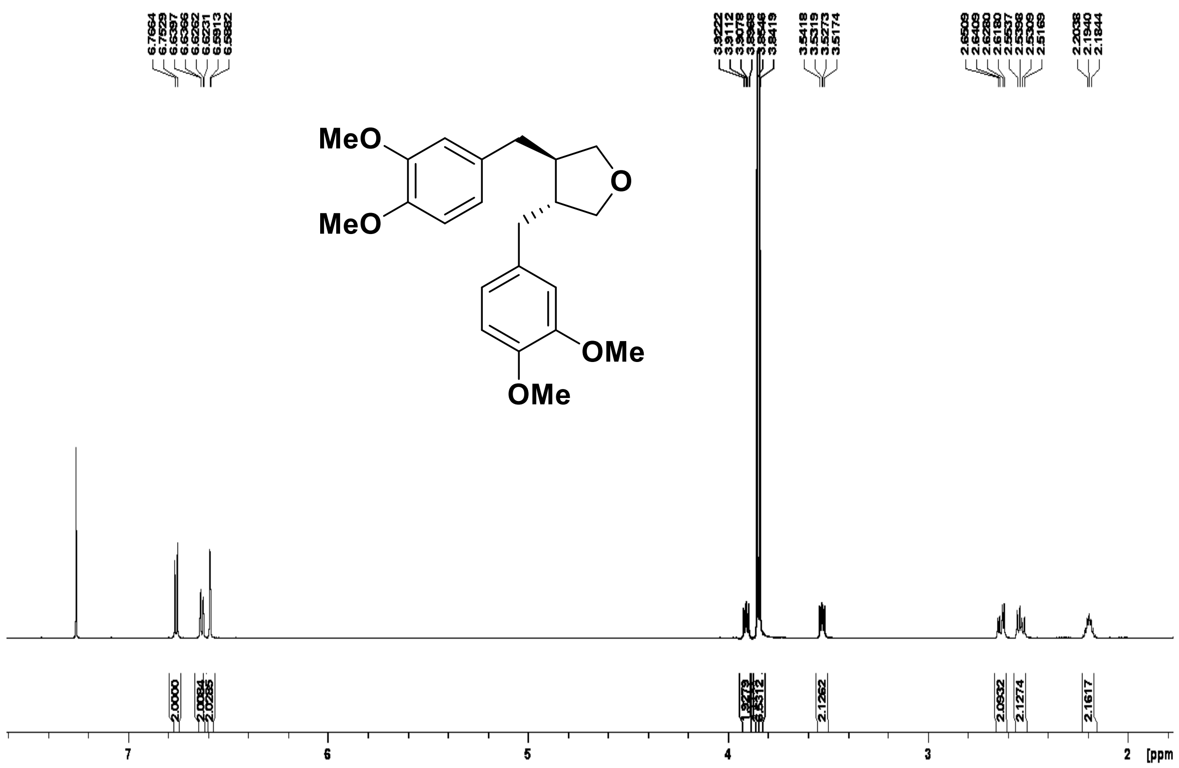

^{13}C-NMR ($CDCl_3$, 500 MHz) of compound **10**.

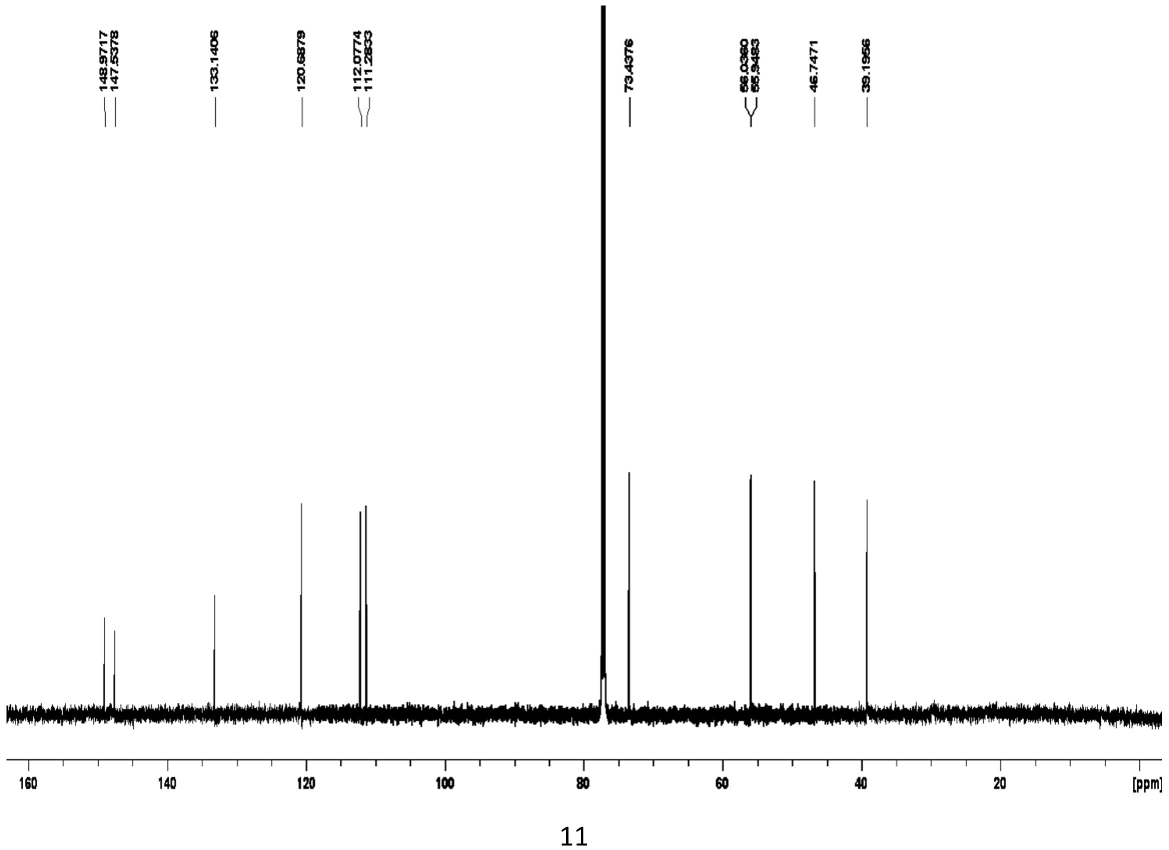

^{1}H-NMR ($CDCl_3$, 500 MHz) of compound **11**.

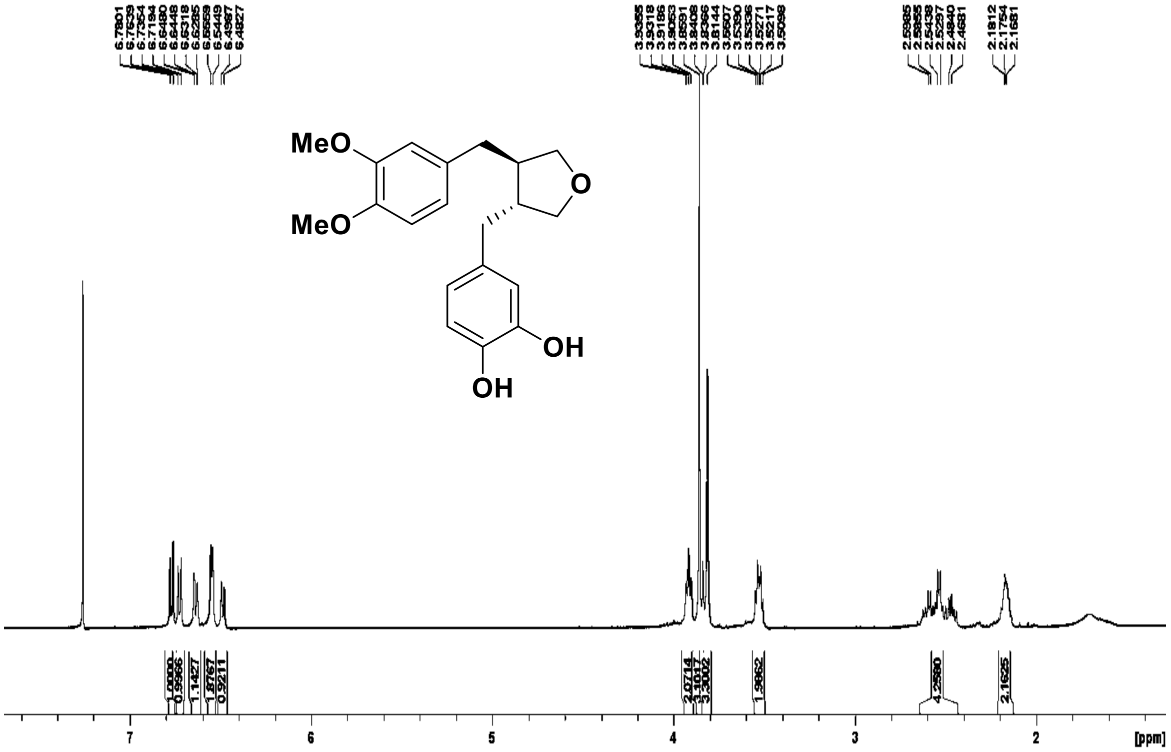

^{13}C-NMR ($CDCl_3$, 500 MHz) of compound **11**.

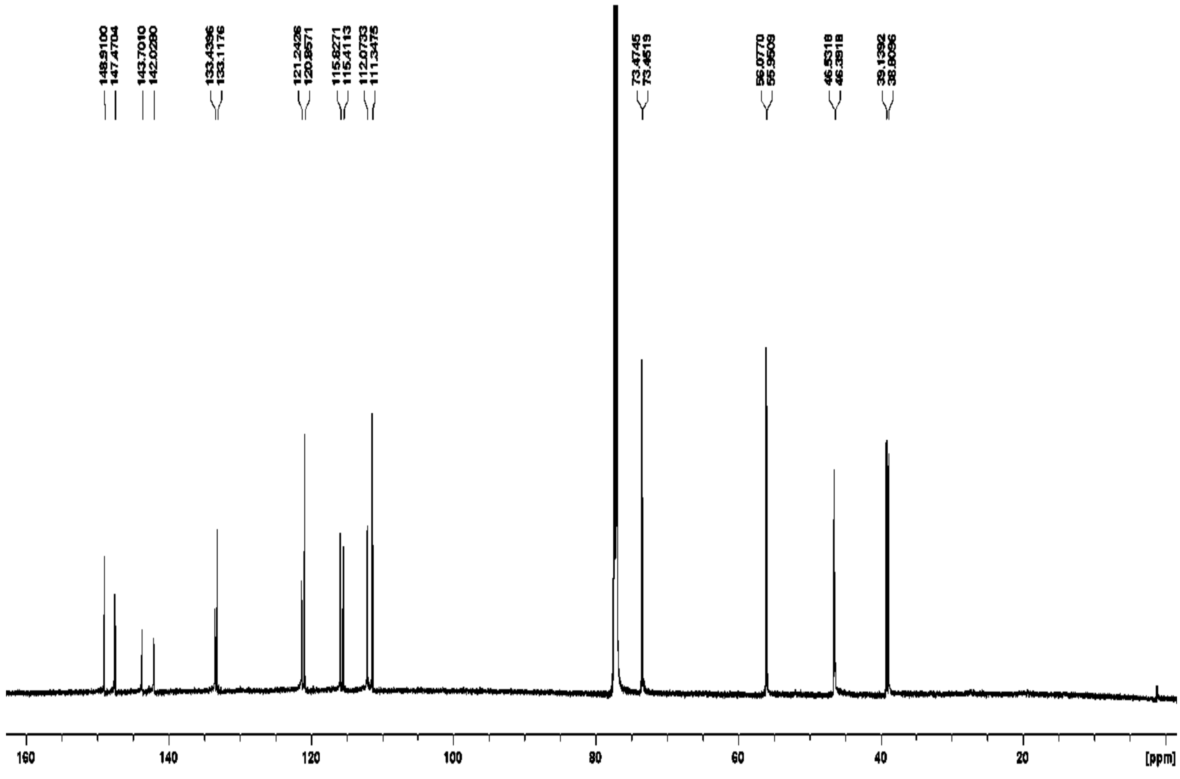

^{1}H-NMR ($CDCl_3$, 500 MHz) of compound **12**.

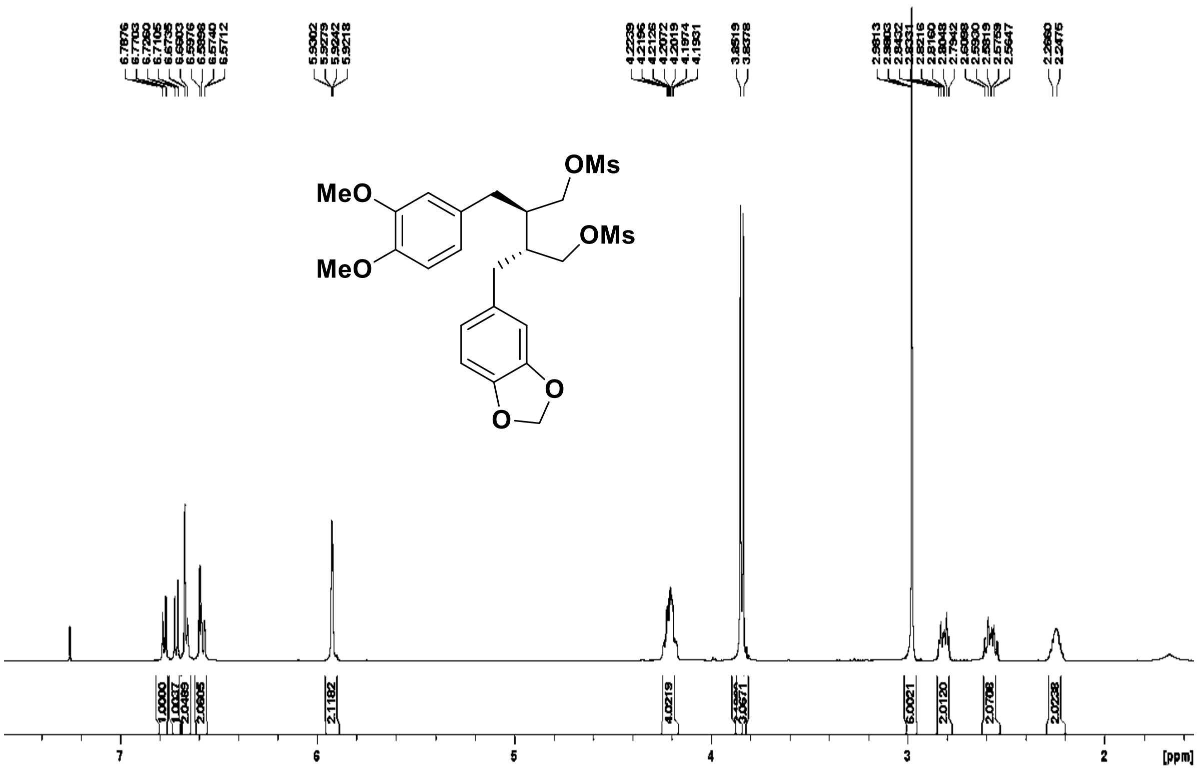

^{13}C-NMR ($CDCl_3$, 500 MHz) of compound **12**.

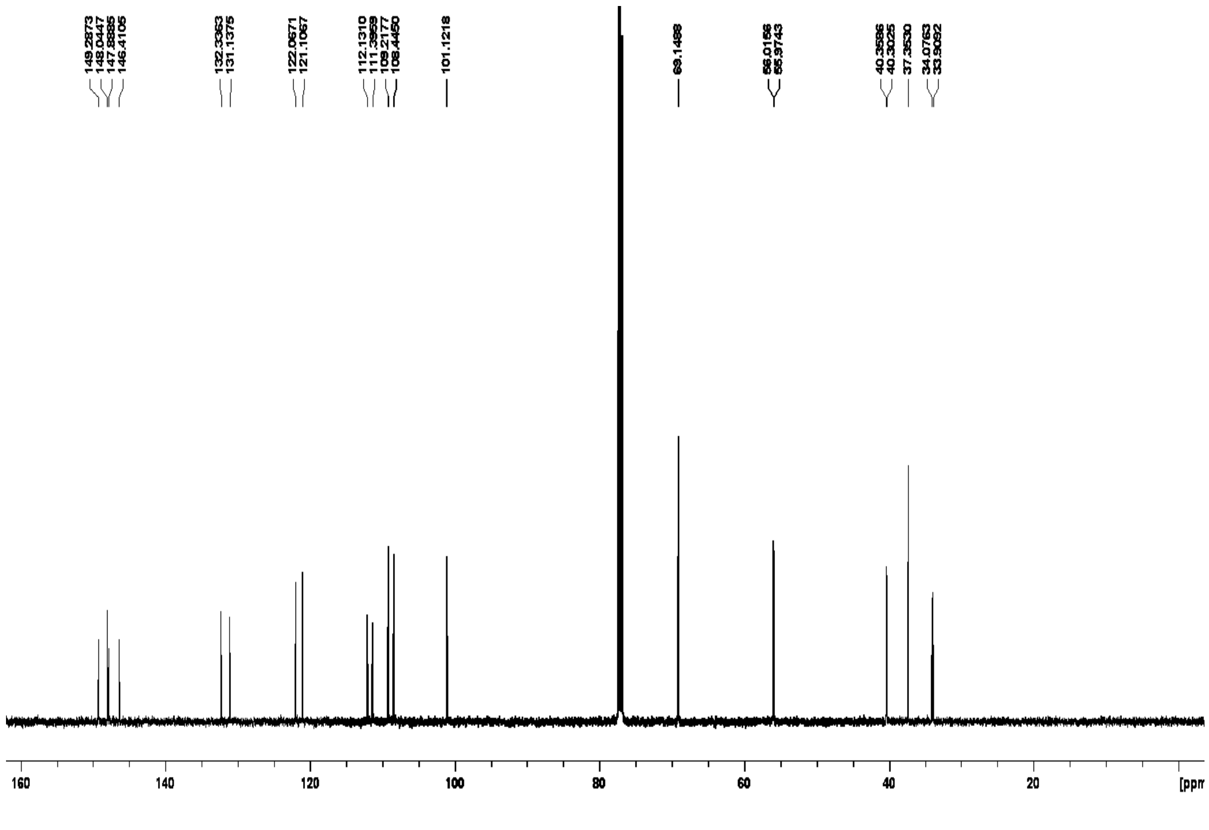

^{1}H-NMR ($CDCl_3$, 500 MHz) of compound **13**.

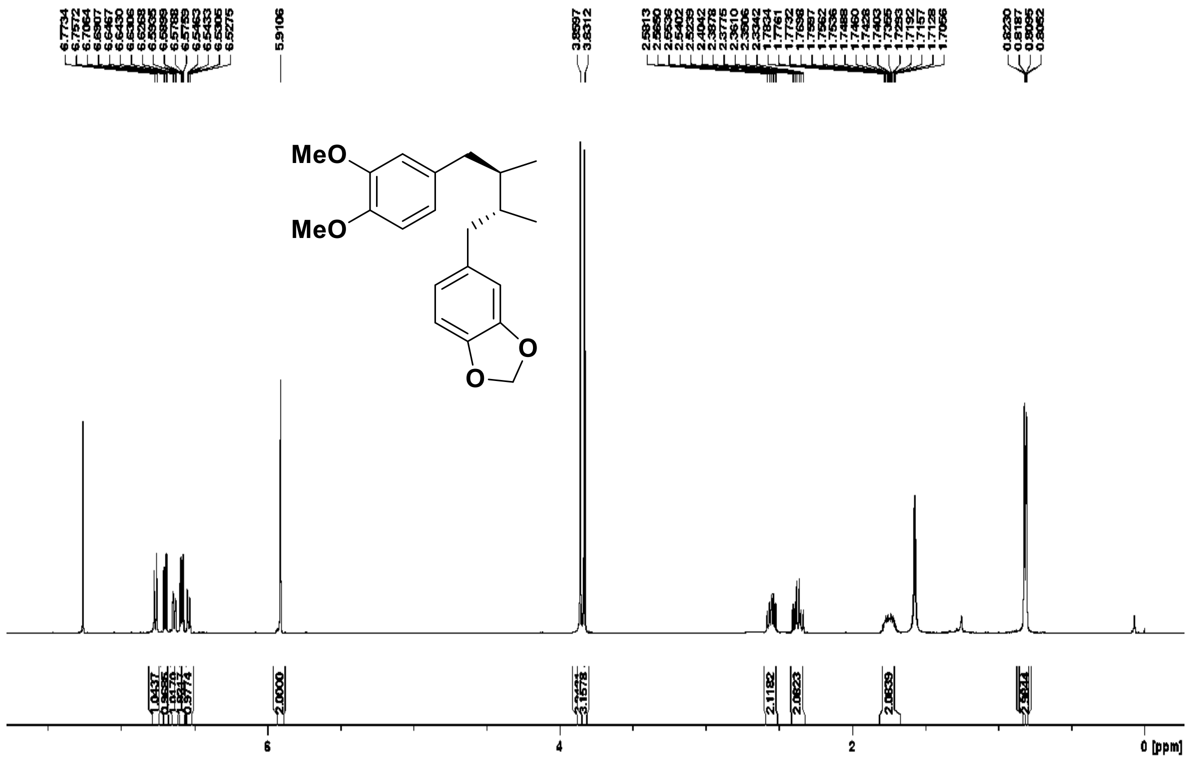

^{13}C-NMR ($CDCl_3$, 500 MHz) of compound **13**.

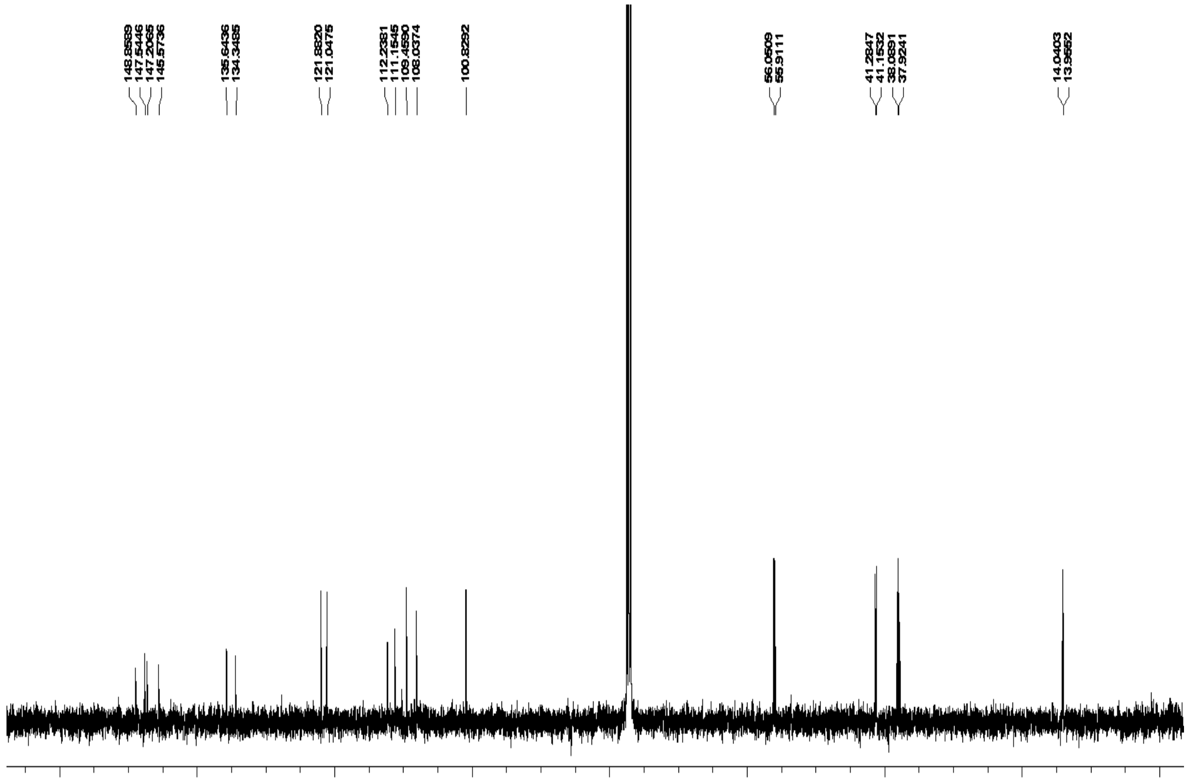

^{1}H-NMR (MeOD-d4, 500 MHz) of compound **14**.

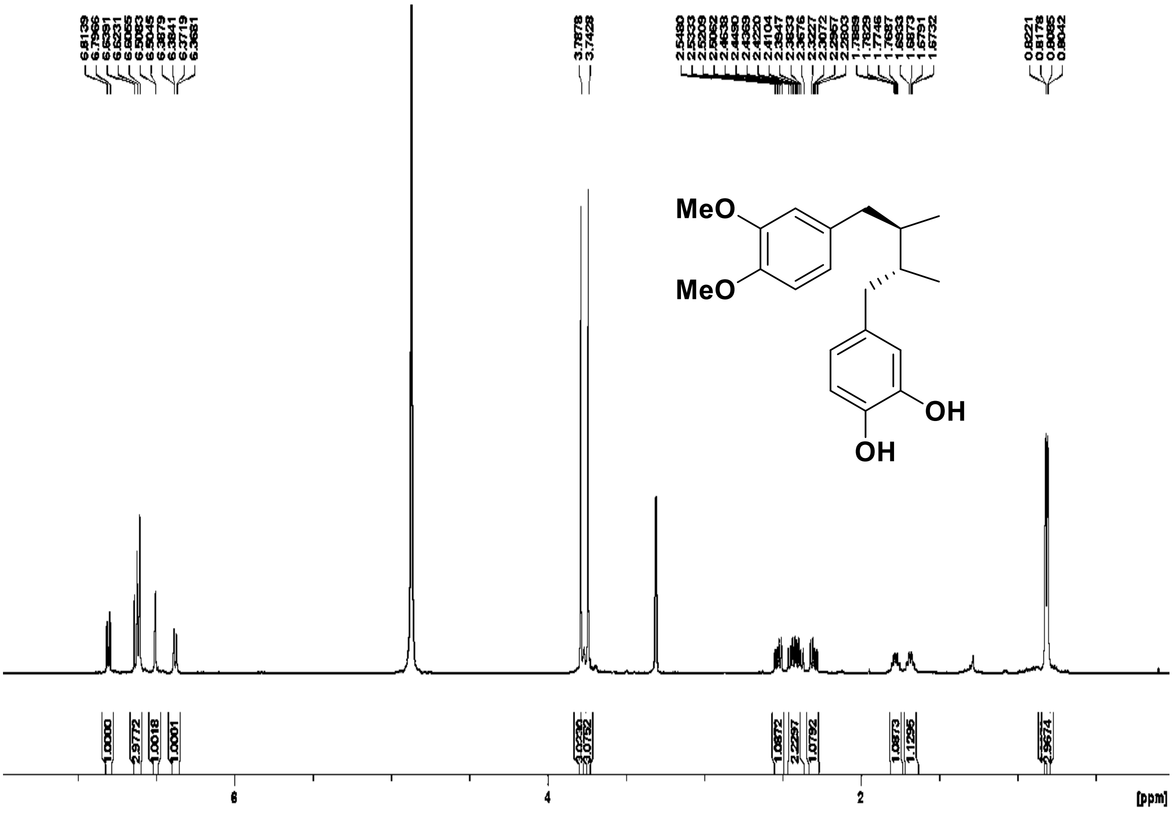

^{13}C-NMR (MeOD-d4, 500 MHz) of compound **14**.

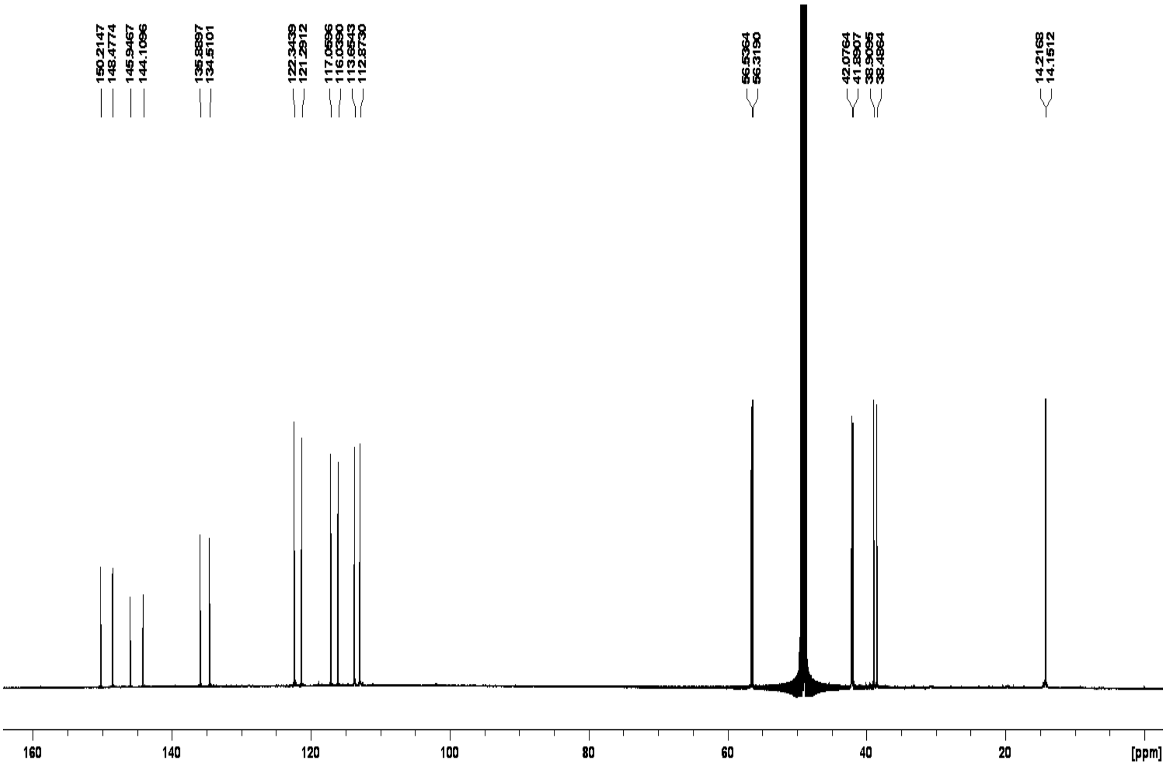

^{1}H-NMR ($CDCl_3$, 500 MHz) of compound **15**.

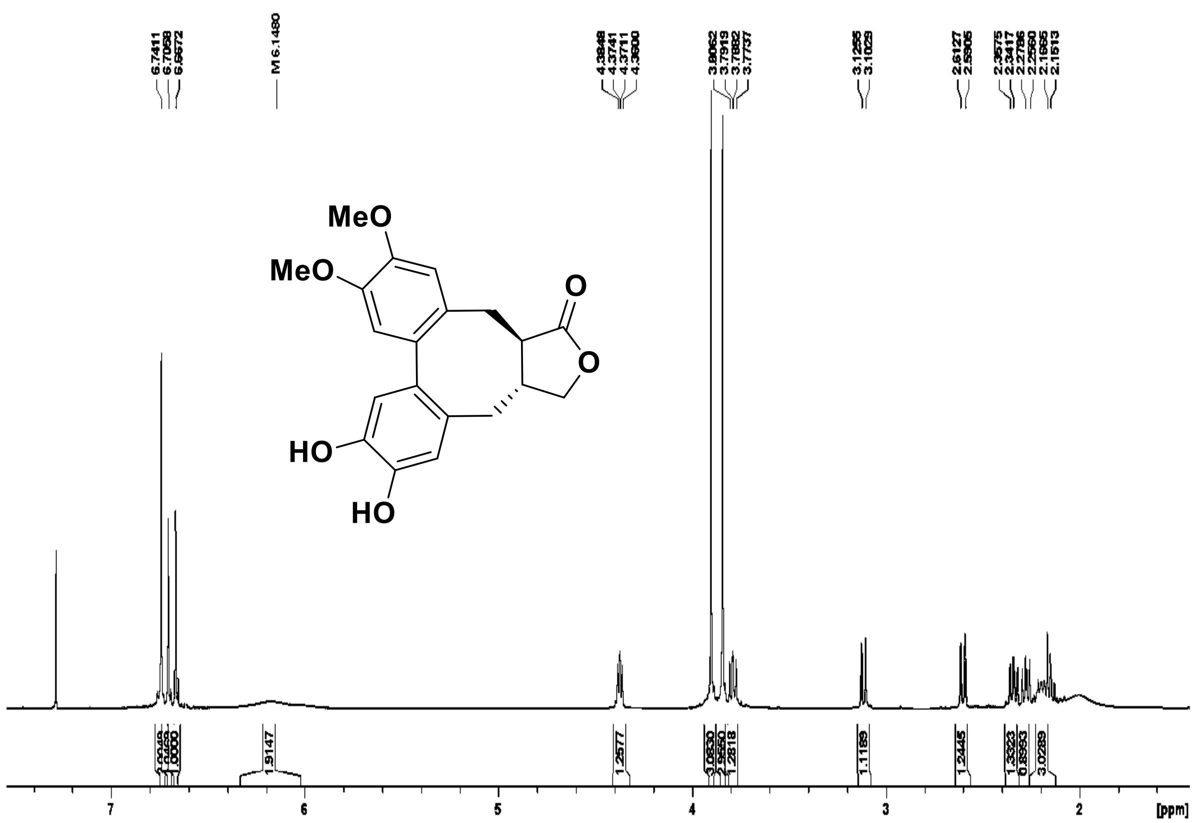

^{13}C-NMR ($CDCl_3$, 500 MHz) of compound **15**.

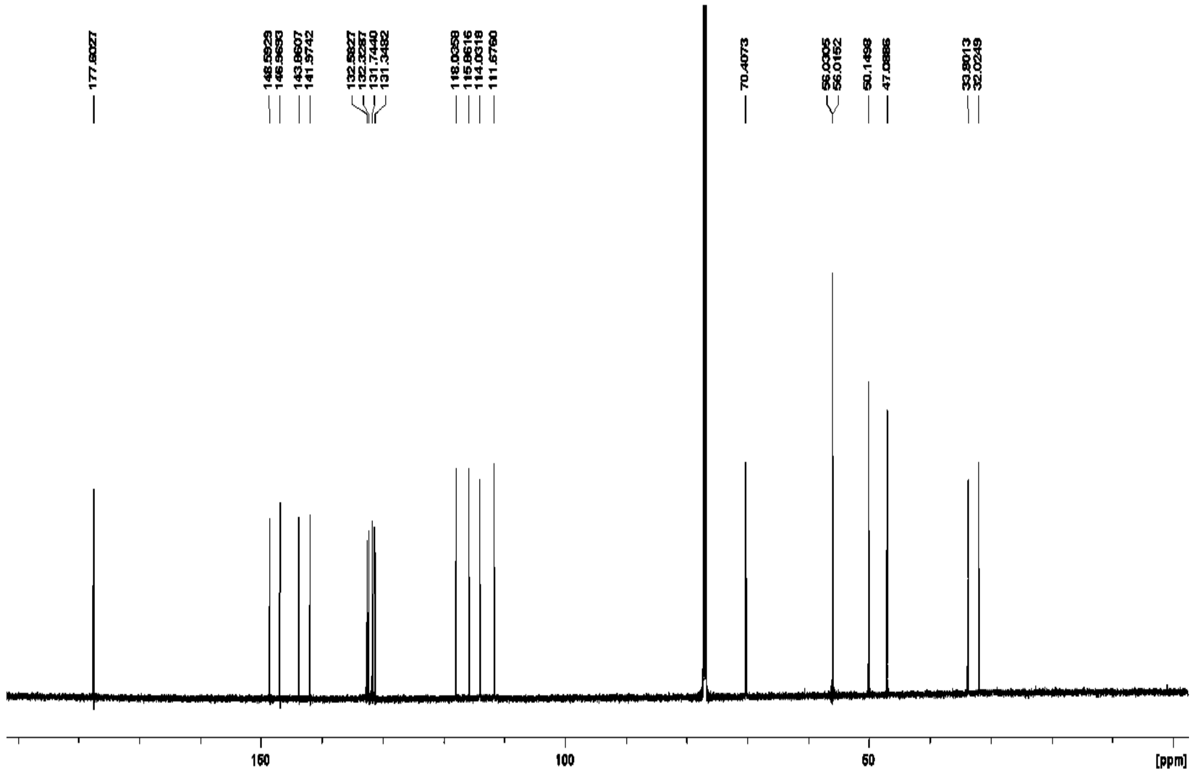

^{1}H-NMR ($CDCl_3$, 500 MHz) of compound **16**.

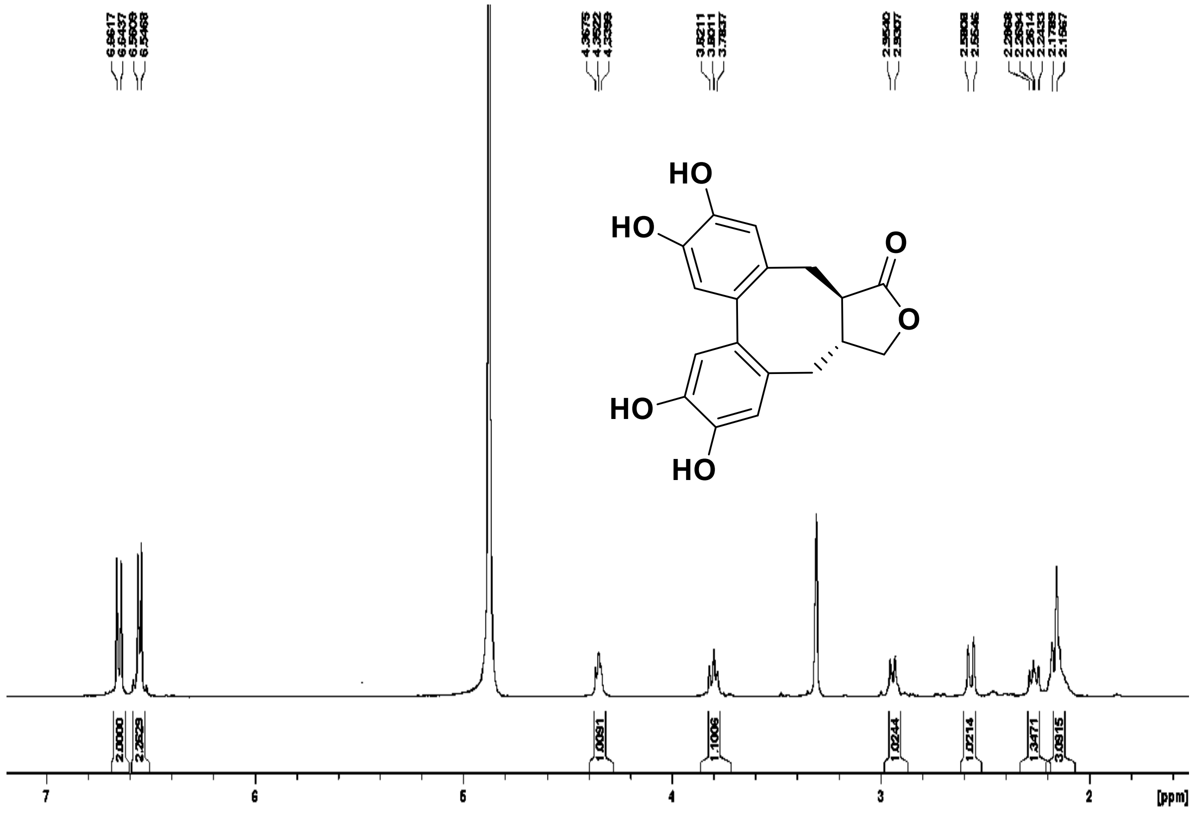

^{13}C-NMR ($CDCl_3$, 500 MHz) of compound **16**.

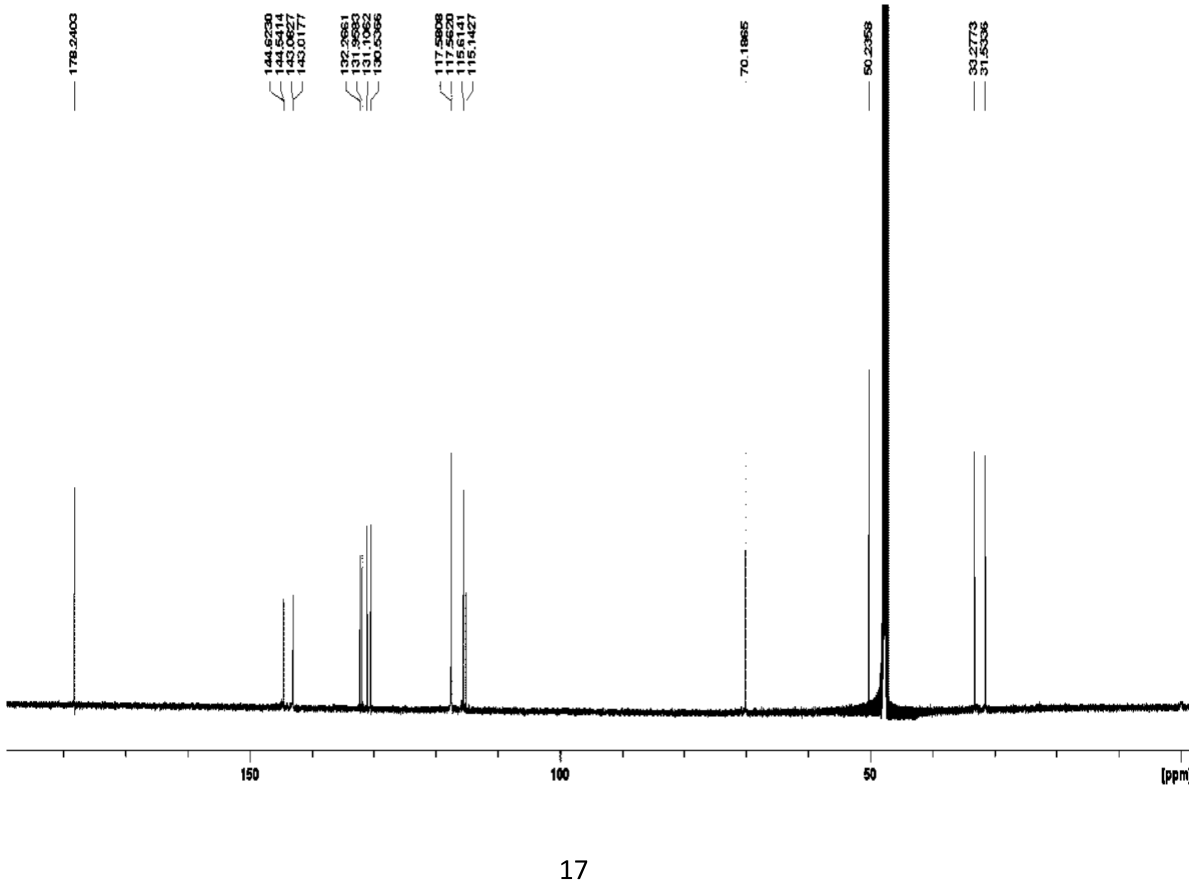

Supplement: Supplementary file 1 [file pharmaceuticals-15-00585-s001.zip › pharmaceuticals-1670817-supplementary.pdf]
